# Tempo and Mode of Gene Duplication in Mammalian Ribosomal Protein Evolution

**DOI:** 10.1371/journal.pone.0111721

**Published:** 2014-11-04

**Authors:** Asav P. Dharia, Ajay Obla, Matthew D. Gajdosik, Amanda Simon, Craig E. Nelson

**Affiliations:** University of Connecticut Department of Molecular and Cell Biology, Storrs, Connecticut, United States of America; University of Lausanne, Switzerland

## Abstract

Gene duplication has been widely recognized as a major driver of evolutionary change and organismal complexity through the generation of multi-gene families. Therefore, understanding the forces that govern the evolution of gene families through the retention or loss of duplicated genes is fundamentally important in our efforts to study genome evolution. Previous work from our lab has shown that ribosomal protein (RP) genes constitute one of the largest classes of conserved duplicated genes in mammals. This result was surprising due to the fact that ribosomal protein genes evolve slowly and transcript levels are very tightly regulated. In our present study, we identified and characterized all RP duplicates in eight mammalian genomes in order to investigate the tempo and mode of ribosomal protein family evolution. We show that a sizable number of duplicates are transcriptionally active and are very highly conserved. Furthermore, we conclude that existing gene duplication models do not readily account for the preservation of a very large number of intact retroduplicated ribosomal protein (RT-RP) genes observed in mammalian genomes. We suggest that selection against dominant-negative mutations may underlie the unexpected retention and conservation of duplicated RP genes, and may shape the fate of newly duplicated genes, regardless of duplication mechanism.

## Introduction

### Gene Duplication and Genome Evolution

In 1970, Susumu Ohno hypothesized that gene duplication provided the raw material required for the diversification of gene function. It is now appreciated that gene duplication and loss is a dynamic process that has given rise to many large gene families critical to the evolution of complex organisms [1]. Recent data reveal that lineage-specific expansion and contraction of gene families is more rapid than previously appreciated, and is responsible for major differences in gene family size between closely related mammalian genomes [2]. These differences are likely to have made major contributions to the divergence of mammalian lineages and to human evolution [3], [4]. For this reason, understanding the forces that lead to the retention or loss of duplicated genes in complex genomes is fundamental to understanding genome evolution, and particularly to the evolution of complex organisms.

### Scales, Mechanisms, and Outcomes of Gene Duplication

Duplications occur at all genomic scales, from a single nucleotide to the entire genome, and vary greatly in frequency. Depending on the nature of the duplication, these events can have a positive, negative or neutral effect upon an organism. For example, duplications involving a single gene or set of genes can be associated with enrichment for essential functions, while large scale duplications can be associated with important evolutionary transitions, major leaps in development, and the adaptive radiation of species [5], [6]. Many physical mechanisms can give rise to duplication events: (1) whole-genome duplication (WGD), (2) tandem duplication (i.e., unequal crossing-over), (3) duplicative transposition, and (4) retrotransposition. WGD, tandem duplication, and duplicative transposition are DNA-mediated events, while retrotransposition is the Reverse Transcriptase (RT)-mediated insertion of a cDNA into the host genome. A WGD is believed to have occurred in yeast [7], and several have been inferred in the teleost lineage [8]; while the last WGD believed to have occurred in the mammalian lineage took place before the emergence of modern mammals [9], [10]. Compared to these very large scale and rare events, duplicative transpositions and tandem duplications are likely to drive much of the duplication and loss giving rise to complex gene families. These are DNA-mediated processes that preserve varying amounts of the source gene's intron-exon structure [4]. Often, depending on the scale of the duplication, varying amounts of the intergenic regulatory DNA flanking the duplicated gene is also transferred, increasing the likelihood of a functional duplicate being created [11]. In contrast, retrotransposition is a process whereby a spliced mRNA transcript is reverse-transcribed into DNA and randomly re-integrated into the genome, creating a copy lacking introns and the promoter and enhancer elements of the source gene [12]. Such retrogenes have traditionally been regarded as non-functional. However, recent studies have shown that rampant retrotransposition can create genes that function as protein-coding genes [13] and small RNA's [14], and which can have dramatic phenotypic consequences [15]. Recent interest in retrotransposition is highlighted by the identification of several functional retrogenes, such as *Fgf4* and *c1orf37-dup* in mammals [16], [17], and suggests that retroduplicaton may be a more important force in the evolution of complex gene families than has been widely appreciated.

### Retroduplication in the Mammalian Genome

Previously, we reported that conserved retroduplicates are widespread in mammals, representing half of all gene duplicates under purifying selective pressure [18], [19]. In addition, we noted that individual gene families have a strong tendency to evolve via DNA-mediated or RNA-mediated duplication, but not both. Developmentally important classes of genes, such as transcription factors, which often require large amounts of regulatory information to function properly, tend to evolve through DNA-mediated events. However, gene families involved in metabolic processes, such as protein synthesis, evolve primarily through RNA-mediated duplication. In fact, we reported that ribosomal protein (RP) genes are the largest class of conserved, retroduplicated genes in mammals [18]. While it is not surprising that the highly abundant ribosomal protein transcripts appear to be more frequently captured by retroviral reverse transcriptase than less abundant transcripts, it is intriguing that the slowly-evolving, highly-conserved ribosomal proteins have hundreds of intact duplicates in the genome.

### Known Examples of Retrotransposed Duplicates

One of the most prominent examples of a RNA-mediated duplicate is Fgf4, a retrogene associated with the breed-defining chondrodysplasia in domestic dogs [15]. A human-specific example is the C1orf37-duplicate, derived through retrotransposition after divergence of human from chimp expressed selectively in certain human tissues, such as brain. It is suggested to encode a novel transmembrane protein [20]. Similar examples include TRMT12 retrogene [21], IMP3 gene [22], [23] and other such retrogenes (see [24]–[28]). The majority of the aforementioned RT genes follow the convention that most retrogenes are in a state of “relaxed” selection. The molecular evolution of retrogenes is selectively neutral, allowing them to freely mutate, giving them a chance to be inactivated or positively selected, while parental genes remain subjected to purifying selection [29], [30].

### The Mammalian Ribosome and Ribosomal Proteins

The ribosome is an ancient molecular machine that is responsible for production of protein in all living cells. The mammalian ribosome consists of 79 RPs and four rRNAs. RPs play a central role in protein synthesis, are expressed at high levels, and evolve very slowly [31] showing strong conservation across the three domains of life [32]. Proper ribosomal biogenesis requires equimolar production of all RPs and rRNAs [33], [34]. These transcriptional regulatory constraints have been extensively elucidated in various studies [35], [36], along with the evidence that different ribosomal protein promoters exhibit equipotent strength [37]. Additionally, a strict copy number constraint is also observed as the majority of full length RP's have been shown to be single copy genes [38].

Due to the necessity of protein synthesis in any living cell, and the complexity of ribosome structure and assembly, it is perhaps unsurprising that mutations in ribosomal genes almost inevitably lead to pathological conditions such as Minutes in Drosophila [39] and Diamond-Blackfan anemia (DBA) in humans [40], [41]. Despite, or perhaps because of, their stringent conservation, the evolution of RPs in vertebrates is relatively understudied.

### Known Examples of Mammalian Ribosomal Protein Duplicates

One of the most recently published DNA-mediated ribosomal protein (DD-RP) gene duplicates is RPL22L1, paralog of ribosomal protein RPL22. These mouse paralogs play essential, distinct, and antagonistic roles in hematopoietic development [42]. Another known rodent-specific RT-RP duplicate is Rps23rg1, a gene originating from a retrotransposition of s23 mRNA that encodes proteins that decrease Alzheimer's β-amyloid level and tau phosphorylation [43]. There is also evidence for a ubiquitously expressed RT-RP duplicate, Rpl36al, and testis-specific RPL10L duplicate that have been implicated in compensation for the reduced dosage of X-linked RP genes [44]. As mentioned in a previous section, RPL3L is a DD-RP duplicate that has been observed to be highly expressed in a group of tissues where parent RPL3 has very little expression, exhibiting a potential functional role [45].

### Focus of this study

In this study, we address several questions about gene duplication and the evolution of RPs. Many research groups have studied the dynamics of gene duplication in RPs in non-mammalian systems such as yeast [46]. In addition, some research has focused on individual human ribosomal proteins and their duplicates (eg: RPS19 and RPL7a) or comparative analyses between mammals [47]–[50]. To date, however, no study has encompassed all 79 mammalian RPs in a large set of mammalian genomes. In order to fill this gap and more thoroughly annotate RP gene duplication events during mammalian evolution, we created a pipeline that utilizes local synteny and conserved intron content to (1) identify each duplicated RP gene in eight mammalian species, (2) place each duplication event within the mammalian phylogeny, (3) discriminate between RNA- and DNA-mediated duplications, (4) estimate the degree of purifying selective pressure exerted on every duplicated RP gene, and (5) determine whether each duplicated RP gene copy exhibits evidence of expression.

## Materials and Methods

### Ribosomal Dataset

**Figure 1:**
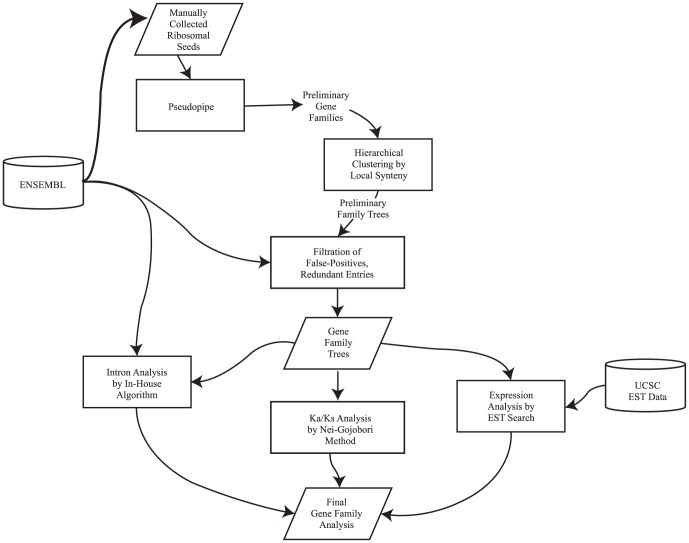
Pipeline for ribosomal protein family analyses. Protein sequence for all parental ribosomal proteins were collected manually from Ensembl62. These were input to tBLASTn against whole genomes to capture all putative duplicates. The resulting duplicates were processed by Pseudopipe to determine the mechanism of duplication (DNA or RNA) and the fate of the duplicate (intact or pseudogenized). We then utilized our in-house pipeline steps of hierarchical clustering by local synteny^3^ in order to build our gene family trees after filtering false-positives and redundant entries. Final gene family analyses were conducted in 2 steps: 1) calculating the selective pressures on all gene duplicates using the Nei-Gojobori method against the species- and family-specific seed protein via an exon-based reconstruction, and 2) checking for expression signatures via EST analyses using the UCSC genome browser EST track for both human and mouse.

Seventy-six ribosomal protein (RP) sequences from nine species [human, chimp, monkey, mouse, rat, dog, cow, opossum, and chicken (outgroup)] were manually collected from Ensembl 62 [51]. Three RPs were excluded due to annotation issues. When a single gene encoded multiple transcripts, the longest was used. These protein sequences served as seed sequences, or input, to the pipeline (Figure 1).

### Extraction of Gene Family Members

RP seed sequences were submitted to tBLASTn against donor genomes to capture as many putative duplicates of the seed gene as possible. Each resulting putative duplicate was processed using Pseudopipe [52] to determine the mechanism of duplication (DNA- or RNA-mediated) and the fate of the duplicate (intact or pseudogene). The default Pseudopipe filters for tBLASTn hits (E-value cutoff ≤10^−4^ and identity and identity ≥40%) were used to define putative duplicates. Ambiguous duplicates, where the duplication mechanism was not confirmed, were resolved using an intron comparison algorithm [18], which compares intron/exon structure within a group while accounting for exon fusions and large insertions in exonic regions. These methods generated a set of RP superfamilies that consist of both protein-coding genes and related pseudogenes.

### Identification of Duplications and Phylogenetic Analysis

Orthologous and paralogous relationships were determined using local synteny and a hierarchical clustering algorithm described in Jun et al, 2008 [18], [19]. A local synteny score was assigned to all gene pairs based on the homology of genes (three upstream and three downstream) neighboring the two query genes. Pairwise synteny measures were obtained for all members of a gene family. The output generated based on these scores was used to construct phylogenetic trees in Newick format, representing the history of duplication in each family. Parsimony [53] was used to assign each inferred duplication event to a specific branch of the species tree [54], [55]. ‘Tube’-style phylogenetic trees for 74 mammalian RP genes were used to illustrate the history of DNA/RNA-mediated duplications across various evolutionary time periods (ancient vs. lineage specific) (See Appendix S1 for all trees).

### Conservation and EST Analyses

Using exon-based reconstruction and the Nei Gojobori method, Ka/Ks ratios for all members of a gene family were calculated against the seed proteins. The putative exon-intron structures of duplicates were generated with an in-house algorithm, using these seed proteins. Results were then filtered based on p-values (<0.1) and the fraction of the parental gene represented by each duplicate (>65%). Pairwise distances using ClustalW were also calculated as an added metric to evaluate sequence identity and account for all nucleotide level substitutions. Additionally, we also determined branch-wise omega values for 28 ribosomal protein families with following parameters, model  = 2 & Nsite  = 0, using codeML in PAML 4.7 [56]. In order to confirm the selective pressures, standard codon models M0, M1a, M2a were fitted to the data set with codeML. We used likelihood ratio tests (LRT) to determine the relative fit of the hierarchically nested models. Log likelihood ratio test statistic is 2Δℓ = 2(ℓ1–ℓ0), where ℓ1 is the log-likelihood of the model corresponding to the alternative hypothesis and ℓ0 represents the log-likelihood corresponding to the model used as null hypothesis. These values were compared with a chi-squared distribution in which the difference between the number of parameters of both models provides the degrees of freedom (df) [57], [58]. Log likelihood values and parameter estimates are detailed in the results section and supplementary material.

In order to determine if duplicates were actively transcribed, human and mouse expressed sequence tags (EST) were mined from the UCSC genome browser EST. ESTs that mapped to multiple locations that showed less than 95% identity or 95% fraction length were discarded. Additionally, EST presence & absence calls were also made using data mined from Bgee database for annotated duplicates in our dataset [59].

## Results

### 76 Ribosomal Protein Family Member Analyses

**Figure 2:**
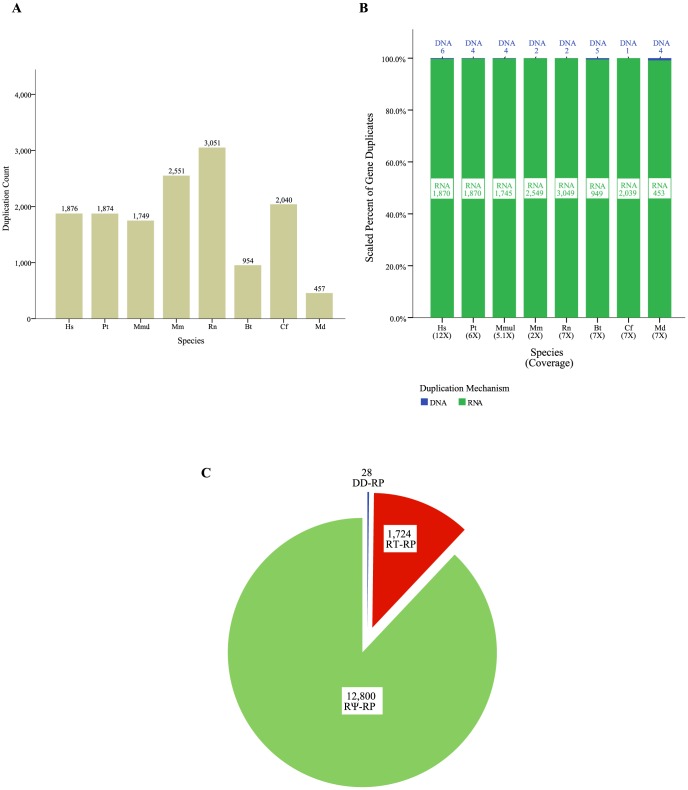
RP gene duplicates in 8 mammalian genomes. A) Distribution of duplication events in 8 mammalian genomes. B) Assessment of coverage or species-specific bias in ribosomal protein gene duplicates. C) Representation of DNA and RNA-mediated duplications in RP gene families. Abbreviations: Hs, *Homo sapiens* (human); Pt, *Pan troglodytes* (chimpanzee); Mmul, *Macaca mulatta* (Rhesus macaque); Mm, *Mus musculus* (house mouse); Rn, *Rattus norvegicus* (Norway rat); Bt, *Bos taurus* (cattle); Cf, *Canis familiaris* (dog); Md, *Monodelphis domestica* (gray short-tailed opossum); Gg, *gallus gallus* (chicken).

The first step of our pipeline identified all detectable duplicates of RP genes in eight mammalian genomes. RP families included 14,552 gene duplicates in the eight genomes analyzed: human, chimp, monkey, mouse, rat, dog, cow, and opossum (Figure 2A). Although data in figure 2A include duplicates with shared ancestry, the counts for each species represent the number of duplicate genes present in each extant species. To determine if sequencing coverage had a significant impact on our detection of RP gene duplicates, we compared the depth of sequence coverage in each species' genome to the number of duplications recovered in that species. We found no significant correlation between the number of duplications and genome coverage (Pearson's r = −0.353, p = 0.391, Figure 2B, S2). We also tested for bias in duplication types in each species and found no species-specific bias in duplication mechanisms. As we found significant association between species (p = 6.07e-17, two-way chi square test, Figure2B), all species were grouped for subsequent analyses.

**Figure 3:**
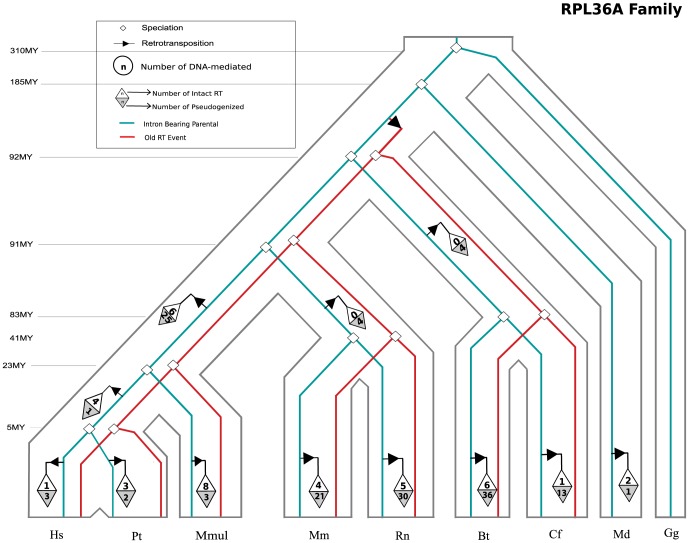
Example of the inferred evolutionary history for duplications of the ribosomal protein gene Rpl36a. Grey outlined tube tree represents the species tree that includes 8 mammals and chicken. Parental intron-bearing gene (in blue). RT-RPs (clear triangles), RΨ-RPs (grey triangles). An RT-RP duplicate generated from one of these events, Rpl36al (in red, at the base of the mammalian lineage on the branch between LCA with opossum and the other mammals) is conserved in all descendent species. All the 74 ribosomal protein gene family history trees are attached as supplementary material.

Next we assessed the fate of each duplicate. Of the 14,552 duplication events detected, only 28 of these gene duplications are DNA-mediated (DD) events; the remainder (99.8%) are RNA-mediated (RT) duplications. Approximately 88% of RNA-mediated duplications are pseudogenes (12,800 duplicates), while 12% are intact (1724 duplicates, Figure 2C). A data table listing all RP gene duplicates recovered by our pipeline can be found in Table S1. We also examined every ribosomal protein gene's duplication history and evolutionary trajectory in the context of the encompassing species tree. One example of the resulting information is shown in Figure 3, for the ribosomal protein gene RPL36A. All 74 ribosomal protein gene family history trees are attached in supplementary material. Hereafter, all the intact RNA-mediated ribosomal protein gene duplicates will be referred to as RT-RPs, intact DNA-mediated copies as DD-RPs and RNA-mediated pseudogenes as RΨ-RPs. Leveraging previously published data by Jun et al; we observed a clear overrepresentation of RT-RPs among 8,872 gene families analyzed (Figure S1).

### The Fate of Ribosomal Protein Duplications over time

**Figure 4:**
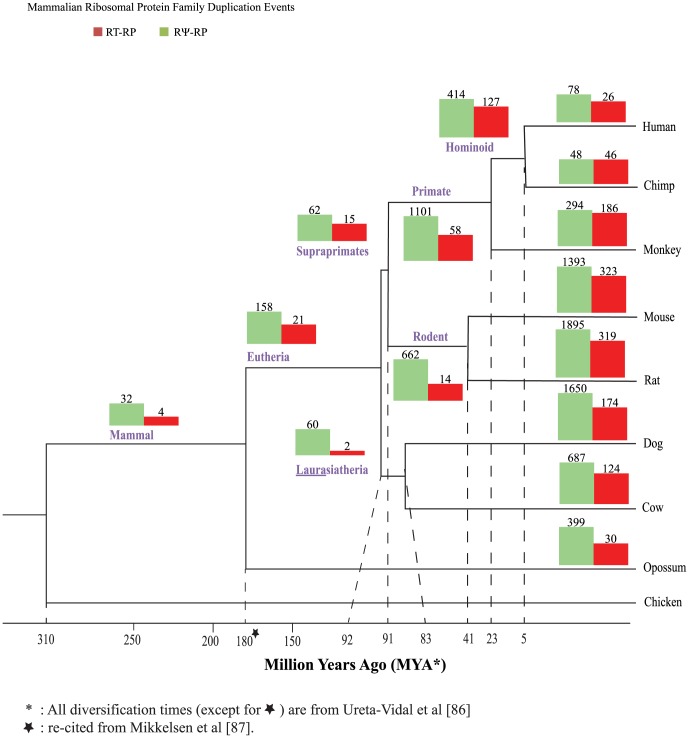
Ribosomal Protein Family duplication events based on age. All RP gene duplication events are displayed for 8 mammalian species. The bar charts at all speciation nodes show events classified by fate of duplication. The duplication counts on the bar charts are log normalized. RT-RPs are shown in red and RΨ-RPs in green. DD-RPs are not shown due to a very small sample size. The numbers above the bar charts represent the total number of gene duplication events at that speciation node. Age is marked at the bottom of the tree in millions of years (age estimates from [55], [103]).

In the second step of our pipeline, we determined the probable location of each RP duplication event in evolutionary history of these eight species, and distinguished between RNA- and DNA-mediated duplication events (Figure 3; [18]). Based on our methodology, Figure 4 clearly shows that the majority of detectable duplications have occurred during recent mammalian evolution: 100 million years ago (MYA) or more recently. However, a significant number of duplications date between 100–300 MYA. The majority of RP gene duplications older than 90 MYA result in RNA-mediated pseudogenes (RΨ-RPs) (190), though some events (25) are intact RNA-mediated duplications (RT-RPs), and a very small number (4) are linked to intact DNA-mediated duplicates (DD-RPs) [data not shown for DD-RPS due to small sample size]. It is important to note that many of the more ancient duplications detected represent incomplete clades; therefore we infer a considerable amount of gene loss. However, our inability to detect these genes may also be due to loss of synteny or other limitations of our pipeline.

The majority of duplicates (N = 13,588) observed in our dataset are young (91 MYA or younger). However, a few RT-RPs and DD-RPs have been conserved in all (or most) of the eight mammalian species analyzed (see the base of the tree in Figure 4).

### Analysis of Selective Pressure Acting on All Ribosomal Gene Duplicates

**Figure 5:**
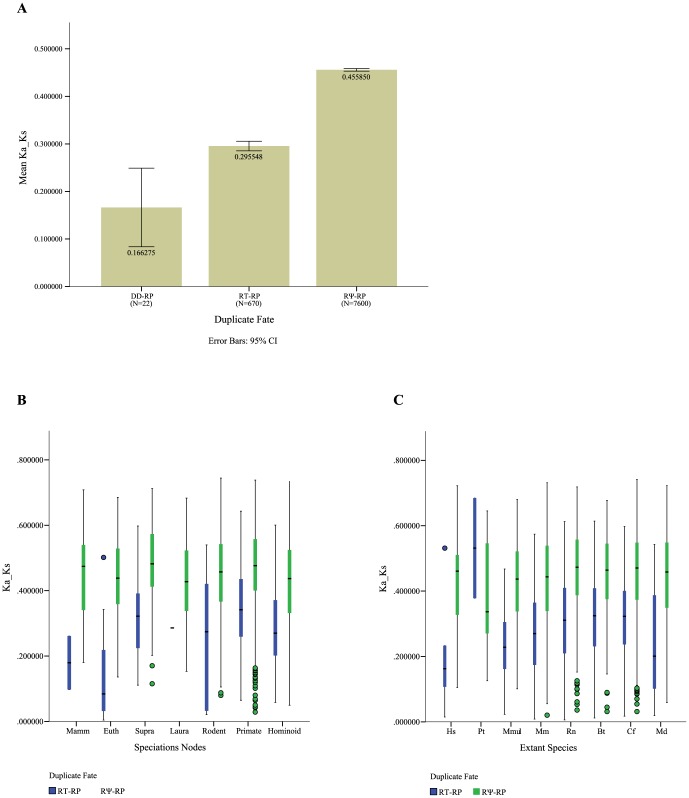
Selective Pressures on Ribosomal Protein Gene Duplicates. A) Mean Ka/Ks ratios were calculated for all classes (DD-RPs, RT-RPs and RΨ-RPs) of RP gene duplicates using the Nei Gojobori method. Results were then filtered based on p-values (<0.1) and the fraction of the parental gene represented by each duplicate (>65%). Error bars represent 95% confidence interval. B) Box and whisker plots for RT-RPs (blue) and RΨ-RPs (green) were generated for inner speciation nodes and C) Extant Species. DD-RPs were not included in the analyses due to small sample size (N = 3).

**Figure 6:**
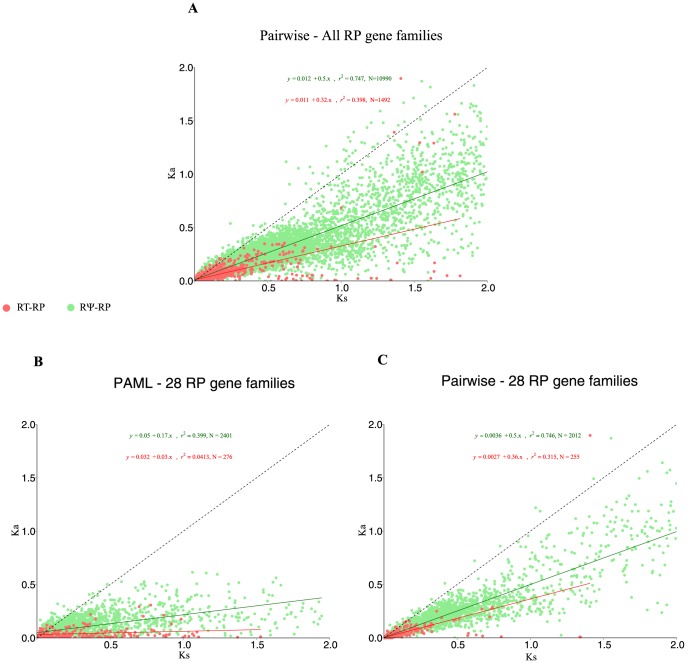
Scatterplots for pair-wise and branch-wise Ka against Ks values show that both methods capture the strong selective pressure acting on the RP gene duplicates. Plot of Ka against Ks for RP families with branch-wise and pair-wise methods. A) Distribution for all 76 RP families using the pair-wise selective pressure calculation method. Red dots represent RT-RPs and green dots represents RΨ-RPs. The black solid line represents Ka  =  Ks and the red & green line are the best line of fits for the distribution of RT-RPs and RΨ-RPs respectively. B) Distribution for 28 RP families analyzed by codeml program in PAML. C) Distribution of the aforementioned 28 families from PAML analysis using the pair-wise method.

To gain insight into the forces shaping the fate of these RP gene duplicates, nonsynonymous/synonymous substitution rates were evaluated using pair-wise and branch-wise methods (see Methods section and [13], [60], [61]. For the pair-wise method, we observe that DD-RP dups and RT-RP dups have mean Ka/Ks values of 0.166 (95% CI 0.083, 0.248) and 0.295 (95% CI 0.285, 0.305) suggesting that they are under strong purifying selective pressure. RΨ-RP's were under relatively less purifying selective pressure with a mean value of 0.455 (95%CI 0.453, 0.458) (Figure 5A). In order to avoid false positives with Ka/Ks >1, we did not include cases that had a very low Ka and Ks values. Calculation of pairwise DNA sequence distances reveals that the mean sequence distance for DD-RP duplicates was 0.091 (95% CI 0.062, 0.118), for RT-RP duplicates was 0.0059 (95% CI 0.062, 0.118) and for RΨ-RPs was 0.172 (95% CI 0.169, 0.173). This corroborates the evidence from the Ka/Ks analysis suggesting that these sequences are under strong selective pressure (Figure S3). Next we compared selective pressures on all RT-RP duplicates of various ages in each lineage. (DD-RPs were not included in this analysis due to the very small dataset.) Box-Whisker plots (Figure 5B and 5C) showed that RT-RP duplicates at all speciation nodes, irrespective of their age or lineage, are under strong selective pressures, as determined by Ka/Ks values. However, chimp (Pt) values seem to be an exception, likely due to a small sample size (Figure 5B). The trends appeared similar for all RΨ-RPs as the median Ka/Ks values are similar (∼0.45) for all ages. Additionally, we also provide scatterplots for all speciation nodes to confirm the strong selection on all RP duplicates irrespective of age (see figures S6 and S7). While pairwise Ka/Ks calculations are computationally rapid and provide a good screen for selective pressure, especially within a gene family, for added support we wanted to cross-check our estimates of selective pressure using branch-specific omega values. To do this we used PAML to calculate branch-specific omega values for a sub-sample of 28 RP gene families (see Table S2). An example RP gene tree with all PAML branch-specific omega values is shown in Figure S4. Using this approach we obtained Ka/Ks values for RT-RP duplicates (mean  = 0.162, CI 0.137, 0.188), and for the RΨ-RPs (mean  = 0.357, CI 0.347, 0.367). As previously mentioned, to avoid false positives with Ka/Ks >>1, we excluded cases with very low Ka and Ks values. Both pairwise and PAML-based estimation methods confirm the strong purifying selective pressure acting on RT-RPs (Ka/Ks <0.3) and a slightly lower pressure on RΨ-RPs (Ka/Ks <0.5). As evolutionary pressure is often time dependent, we also plotted Ka against Ks estimated by both pair-wise (Figure 6A) and branch-wise (Figure 6B) methods. As expected the branch-wise method estimates higher divergence, as seen by the large distribution of Ks values (Figure 6B) compared to pairwise method. The influence of strong purifying selection over time is readily observed in Ka values for both methods as the data points of RP-RTs are compressed near the origin relative to RΨ-RPs, which have a much wider distribution (Figure 6).

In order to further confirm the nature of the selective pressure acting on our RP-RTs and RΨ-RPs, we also used different codon-substitution models developed by Nielsen and Yang [62] and Yang et al. [58]. Random-site models M0, M1a and M2a which assume variation in ω among sites but not among lineages were fitted to our data. The models used, parameter estimates and log-likelihood values are shown in Table S3. Table 1 shows the results of the LRT tests for these models. We applied the simplest of site-based models M0 [30], which assume a uniform ω ratio for all codons, to four random ribosomal genes namely Rps16, Rps18, Rpl14 and Rpl28. The estimated single ω value for each of these trees ranges from 0.22 to 0.35 (table S3). These values can be interpreted as an average of all lineages in the tree and over all sites in the protein. The low ω range obtained indicates a strong action of purifying selection in the evolution of ribosomal gene duplicates studied.

**Table 1:** Likelihood Ratio Test statistics (LRT) for random site models.

Genes		M1a^a^ vs. M2a^b^	M1a^a^ vs. fix_omega = 1^b^
**RPL28**	df	2	2
	2Δℓ	−150.153	1209.854
	P-Value	1	<0.001
			
**RPL14**	df	2	2
	2Δℓ	−70.308	2043.084
	P-Value	1	<0.001
			
**RPS16**	df	2	2
	2Δℓ	1024.072	2410.42
	P-Value	0	<0.001
			
**RPS18**	df	2	2
	2Δℓ	−1269.931	1332.272
	P-Value	1	<0.001

a Alternative model;

b null model; 2Δℓ = 2(ℓ_1_−ℓ_0_), df degrees of freedom.

To test if branch-specific omegas are statistically justified, we compared Model M1a (nearly neutral), which constrains Ka/Ks ≤1 but not positive selection (Ka/Ks >1) and M2a which allows for positive selective pressure. This comparison leads us to reject the nearly neutral model as seen in table 1. Our final comparison was model M1a vs. M0 with a fix omega  = 1 and we find out that model M1a fits our data better (p-value <0.0001). These results confirm that purifying selection is the predominant force acting in the evolution of ribosomal protein genes. Hence it further validates the Ka/Ks values obtained from both pair-wise and branch-wise methods.

### EST Analysis for human and mouse RP duplicate genes

**Figure 7:**
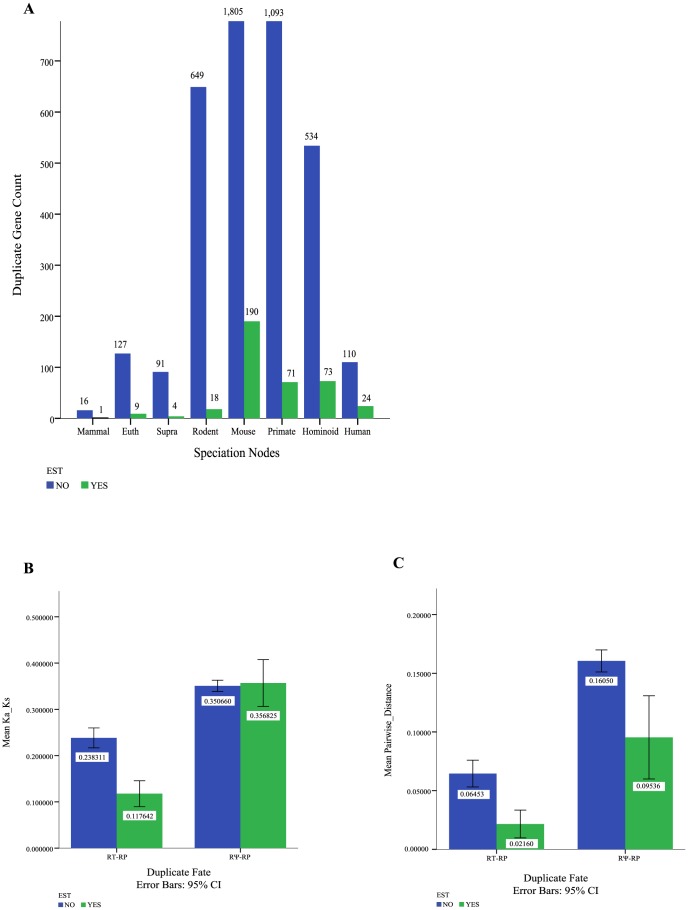
Human/Mouse EST Counts and evolutionary selective pressure. A) Counts of human and mouse genes with EST (green) versus NO EST (blue) at all speciation nodes were calculated. B) Ka/Ks values were calculated for RT-RPs and RΨ-RPs for gene duplicates with (green) and without expression (blue). C) Pairwise distances for RP-RTs and RΨ-RPs with and without expression.

EST data for human and mouse were mined in the final step of our analysis pipeline. Using very stringent constraints (see Methods), we found evidence of expression for approximately 8% of all human and mouse duplicates. It should be noted that in order to avoid false positives resulting from the strong sequence similarity between parent genes and duplicates, a large number of EST matches were filtered out, suggesting that our estimates of active transcription are likely underestimates. The majority of EST data results from duplicates arising along younger portions of the mammalian lineage (younger primate or rodent lineage or the mouse, hominoid and human branches (Figure 7A)). For example, 320 out of 335 ESTs are either primate-specific or mouse-specific.

Finally, we compared the selective pressure on all RT-RP duplicate genes with evidence of expression (in the form of EST matches) to those without matching EST data. DD-RPs were not analyzed due to the small sample size (N = 3). Expressed RT-RP duplicates exhibit significantly higher levels of purifying selection than their non-transcribed counterparts (mean Ka/Ks value of 0.12 (95% CI 0.09, 0.15) compared to 0.24 (95% CI 0.22, 0.26) respectively (Figure 7B)). However, no similar difference in selective pressure is observed between expressed RΨ-RPs, whose mean Ka/Ks value is 0.36 (95% CI 0.31, 0.41), and their non- transcribed counterparts (mean Ka/Ks value of 0.35 (95% CI 0.34, 0.36)) (Figure 7B). Similarly, pairwise sequence distances for these duplicated genes show that expressed RT-RP duplicates (mean pairwise distance of 0.02 (95% CI 0.01, 0.03)) have diverged less than intact non-transcribed duplicates (pairwise distance of 0.06 (95% CI 0.05, 0.07)) (Figure 7C). However, just as in the Ka/Ks analysis above, expressed RΨ-RPs have diverged less than non-transcribed pseudogenized duplicates (0.09 (95% CI 0.06, 0.13) compared to 0.16 (95% CI 0.15, 0.17) (Figure 7C). It is interesting to note that mean pairwise sequence distances are lower for pseudogenes with ESTs, suggesting purifying selective pressure prior to the pseudogenization event.

## Discussion

Here we provide a near-comprehensive study of ribosomal protein gene sequence evolution, duplication, and loss in eight mammalian species. We find that these highly-conserved and highly-expressed genes are, not unexpectedly, frequently duplicated by retrotransposition, and comprise the largest such class of genes in mammalian genomes. It is quite clear that RNA-mediated RP duplicates (14,524 out of 14,552 events) dominate RP gene families. There is rare evidence of an old DNA duplicate, RPL3L (see Figure S5 for family tree) that has been retained for function (see [36], [63]). However, the presence of only a very few such old duplicates and a complete absence of recent DD-RP duplicates, implies selection against the retention of DNA-mediated RP duplications. Negative selection against DNA-duplicates combined with the abundance of ribosomal protein gene mRNAs, and the observation that reverse transcription and transposition are more efficient on short GC-poor sequences like the ribosomal mRNAs [64], [65], likely explain the almost complete dominance of retroduplication events in the evolution of the mammalian ribosomal protein genes.

Less expectedly, we also find that many of these retrotransposed RP duplicates are under strong purifying selective pressure (N = 1,724), and that this pressure is greatest amongst transcribed RP retroduplicates, regardless of whether these duplicates have been pseudogenized or retain intact coding regions. As gene duplicates are often found to be under relaxed selective pressures [66]–[68], the strength of selective pressure we observe across RT-RP duplicates was unexpected. It was not immediately obvious to us why so many duplicates are under selective pressure when the parental ribosomal genes exist almost exclusively in single copy, when DNA-mediated duplications appear to be selected against, where RP transcript levels are tightly regulated for optimal fitness, and the duplications are occurring over a timeframe where ribosomal evolution is thought to be almost stationary. Indeed, we expected RT-RP duplicates to be evolving neutrally for exactly these reasons.

The precise combination of forces enabling the retention of duplicated genes in complex genomes leading to the formation of gene families has been a subject of much study [69], [70]. Several interesting studies have focused on the fate of ribosomal protein duplicates in non-mammalian lineages. RP duplicate fate after WGD events have been closely studied in yeasts and plants [71], [72]. RP duplicates have been shown to be retained to maintain gene dosage after WGD [6], [72]–[74]. But these retention events are not expected to affect the relative stoichiometry between RPs. However, the primary mode of duplication observed in the present study is RNA-mediated, small-scale duplications, which could result in severe stoichiometric imbalance. Additionally, it has been implied that RP duplicates after WGD's can be selected for defined functions like increasing levels of gene expression and divergence of gene function [75]. But evidence for this is not readily apparent in mammalian RT-RPs.

Population genetics suggests that duplicates should be lost long before adaptive forces can fix them in the population [76]. Many models have been forwarded that attempt to explain this apparent paradox and provide scenarios within which duplicated genes will be retained at the levels observed in many genomes (for an excellent review see [77]). In an attempt to understand the origin of the widespread selective pressure we observe on mammalian ribosomal protein retroduplicates, we focus this discussion on the ability of current models to account for this phenomenon.

### Can existing models account for observed number of conserved RP-dups?

#### Neofunctionalization

After assessing the current literature for existing retrogenes and ribosomal gene duplicates, we wanted to evaluate the gene duplication models. Gene duplication models for neofunctionalization, namely, the Dyhkhuizen-Hartl model, the Adaptation model, and the Adaptive Radiation model, predict that the rate of evolution after gene duplication will be accelerated in the duplicated copy and constrained in the original gene [67], [78]. However, these models fail to account for thousands of ribosomal retrogenes in our dataset which demonstrates that rather than experiencing neutral selection, the new copy is under stringent purifying selection. Moreover, while some extra-ribosomal functions for divergent RP duplicates has been observed these events appear to be very rare [79]. Therefore, neofunctionalization models appear unlikely to account for the very large number of conserved ribosomal protein gene retroduplicates in mammalian genomes.

#### Subfunctionalization

Subfunctionalization, and its most cited model, DDC does appear to account for the retention of some number of gene duplicates [80]. DDC postulates that the genetic drift and accumulation of mutations will cause the loss of specific subfunctions from each copy of the duplicated genes. Once one copy has lost an essential function, selection on that function in the other duplicate will be reasserted. Eventually the two copies preserve largely non-overlapping complementary functions and both must be maintained by selection [80]. This division of function can result from changes in the regulatory regions or the coding regions of duplicated genes, and is most often envisioned as a driving force for the divergence of gene expression [for example see [74]].

However, DDC seems an improbable model for retention of the ribosomal protein retrogenes due to the fact that rather than appearing to drift, the coding regions of these duplicates are under strong purifying selective pressure. Large numbers of degenerative mutations in the coding regions are not observed until after pseudogenization. Also, because RT-RP duplicates do not carry any regulatory information, the most likely scenario for DDC, the evolution of complementary regulatory regions is unlikely. In addition, EST signatures retrieved from our pipeline and a review of existing literature [81] suggests that retroduplicates typically have a much narrower expression profile compared to the ubiquitous expression patterning of their parents, while the parents never seem to lose ubiquitous expression, as would be expected under DDC. Hence, division of function in such a manner seems improbable for ubiquitously expressed ribosomal parental genes. Other subfunctionalization models like EAC [82], and specialization and gene sharing [83] require neutral selection on the duplicate copy [84] and are not consistent with the purifying selective pressure we observe.

#### Gene conservation

Gene conservation is another outcome that can be used to explain the retention of retrogenes. The primary gene conservation model that has been employed to explain gene retention is the dosage model, which posits that gene duplicates are retained in order to produce more of the same gene product [69]. In comparison, the dosage compensation model states that the gene duplicates can compensate for the activity of the source gene [85].

The RP genes are under strict transcriptional regulatory control to maintain equimolar ratio of ribosomal constituents [86]–[88], and changes in ribosomal protein levels, including overexpression, are often highly deleterious [89]. This point is confirmed by DeSmet et al. 2013 paper [90] as they suggest that retention of small scale duplications (SSDs) will result in the stoichiometric imbalance among protein complexes and that the dosage balance hypothesis would work for a WGD as relative ratios among subunits can be flawlessly maintained, which would not be the case with SSDs. Similar conclusions were drawn for SSDs, suggesting that they would be selected against in a highly connected protein network [91]. This suggests that retroduplications that alter gene dosage would be selected against, not favored. Another very important piece of evidence that argues strongly against the retention of RP-RT duplicates by dosage is the study conducted by Kittler et al and Gilsdorf et al. [92], [93]. In this study 34 ribosomal retrogenes (highly conserved old, new, intact and pseudogenized candidates) were knocked-down with no detectable phenotypic defects. However, knock-down of each of 70 parental RP genes had drastic phenotypic defects on the cells, with no evidence of retrogenes compensating for the loss of parental gene products (data obtained and analyzed from [94]). Previous work done on *Paramecium tetraurelia* [95], [96] discusses about dosage compensation affecting the short term retention rate of duplicate genes after WGD's, while maintaining stoichiometry. While they correctly predict selection against the retention of non-balanced duplicates, they do not predict the knock-down results obtained in mammalian RT-RPs discussed earlier.

#### Retention of RT-RPs cannot be readily explained

Due to the fact that RT-RPs are very abundant, are highly conserved, lack parental regulatory regions, and because changes in ribosomal gene dosage are strongly selected against, the retention of the RT-RP duplicates is not readily explained using current models of Ohno's three trajectories of dosage, subfunctionalization, and neofunctionalization [1]. Because of this, identifying the forces leading to the retention of these thousands of highly conserved, expressed duplicates in mammalian genomes is likely to require a new model for the retention of gene duplicates.

#### Dominant-Negative effects acting to preserve RT-RP duplicates

One factor not fully explored in most existing models for the retention of retroduplicated genes is the potential for dominant-negative effects of missense mutations on cellular processes. Mutant proteins can act in a dominant-negative fashion in a wide variety of ways [97]–[99], and this mechanism could account for the strong purifying selective pressure we observe on duplicated genes. Importantly, a dominant-negative mechanism does not require complementation, neofunctionalization, subfunctionalization, ubiquitous expression, or a selective advantage for the new copy. The acquisition of dominant-negative mutations in duplicates may represent a threat to the viability of an organism *via* expression alone. Thus, these gene copies will remain under purifying selection until they are inactivated (pseudogenized or transcriptionally silenced).

We suggest that dominant negative phenotypes may exert an immediate and strong purifying selective pressure upon any duplicated gene, with this pressure varying directly with the potential for the gene product to act in a dominant negative fashion [99]. The pairwise Ka/Ks values of less than 1 that we observe may be the average of a regime of intense purifying selective pressure, followed by drift after pseudogenization. In a recent study of flowering plants, De Smet et al., postulate a very similar idea that dominant negative model constrains genes to be maintained as single copies to avoid non-specific interactions [90]. Strongly conserved multiprotein complexes like the ribosome are the most commonly observed context for dominant negative phenotypes, but dominant negative phenotypes are not restricted to such multi-protein complexes, in fact, they are widespread [98]. Because selection against dominant negative alleles acts immediately upon newly duplicated genes, and serves to maintain gene products in a very restricted portion of protein conformational space, it likely facilitates the retention of duplicates by many of the models described above by increasing the half-life of functional alleles in the population and the exploration of the small local region of allowable variation in protein conformation. Liberles and coworkers have proposed similar models in the context of negative pleiotropy [91], [100]–[102], and have reached parallel conclusions on the impact of these bottlenecks in sequence space during evolution. To gain support for these models of gene family evolution, it will be important to functionally test the predictions of these models in experimentally tractable systems.

## Supporting Information

Figure S1 **Observed frequencies for RNA-mediated duplicates are much higher than expected frequencies in RP families.** Observed frequencies and expected frequencies (in brackets) shown for each speciation node for 5 mammalian genomes. The values were generated using data table created in Jun et al [68]. Observed frequencies for RP-RTs were derived from ribosomal families (Number of RP-RTs/Total Number of Duplicates in RPs) and expected frequencies for intact retroduplicates were derived from 8872 non-ribosomal gene families (Number of RTs in non-rp gene families/Total Number of duplicates in non-rp gene families). All diversification times are from Ureta-Vidal et al [54].
(EPS)

Figure S2 **No Species-specific bias seen based on duplicate fates in ribosomal protein gene families.** Distribution of duplicates (annotated by fate) across 8 mammalian species. DD-RPs, RT-RPs and RΨ-RPs are shown in blue, green and yellow respectively.
(EPS)

Figure S3 **High sequence conservation observed across all duplicate fates in terms of pairwise distances.** DD-RPs and RT-RPs were seen to be under comparatively stronger conservation than RΨ-RPs. Using pairwise distances instead of Ka/Ks ratios as seen in figure 4. Error bars represent 95% Confidence Interval.
(EPS)

Figure S4 **Gene Tree for RPL10A showing PAML branch specific omega values leading up to a clade.** An abridged Gene tree of RPL10A generated by parsimony-based syntenic method (see Methods). The branch specific omega values are listed at each node in purple. Ka/Ks values are represented at all leaves in green. The RT-RP duplicates and their omega values are highlighted in red.
(EPS)

Figure S5 **Reconstructed evolutionary history for ribosomal protein gene RPL3.** Tube Tree showing RPL3L which is a DD-RP that can be seen persistent in all mammals since its origination (green line). Refer to figure 3 legend for tree annotation.
(EPS)

Figure S6 **Scatterplots for pair-wise Ka against Ks values show a strong selective pressure acting on the RP gene duplicates in older clades.** Panel of Ka against Ks graphs for RP duplicates using pair-wise method. Each panel represents different clades for 8 mammalian genomes we studied. Red dots represent RT-RPs and green dots represents RΨ-RPs. The black dashed line represents Ka = Ks and the red & green line are the best line of fits for the distribution of RT-RPs and RΨ-RPs respectively.
(EPS)

Figure S7 **Scatterplots for pair-wise Ka against Ks values show variable levels of purifying selection acting on the RP gene duplicates across all lineage specific clades.** Each panel represents lineage specific clade of 8 mammalian genomes. Refer to figure S6 for more information on the graph. Refer to figure 2 for abbreviations.
(EPS)

Table S1 **Table representing all our RP gene duplicates in 8 mammalian genomes and outgroup chicken.** The clade_num represents the syntenic relationships between gene duplicates.
(XLSX)

Table S2 **Table for all RP families that have PAML branch-specific Ka and Ks values.** The clade_num represents the syntenic relationships between duplicates.
(XLSX)

Table S3 **Log-likelihood and parameter estimates generated from random-site models for RP genes.** P =  number of free parameters for each model, l =  log-likelihood value for each model.
(PDF)

Appendix S1 **74 RP gene trees with all annotated duplication events.**
(PDF)
