# Supplementary material for: Tempo and Mode of Gene Duplication in Mammalian Ribosomal Protein Evolution
Source: PLoS One. 2014 Nov 4;9(11):e111721. doi: 10.1371/journal.pone.0111721 (PMC4219774; doi:10.1371/journal.pone.0111721)
Supplement: Table S3 — Log-likelihood and parameter estimates generated from random-site models for RP genes. P = number of free parameters for each model, l = log-likelihood value for each model. (PDF) [file pone.0111721.s010.pdf]

Supplementary Table3: **Log-likelihood and parameter estimates generated from random-site models for RP genes**. P = number of free parameters for each model, ℓ = log-likelihood value for each model.

RPL28:

Models	p	ℓ	Estimates of parameters
**M0:one ratio**	1	-14664.881	omega=0.223
**M1a:nearly neutral**	2	-15243.079	p_0=0.0001,p_1=0.9999,<br>omega$_0$=0.0001, omega$_1$=1.0000
**M2a:positive selection**	4	-15168.003	p_0=0.0000,p_1=0.83519, p_2=0.16481<br>omega$_0$=0, omega$_1$=1, omega$_2$=3.39940
**M0, omega=1:fixed omega**	4	-15848.007	None

RPL14:

Models	p	ℓ	Estimates of parameters
**M0:one ratio**	1	-11320.898	omega=0.348
**M1a:nearly neutral**	2	-10997.396	p_0=0.7266,p_1=0.27331,<br>omega$_0$=0.18668, omega$_1$=1.0000
**M2a:positive selection**	4	-10962.242	p_0=0.71909,p_1=0.17451, p_2=0.10640<br>omega$_0$=0.20926, omega$_1$=1,<br>omega$_2$=2.54602
**M0, omega=1:fixed omega**	4	-12018.938	None

RPS16:

**Models**	**p**	**ℓ**	**Estimates of parameters**
**M0:one ratio**	1	-14320.835	omega=0.286
**M1a:nearly neutral**	2	-14134.842	p_0=0.76,p_1=0.24, omega$_0$=0.226, omega$_1$=1
**M2a:positive selection**	4	-14646.878	p_0=0,p_1=0.17451, p_2=0.10640 omega$_0$=0, omega$_1$=1, omega$_2$=4.65
**M0, omega=1:fixed omega**	4	-15340.056	None

RPS18:

**Models**	**p**	**ℓ**	**Estimates of parameters**
**M0:one ratio**	1	-14320.835	omega=0.328
**M1a:nearly neutral**	2	-14134.842	p_0=0.0001,p_1=0.9999, omega$_0$=0, omega$_1$=1.0000
**M2a:positive selection**	4	-14646.878	p_0=0.8292,p_1=0.11455, p_2=0.05563 omega$_0$=0.288, omega$_1$=1, omega$_2$=2.19489
**M0, omega=1:fixed omega**	4	-176000.284	None
