# Supplementary figures and images for: Tempo and Mode of Gene Duplication in Mammalian Ribosomal Protein Evolution
Source: PLoS One. 2014 Nov 4;9(11):e111721. doi: 10.1371/journal.pone.0111721 (PMC4219774; doi:10.1371/journal.pone.0111721)

APPENDIX 1 – 74 RP gene trees with all annotated duplication events. For legend, see figure 3.

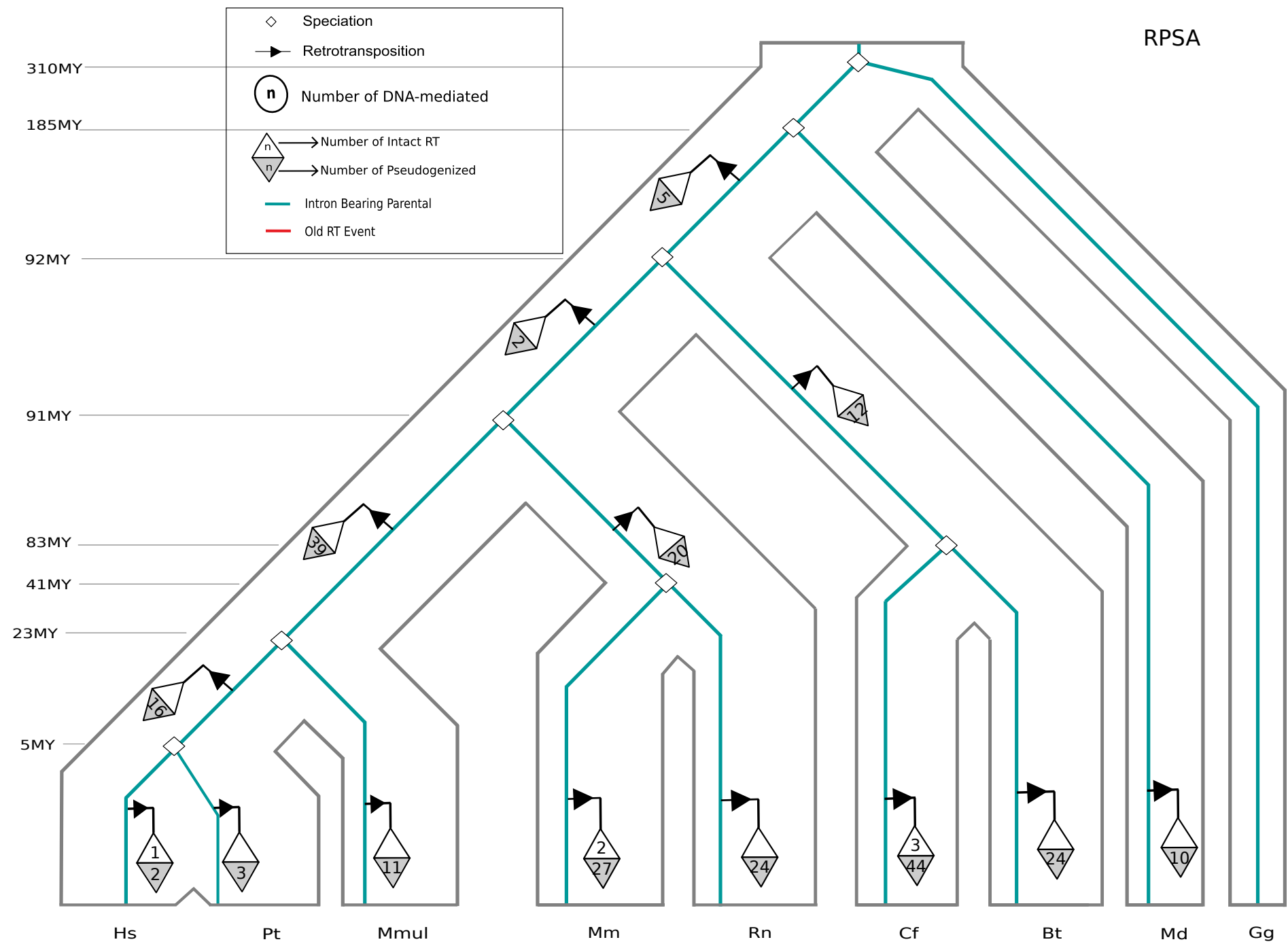

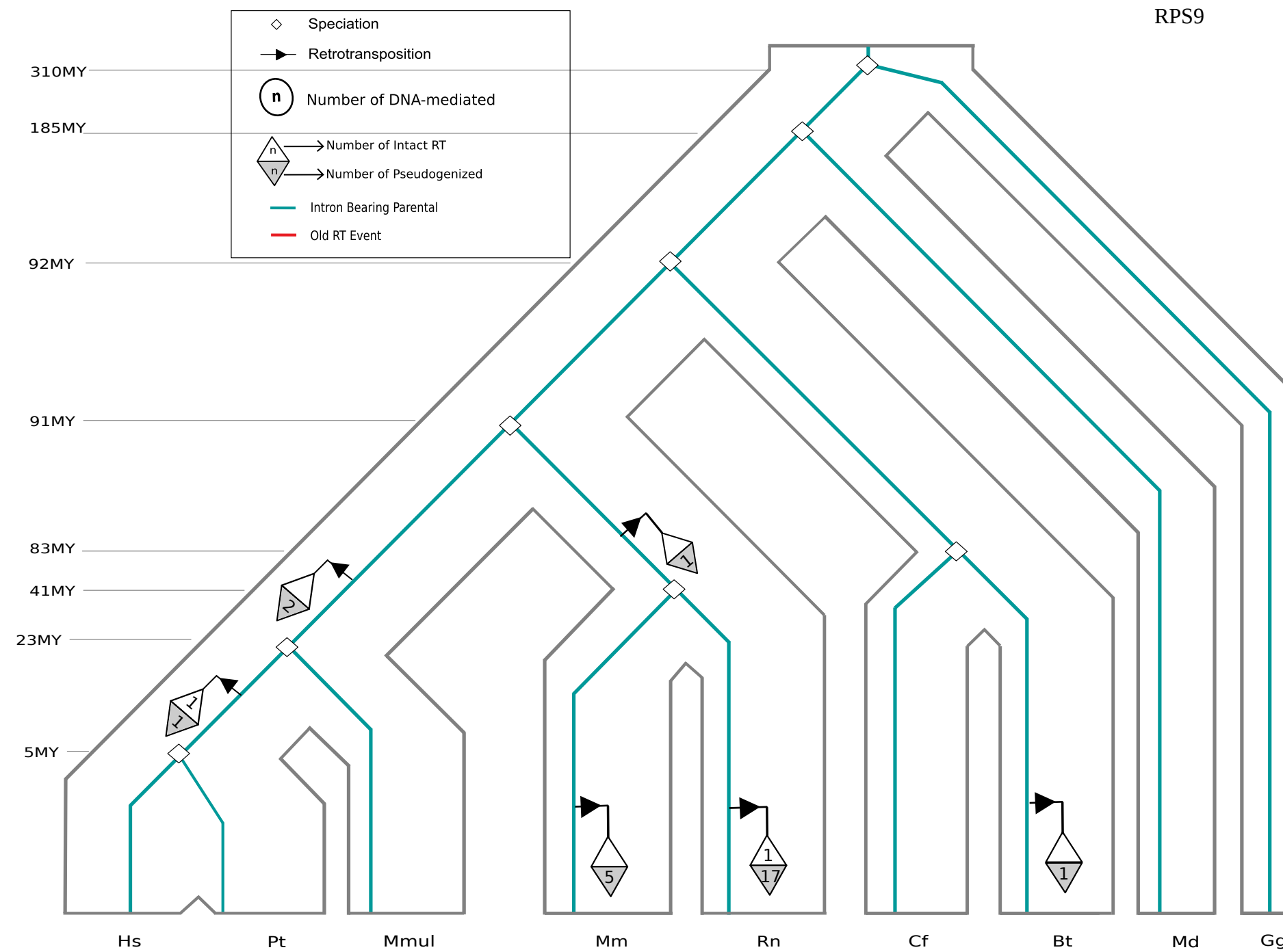

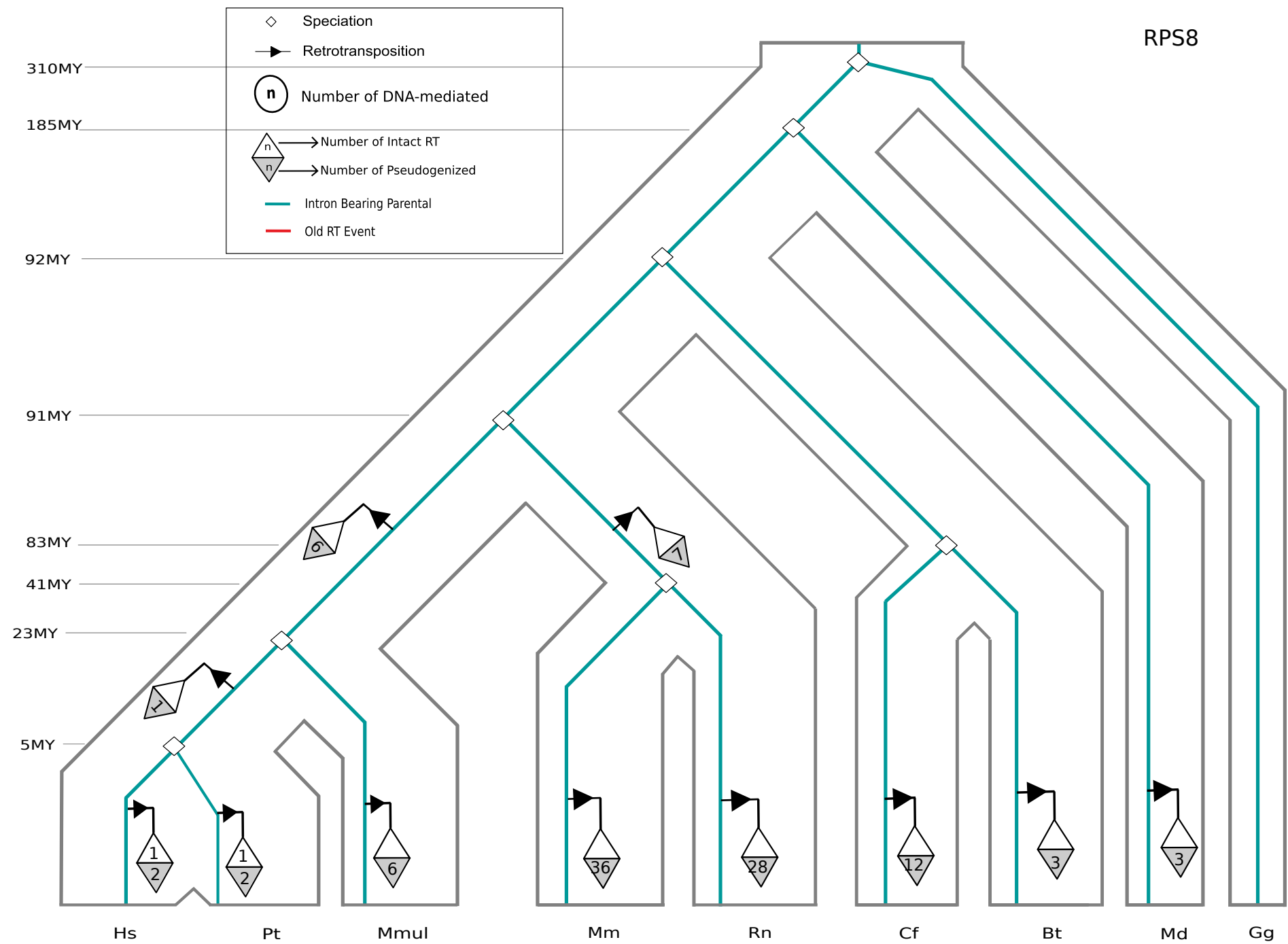

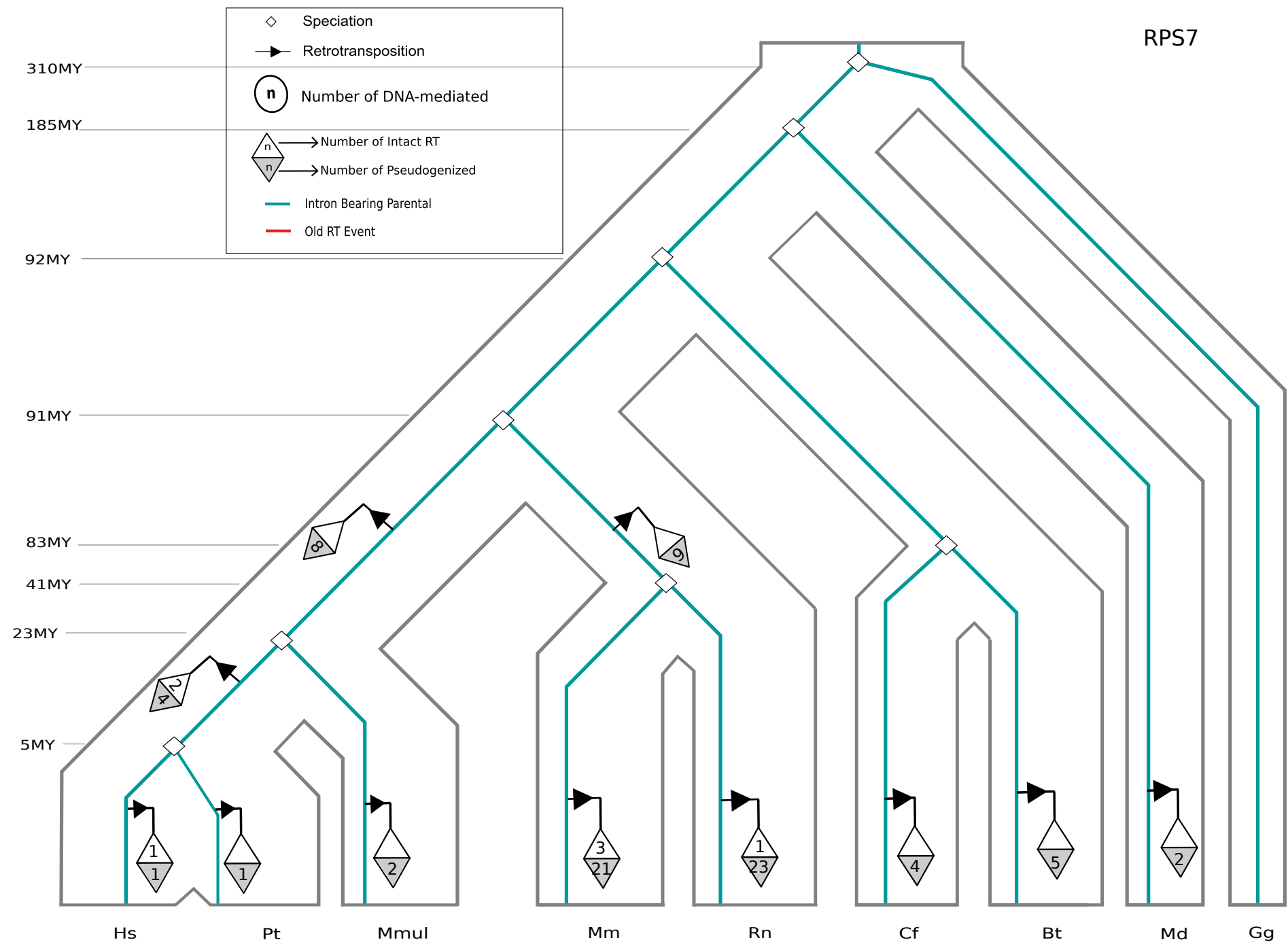

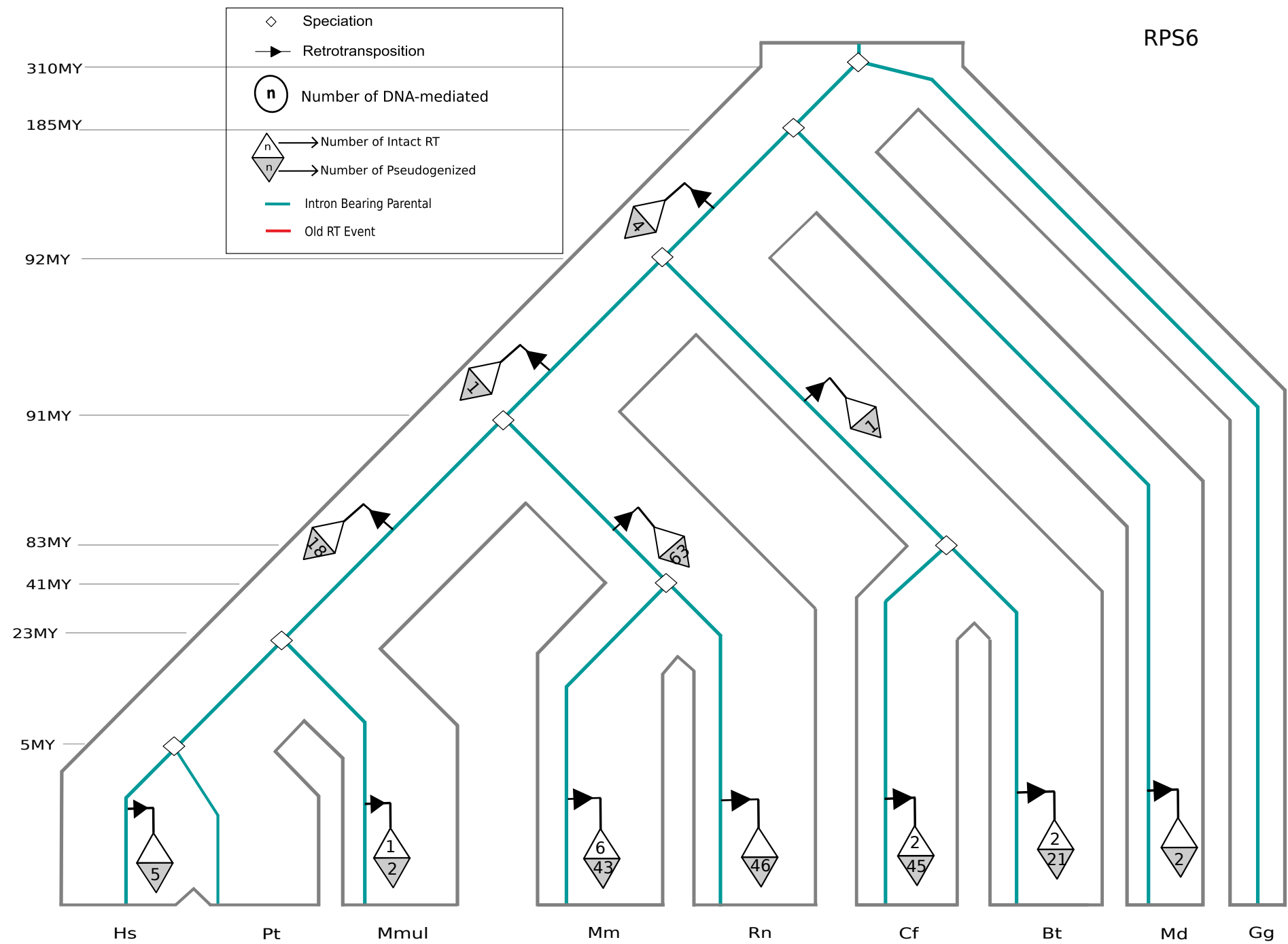

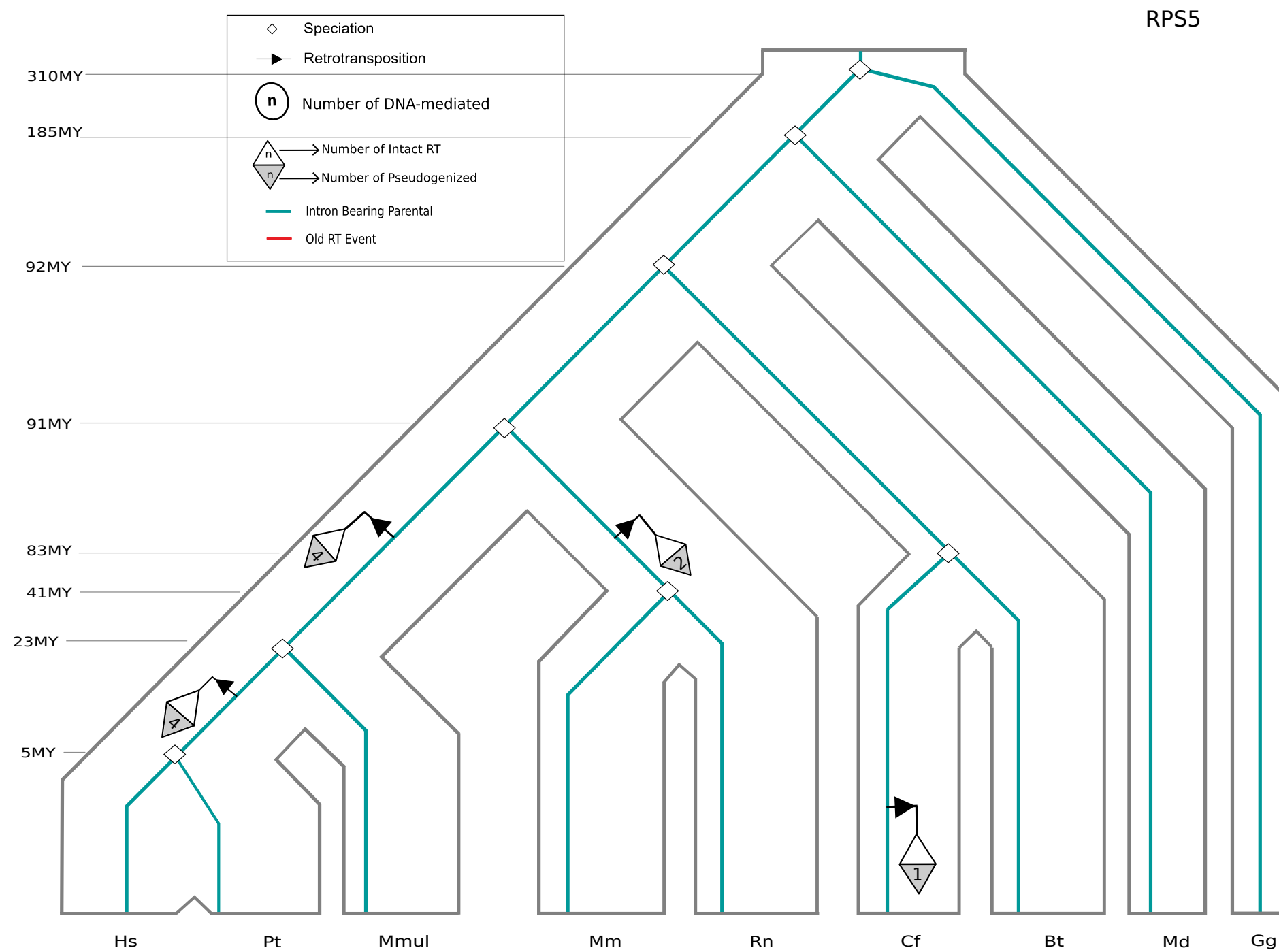

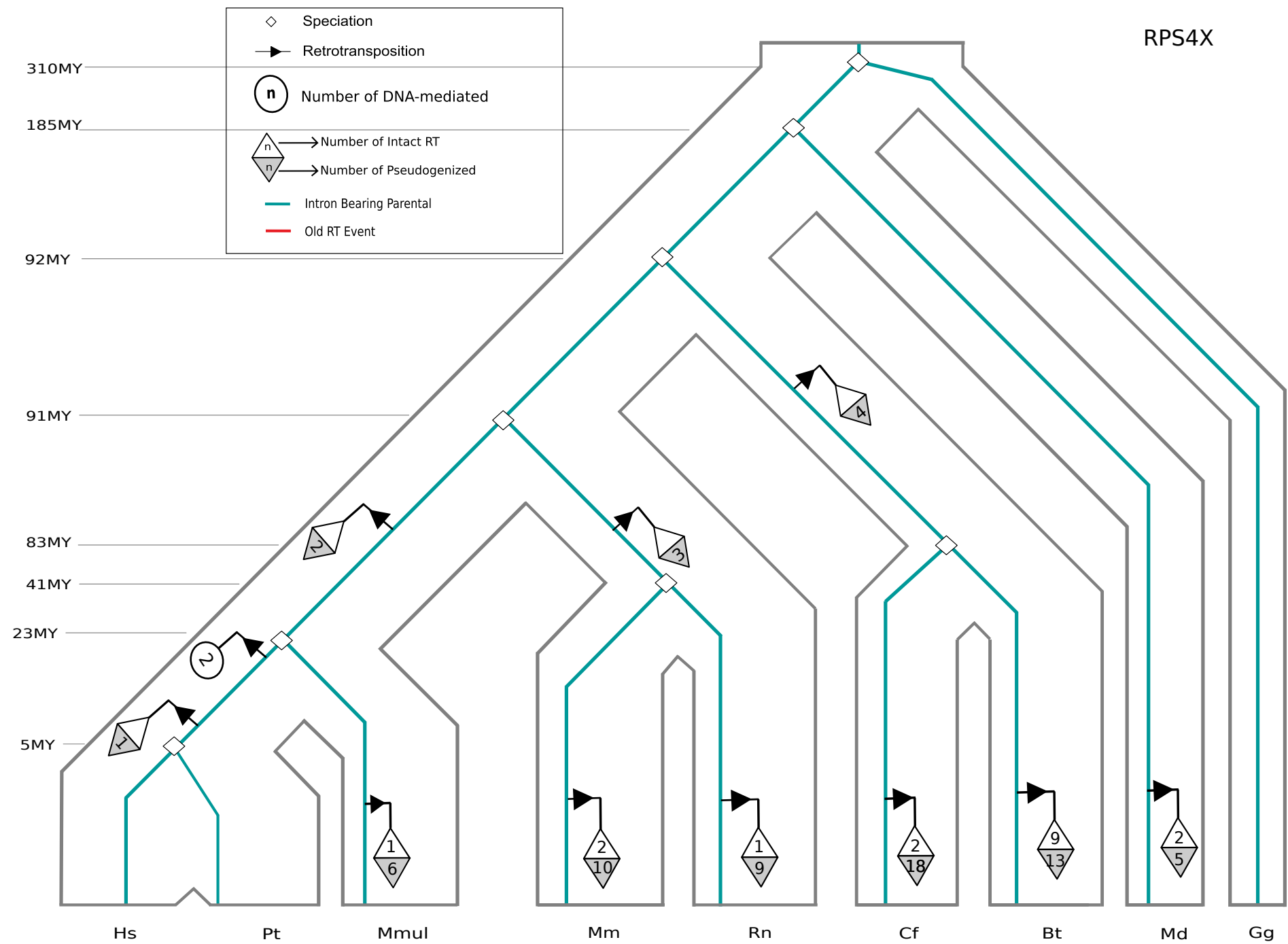

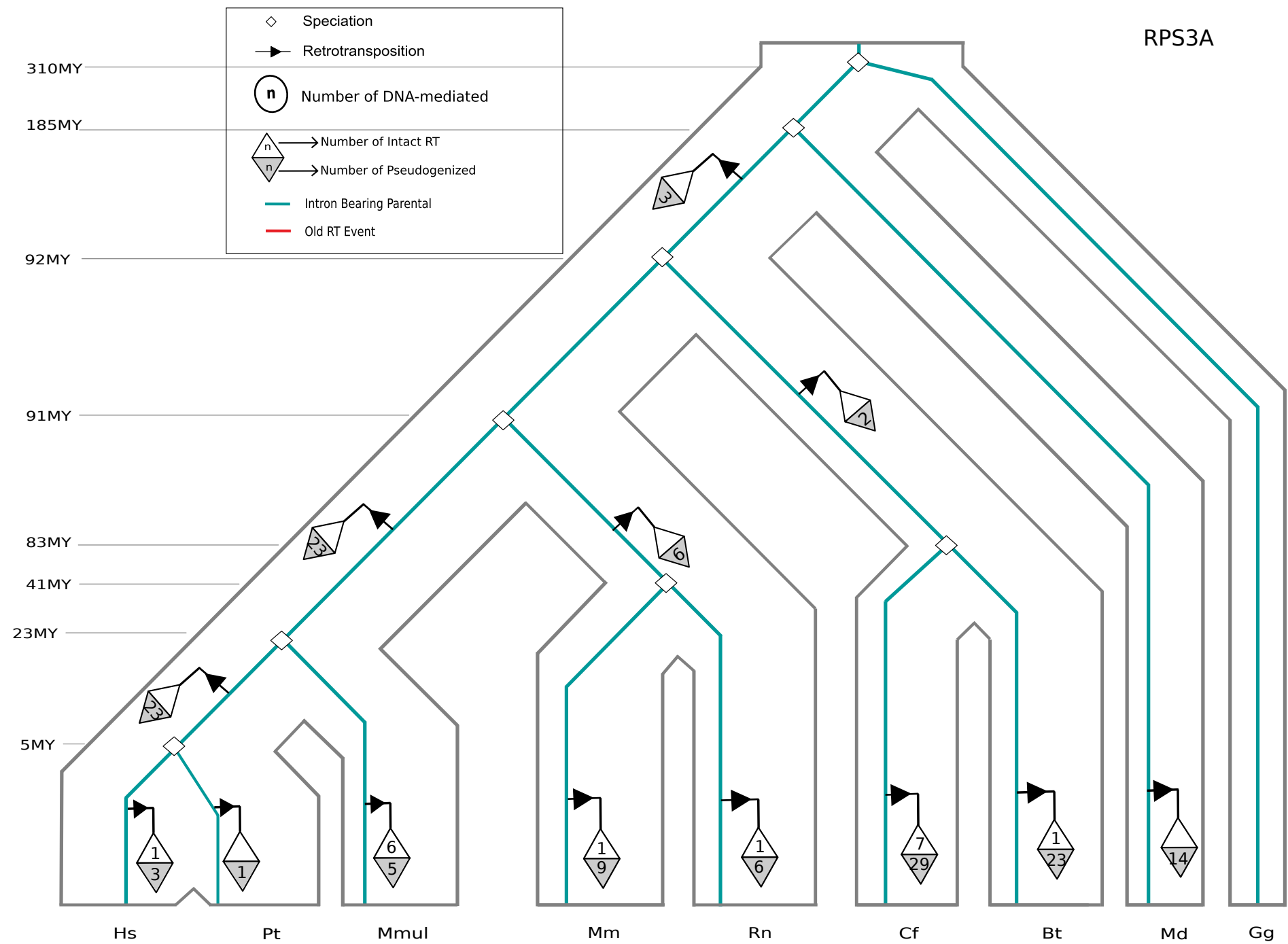

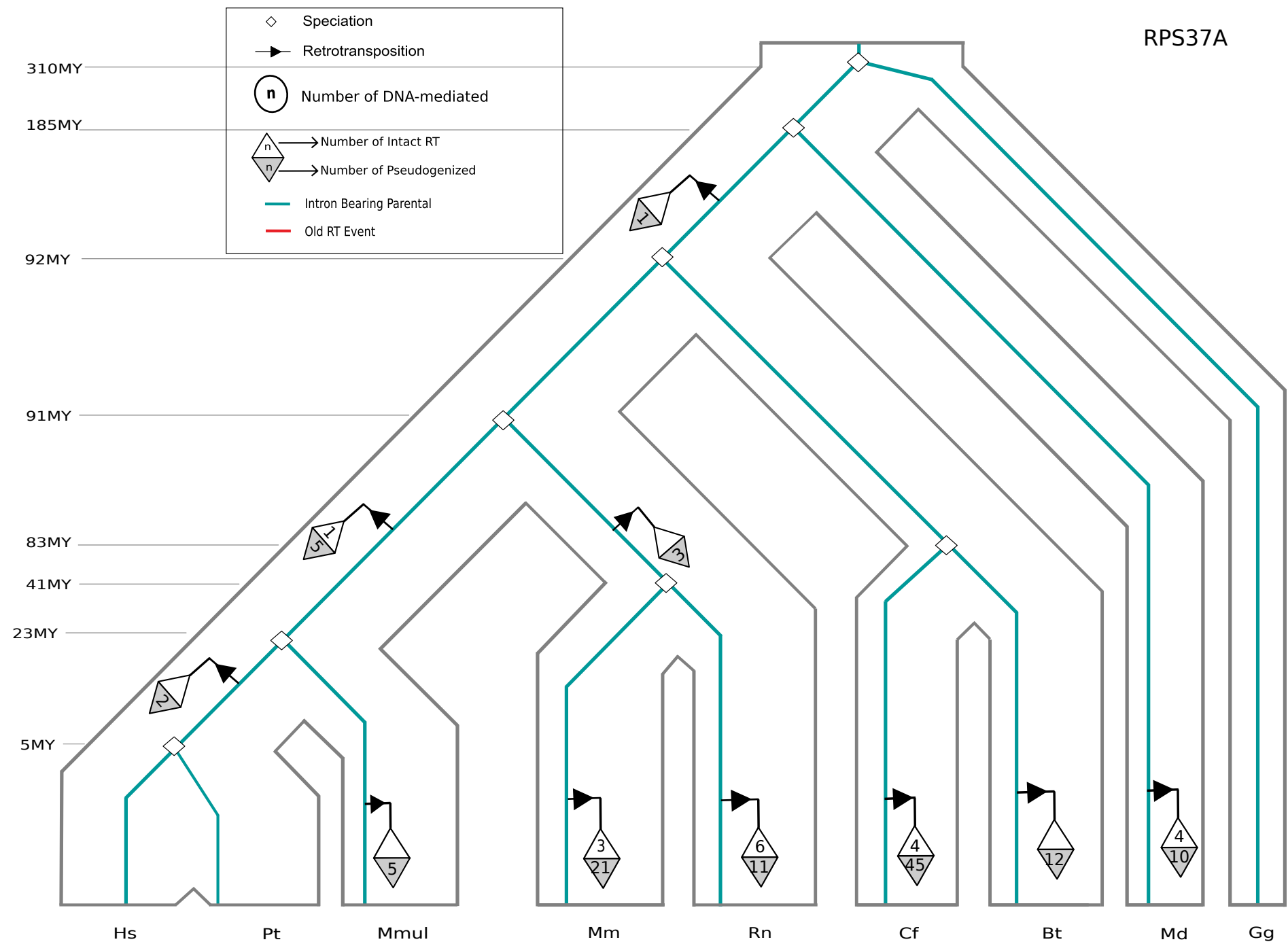

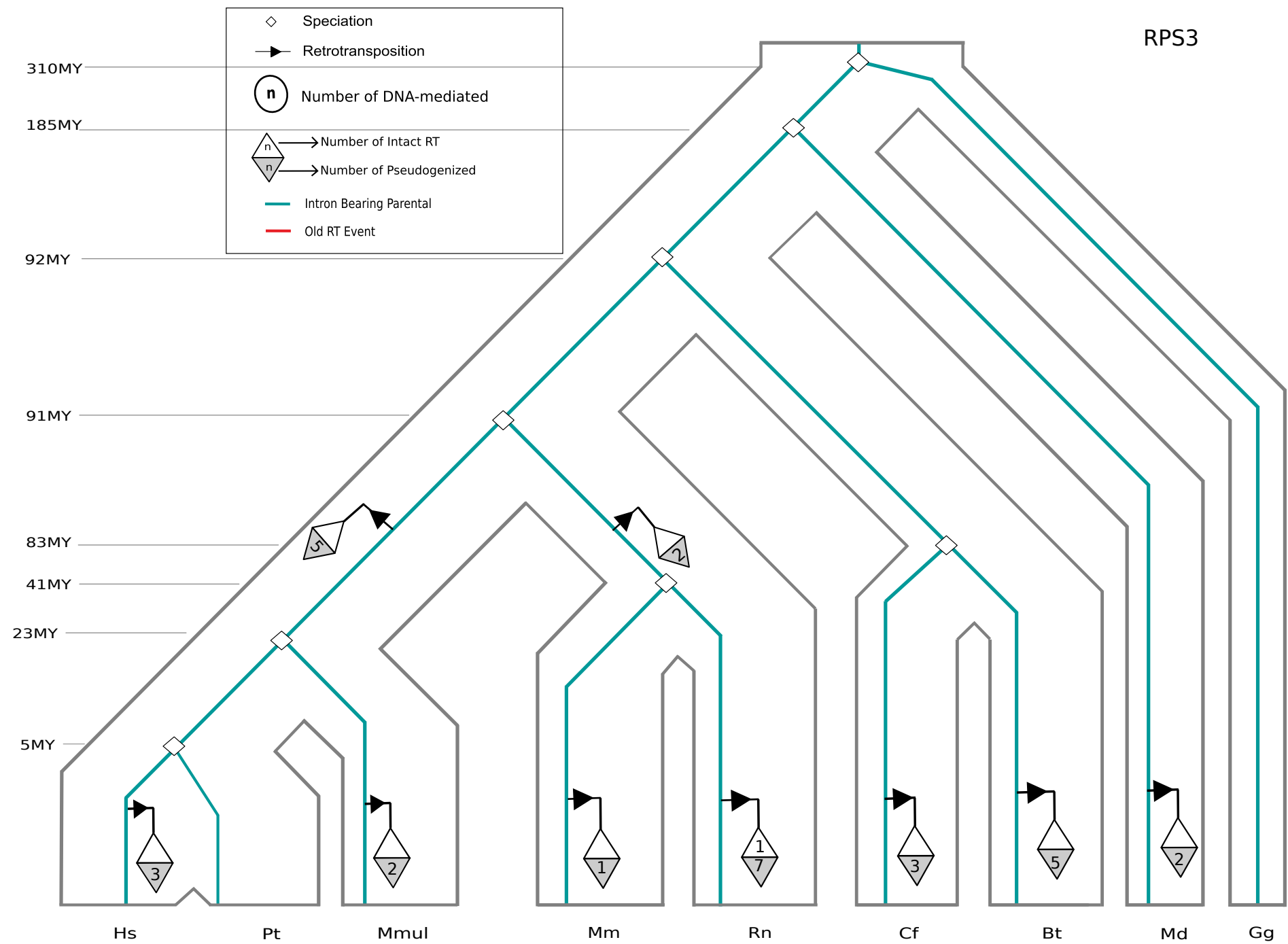

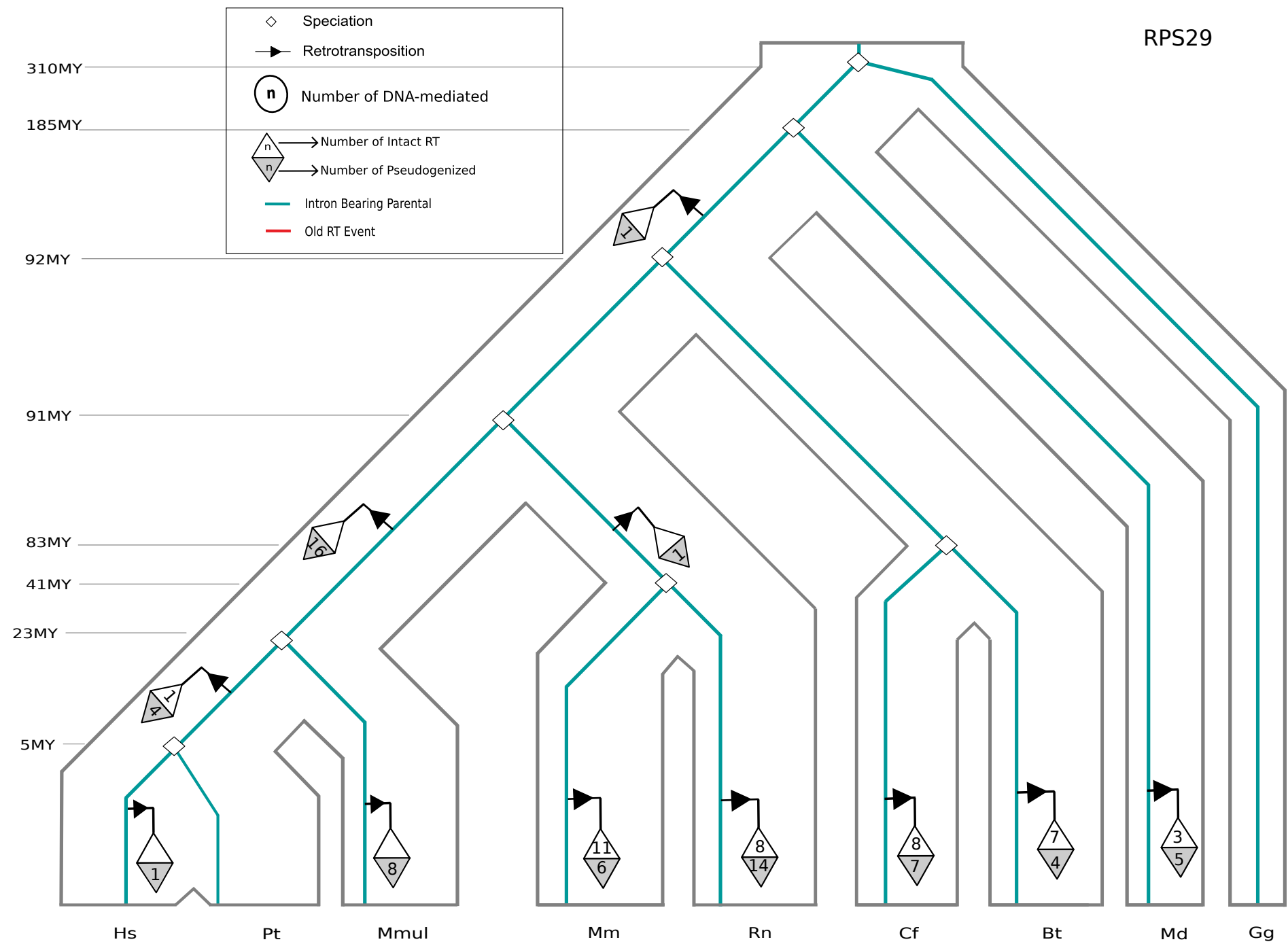

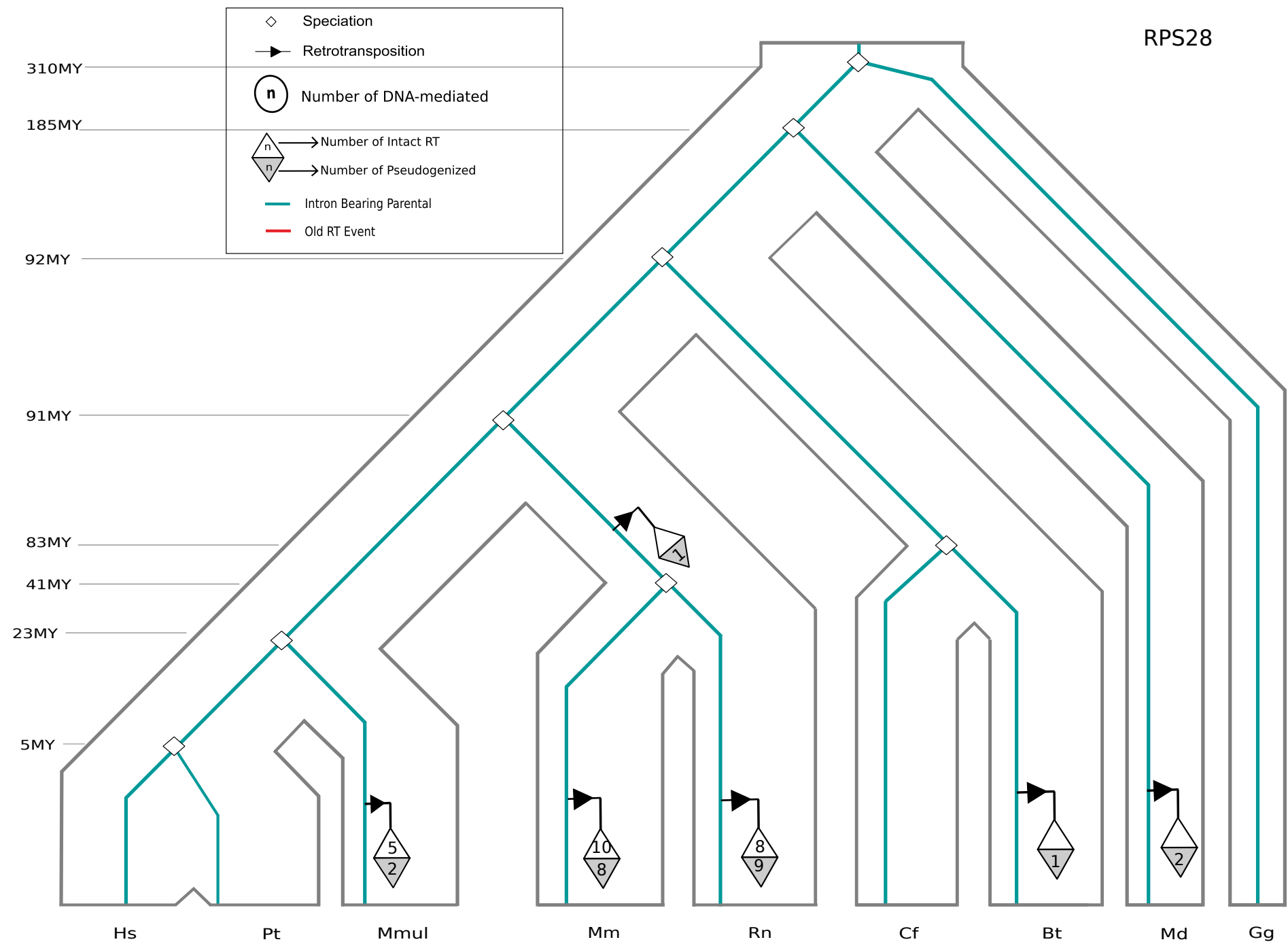

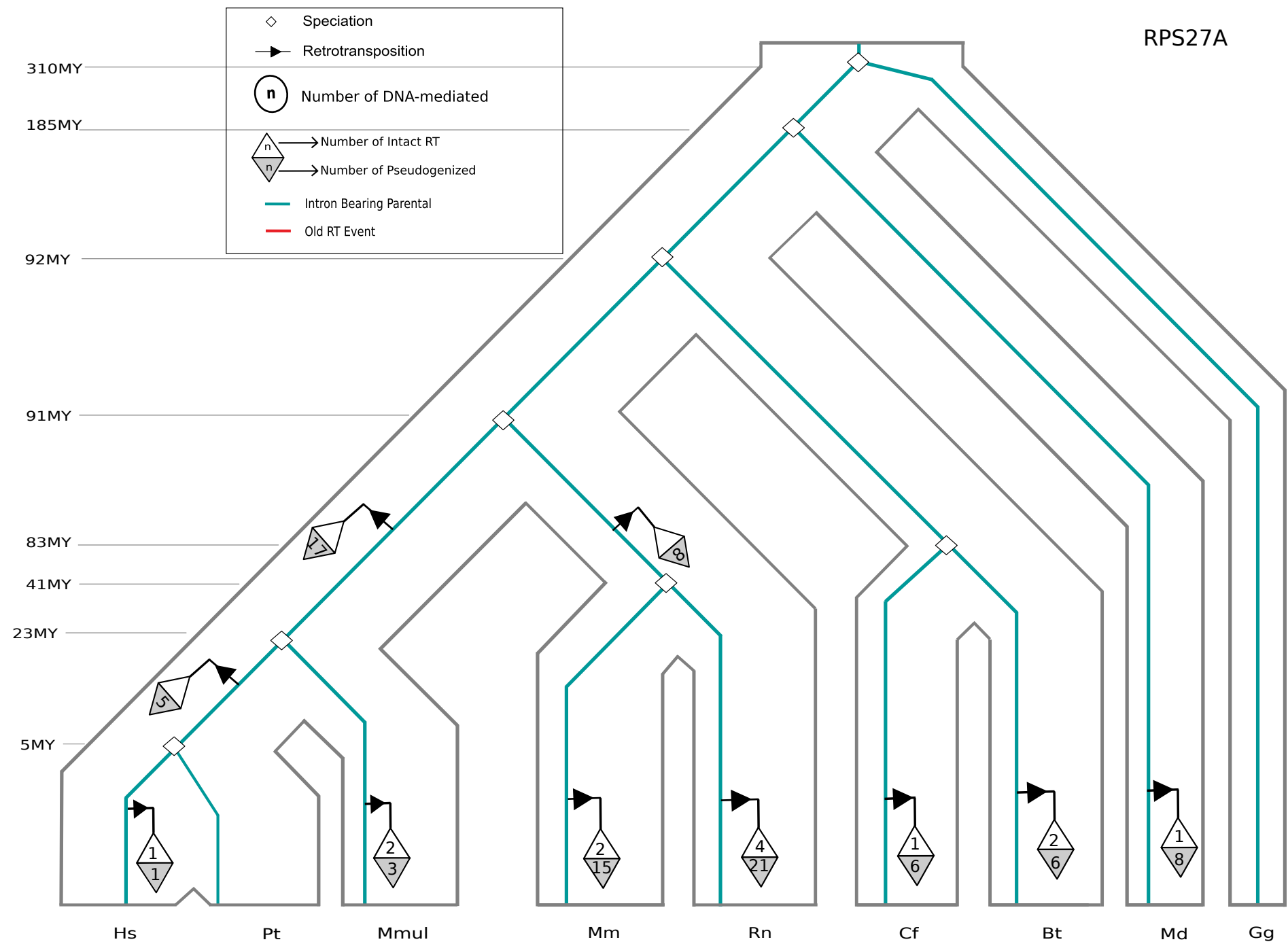

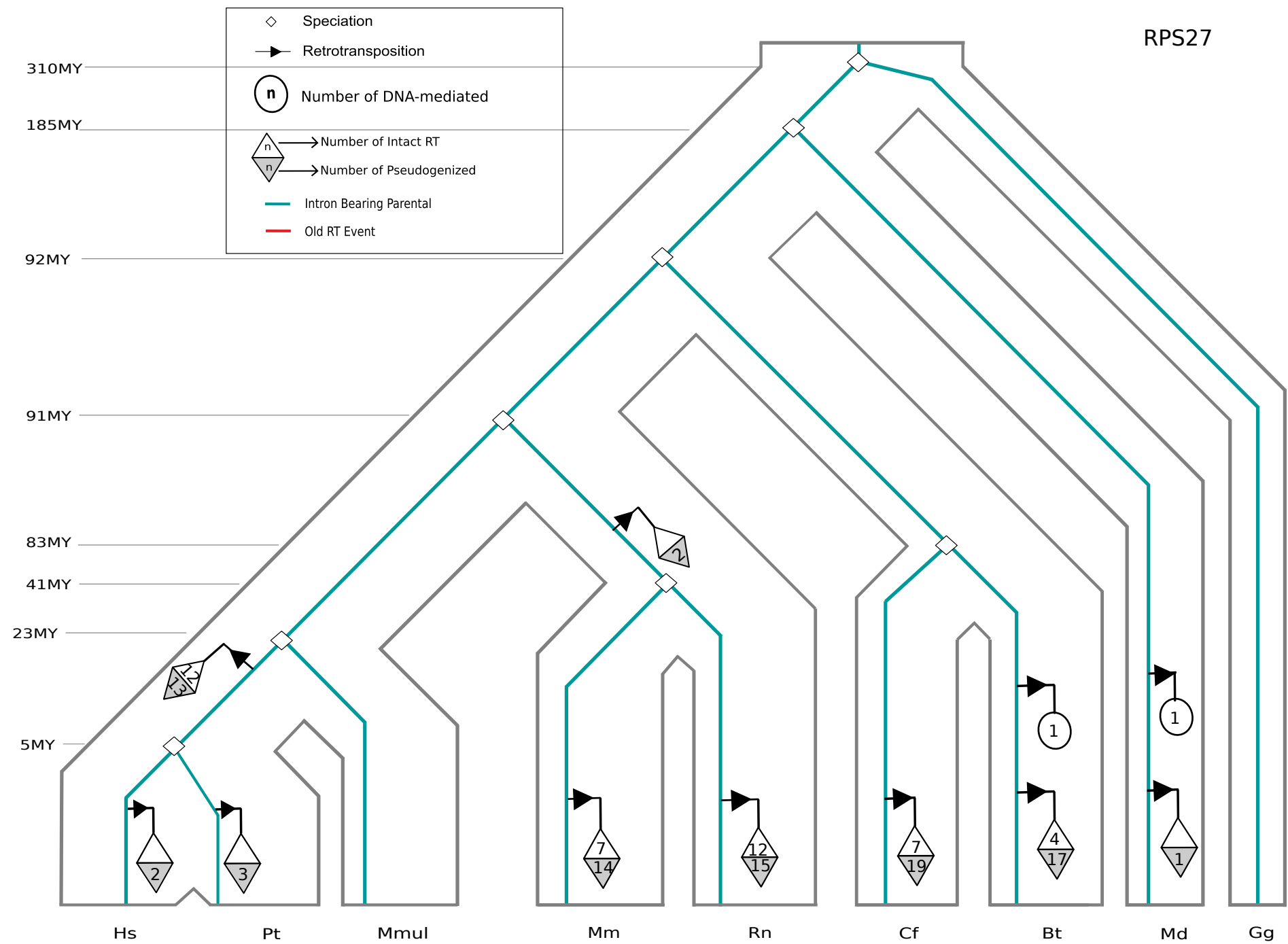

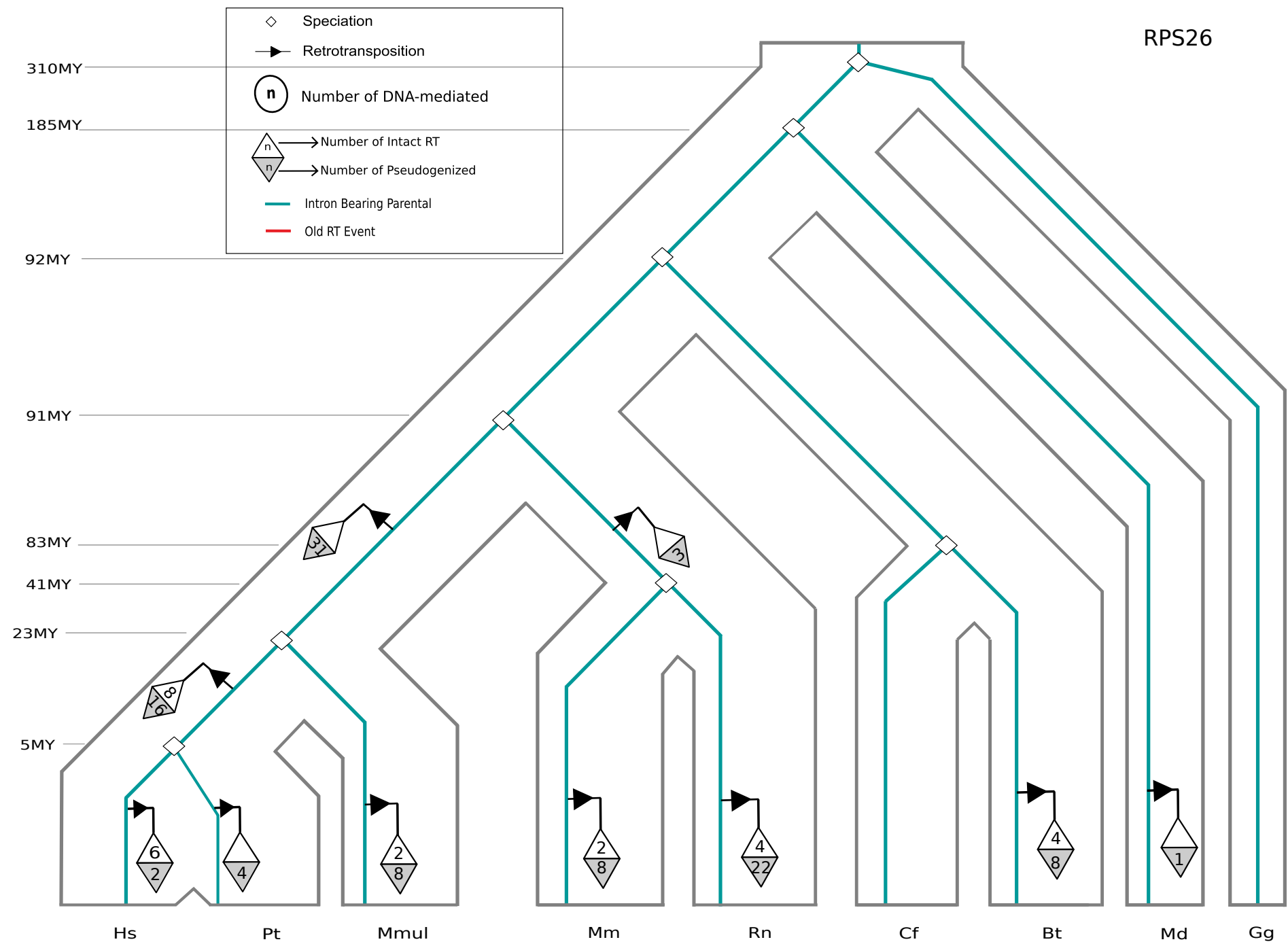

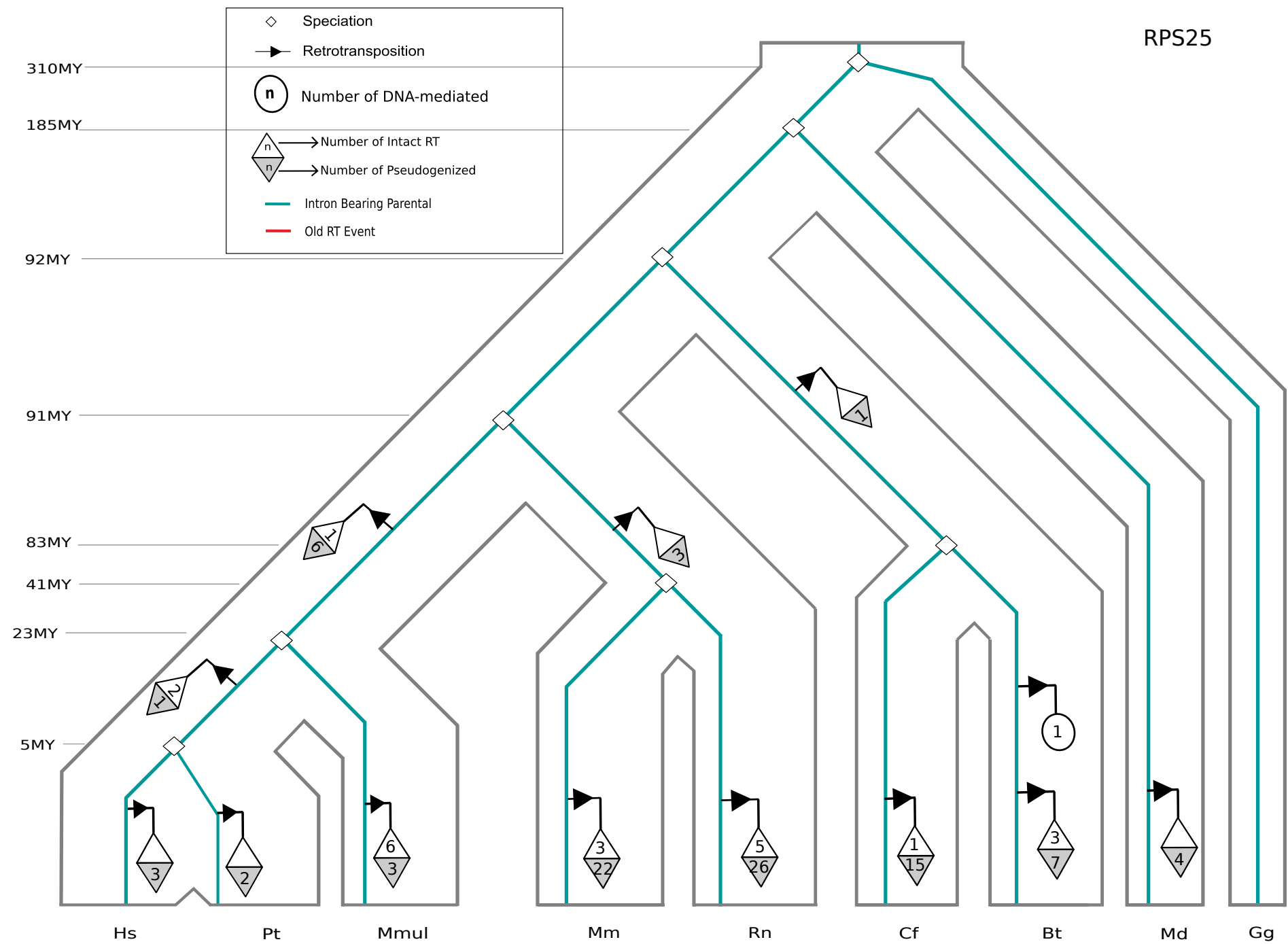

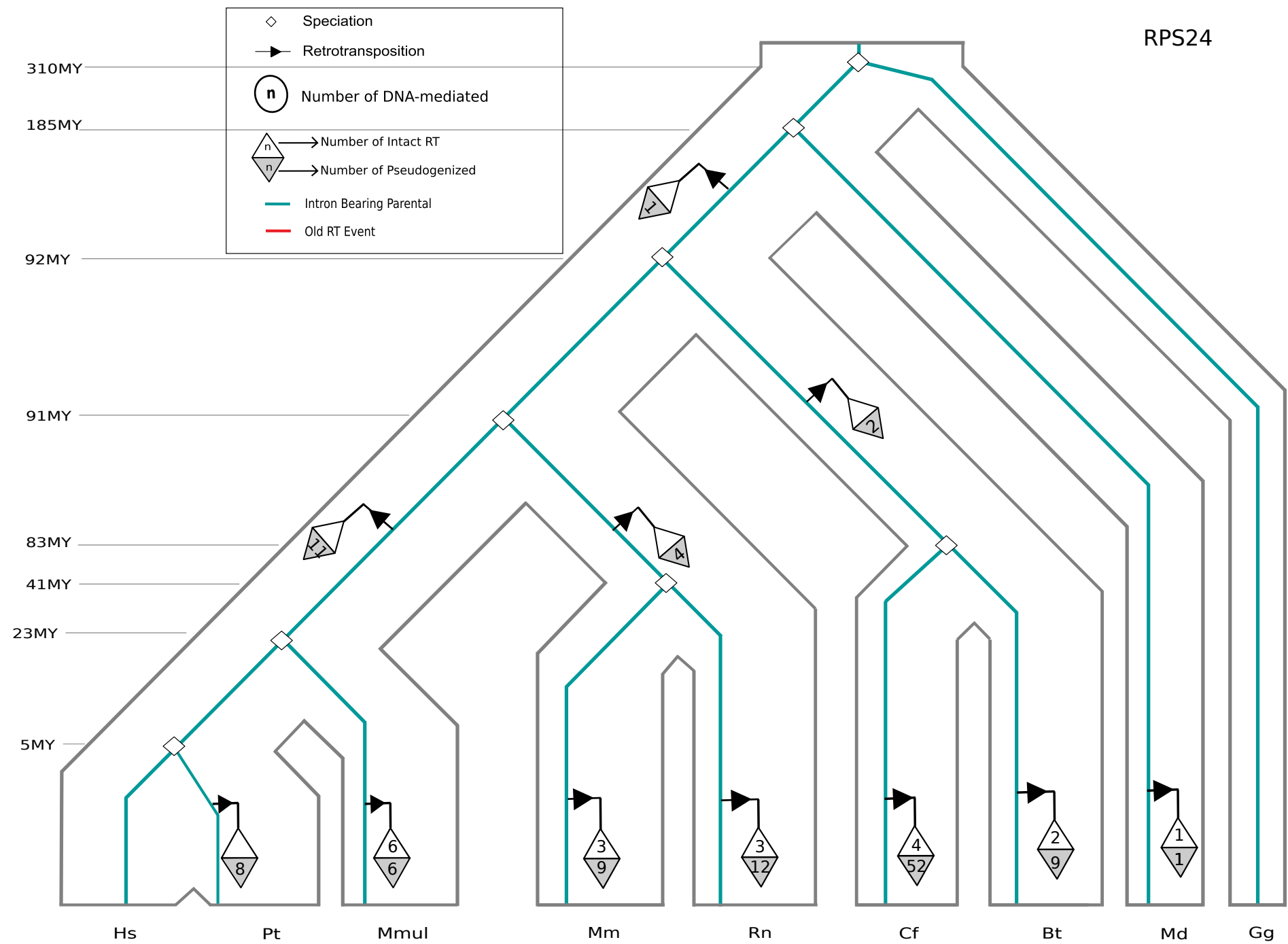

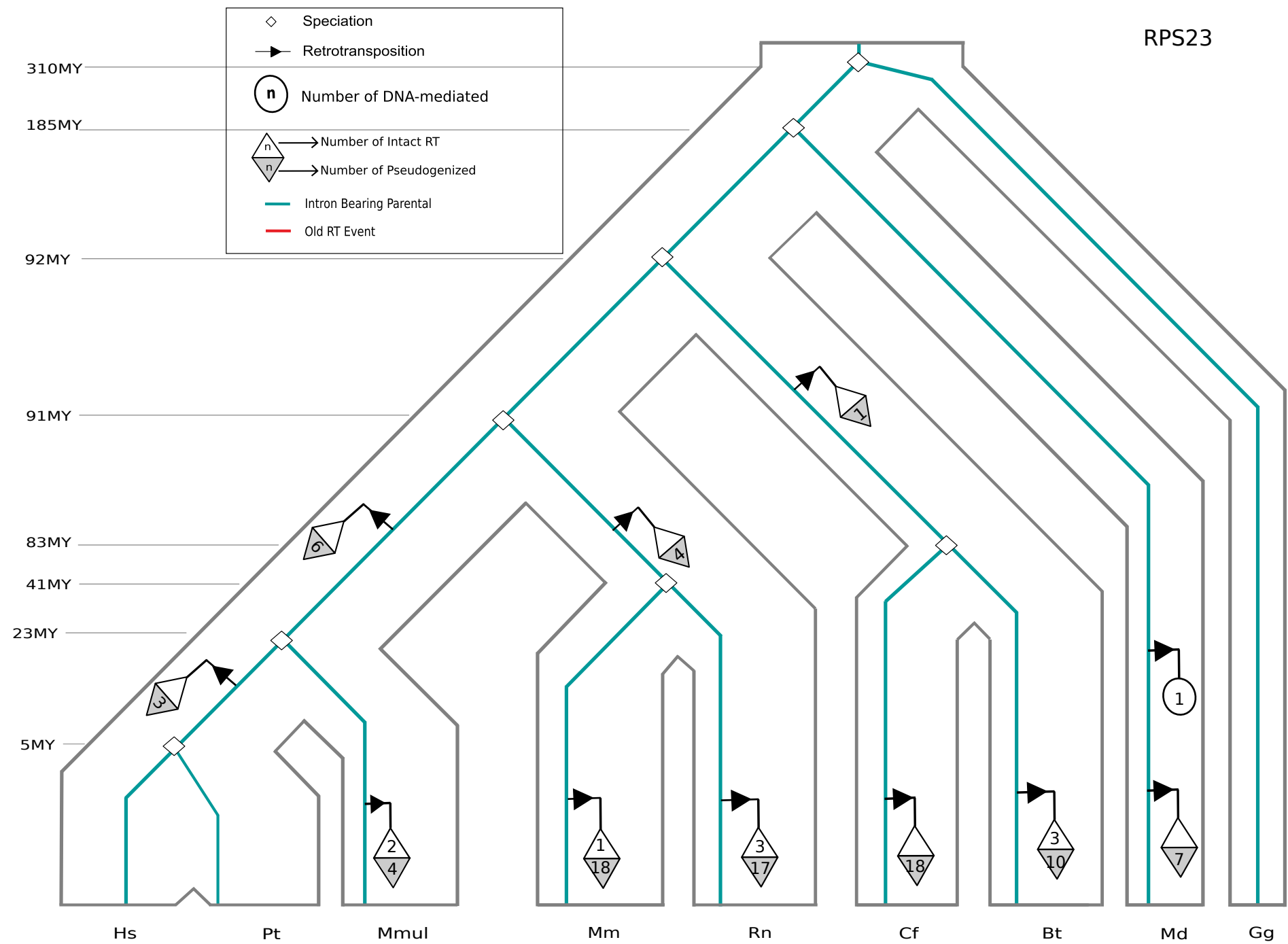

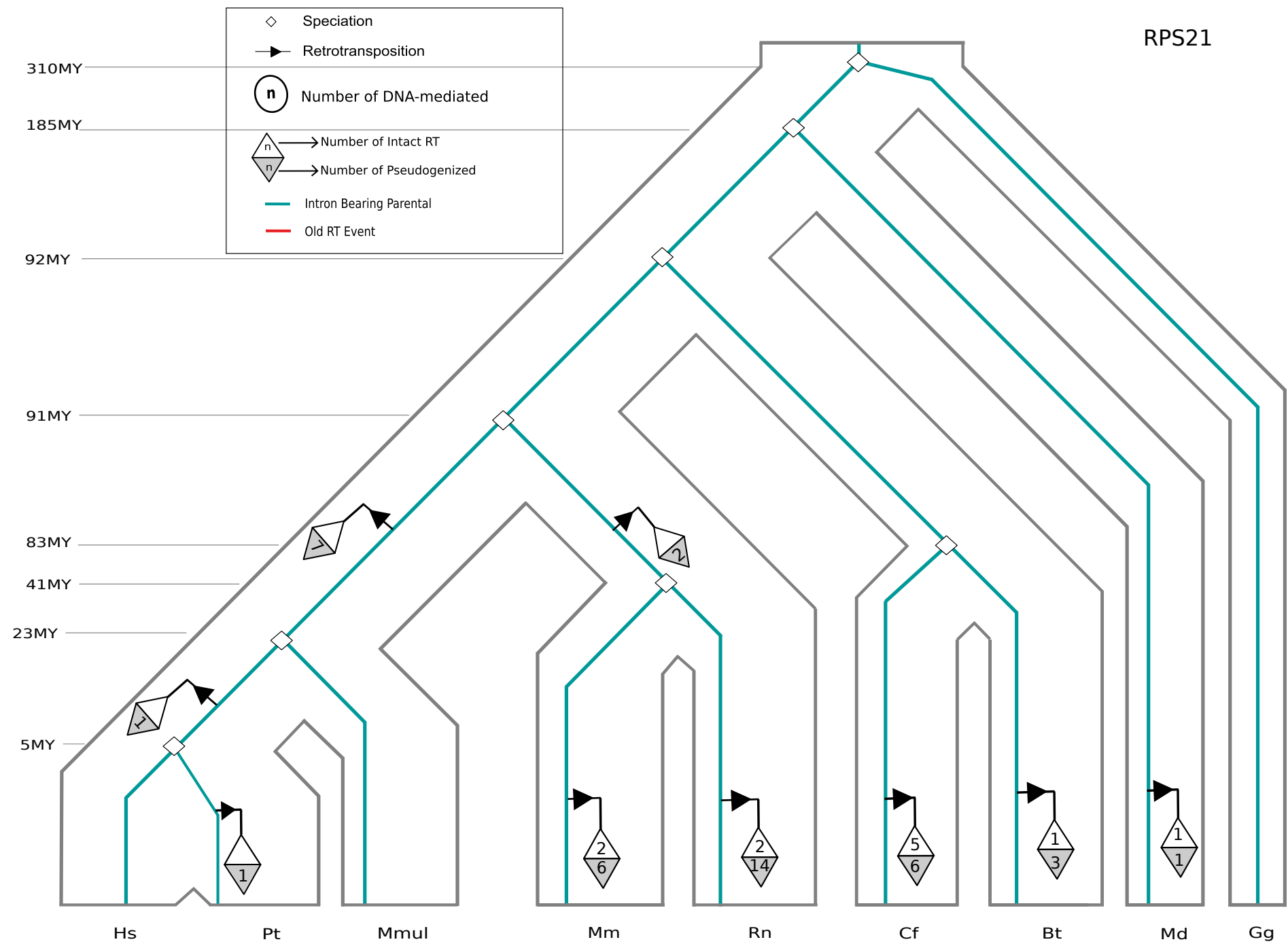

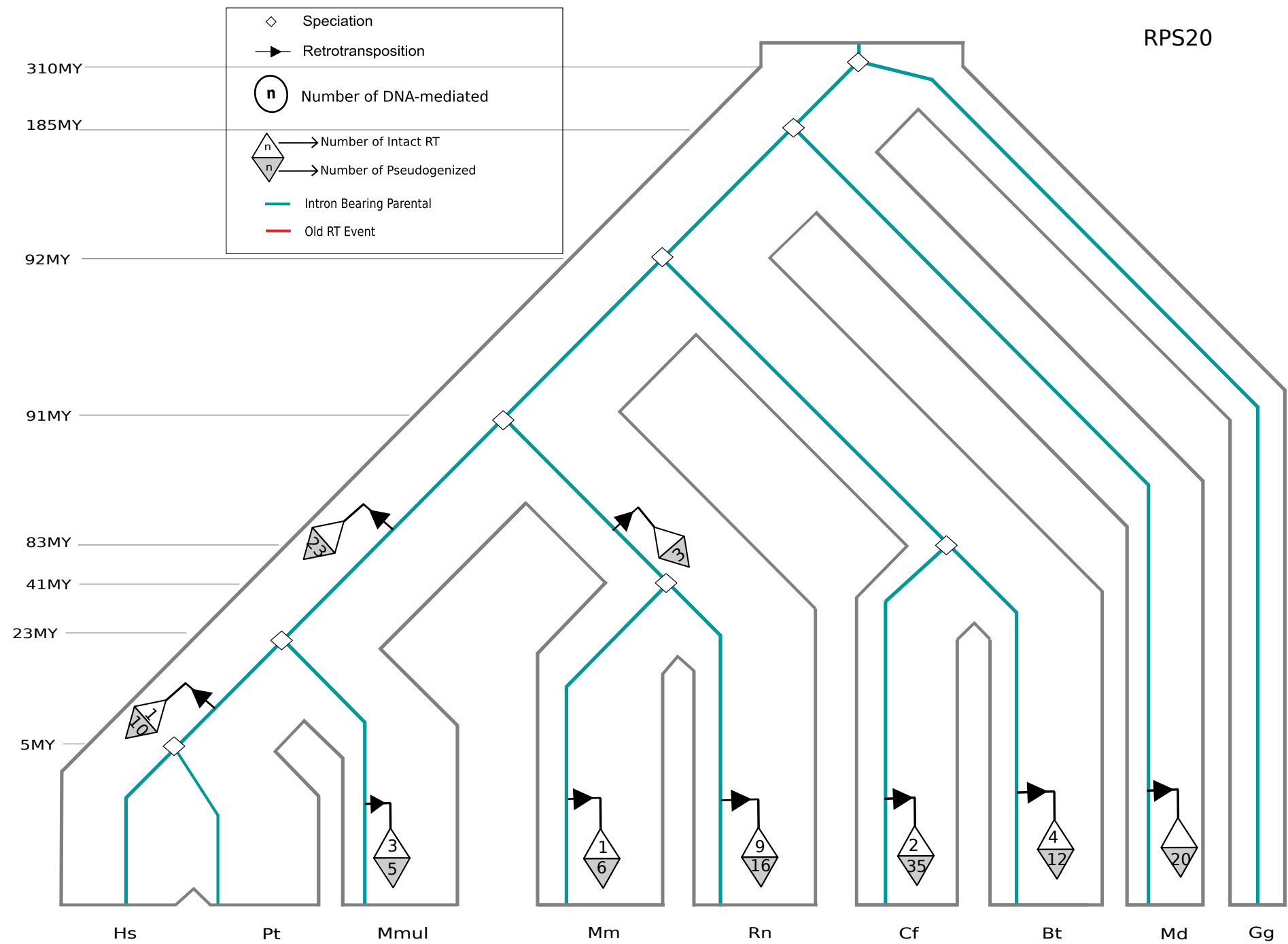

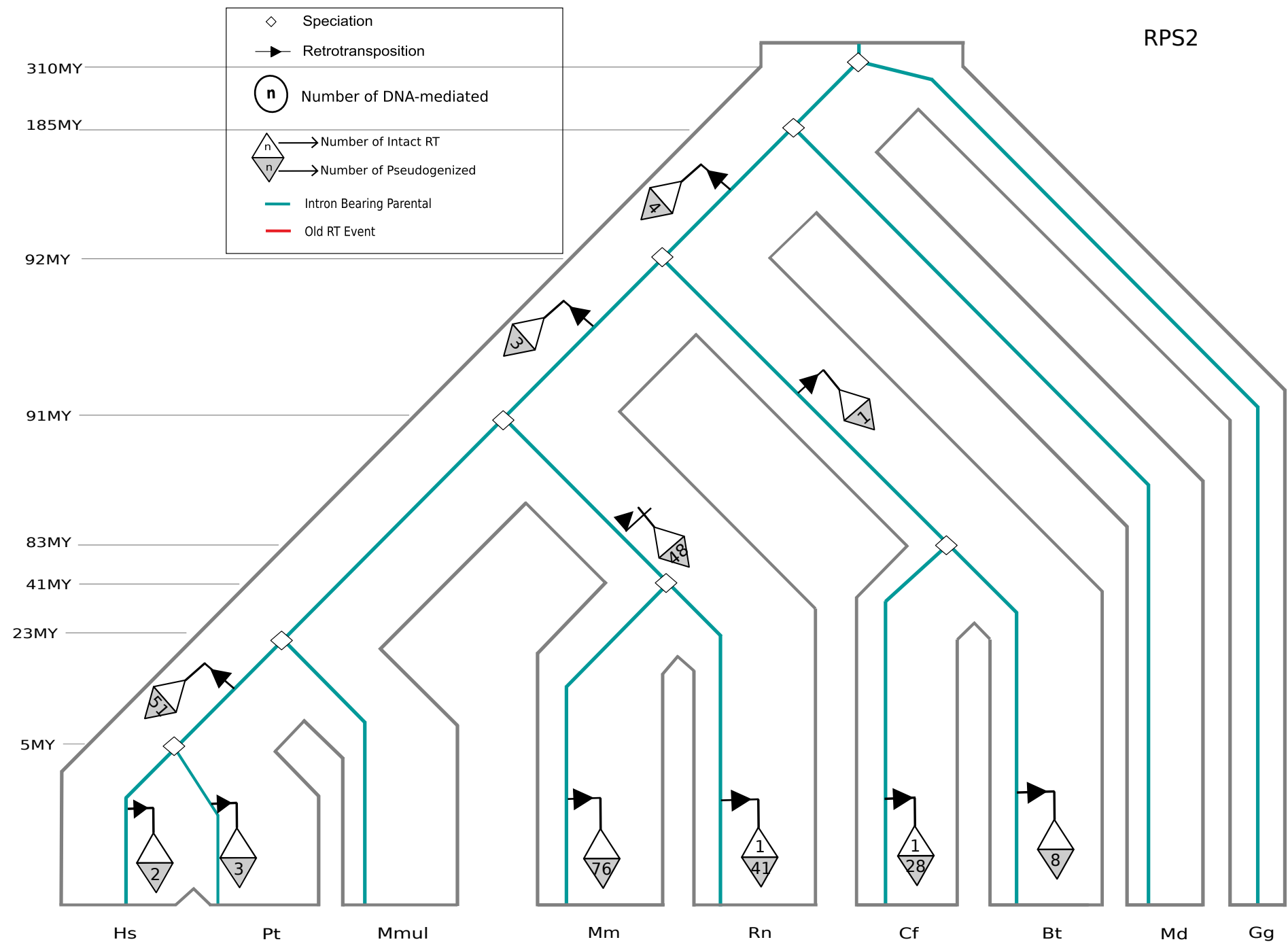

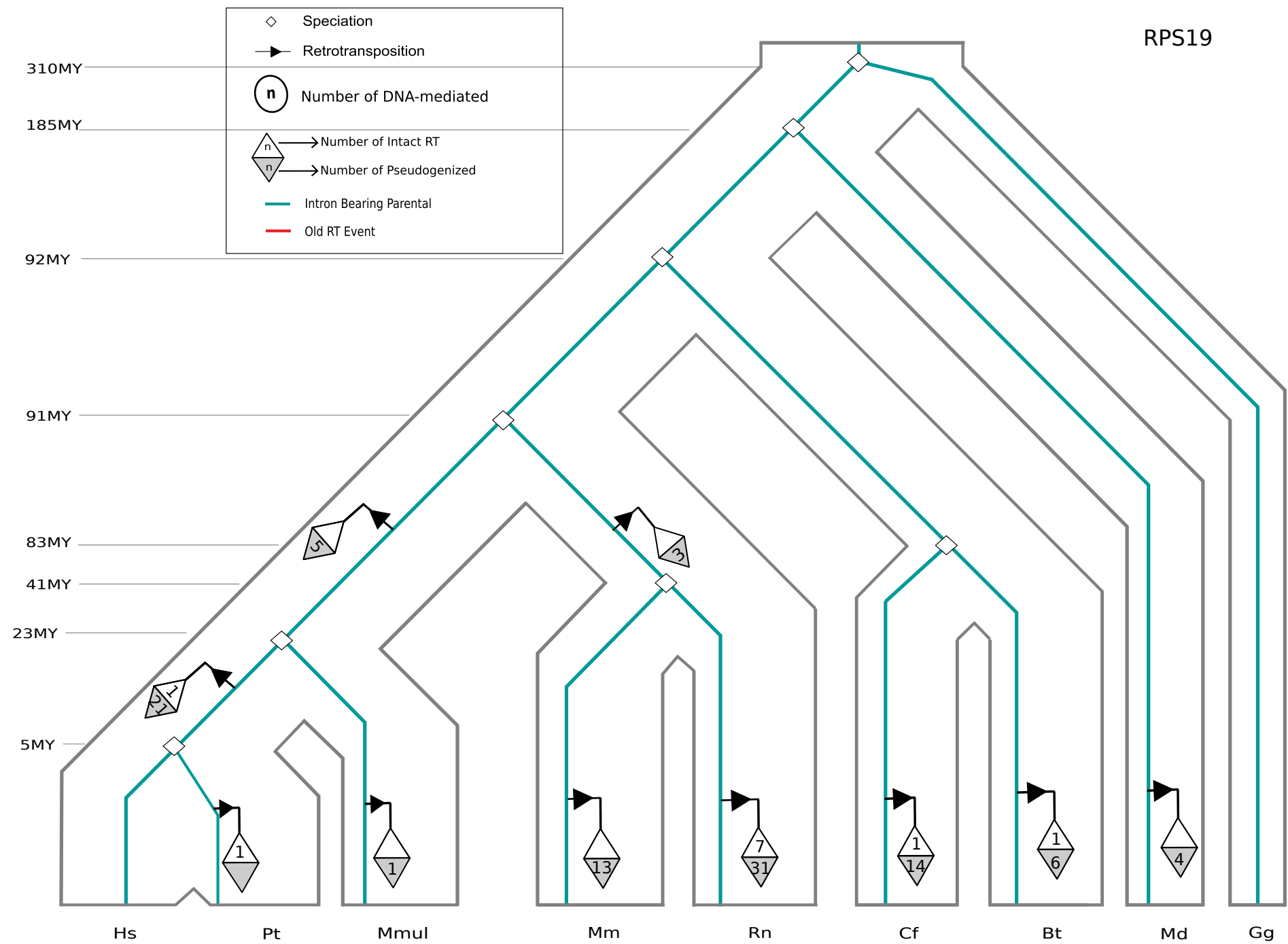

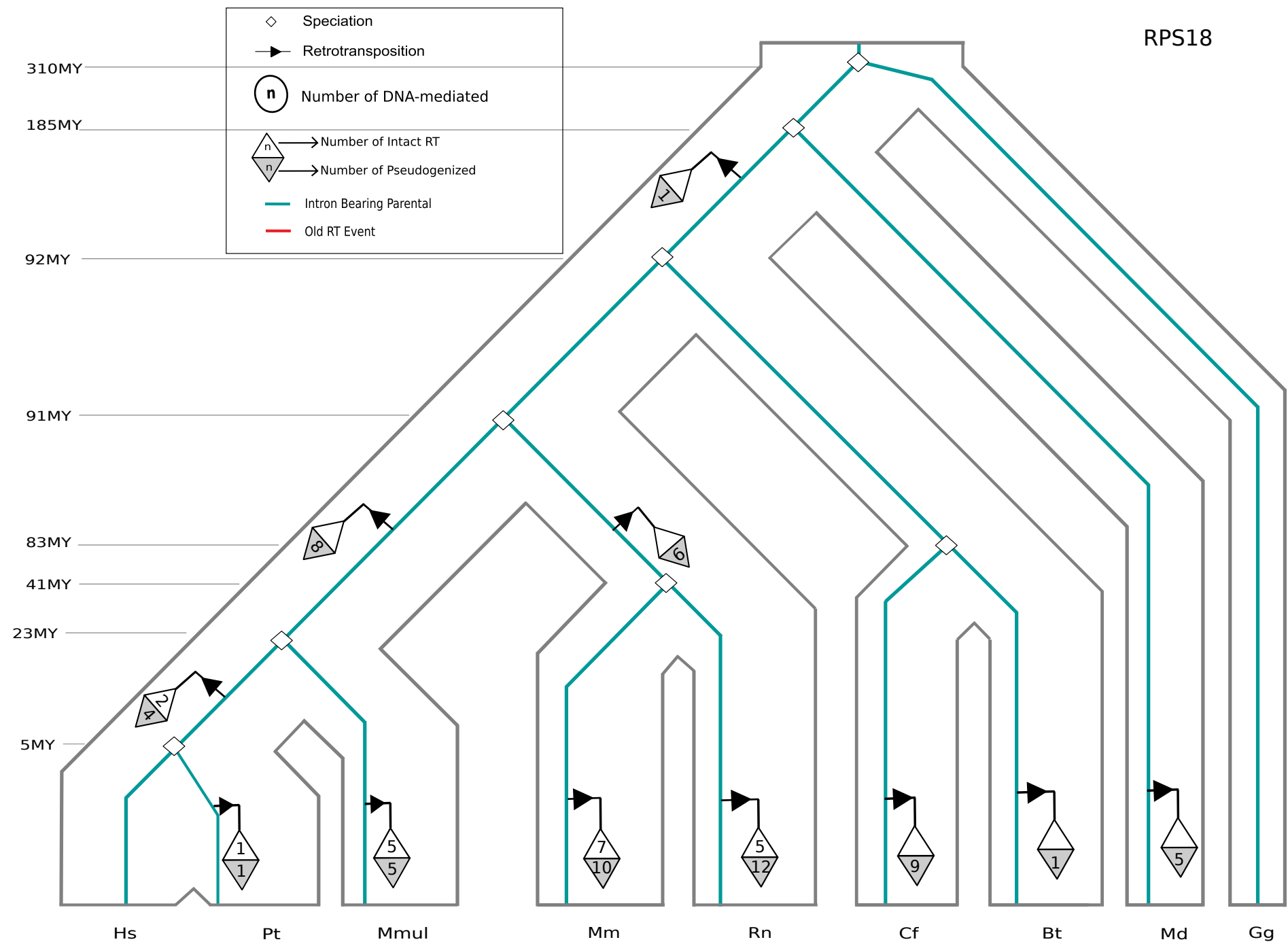

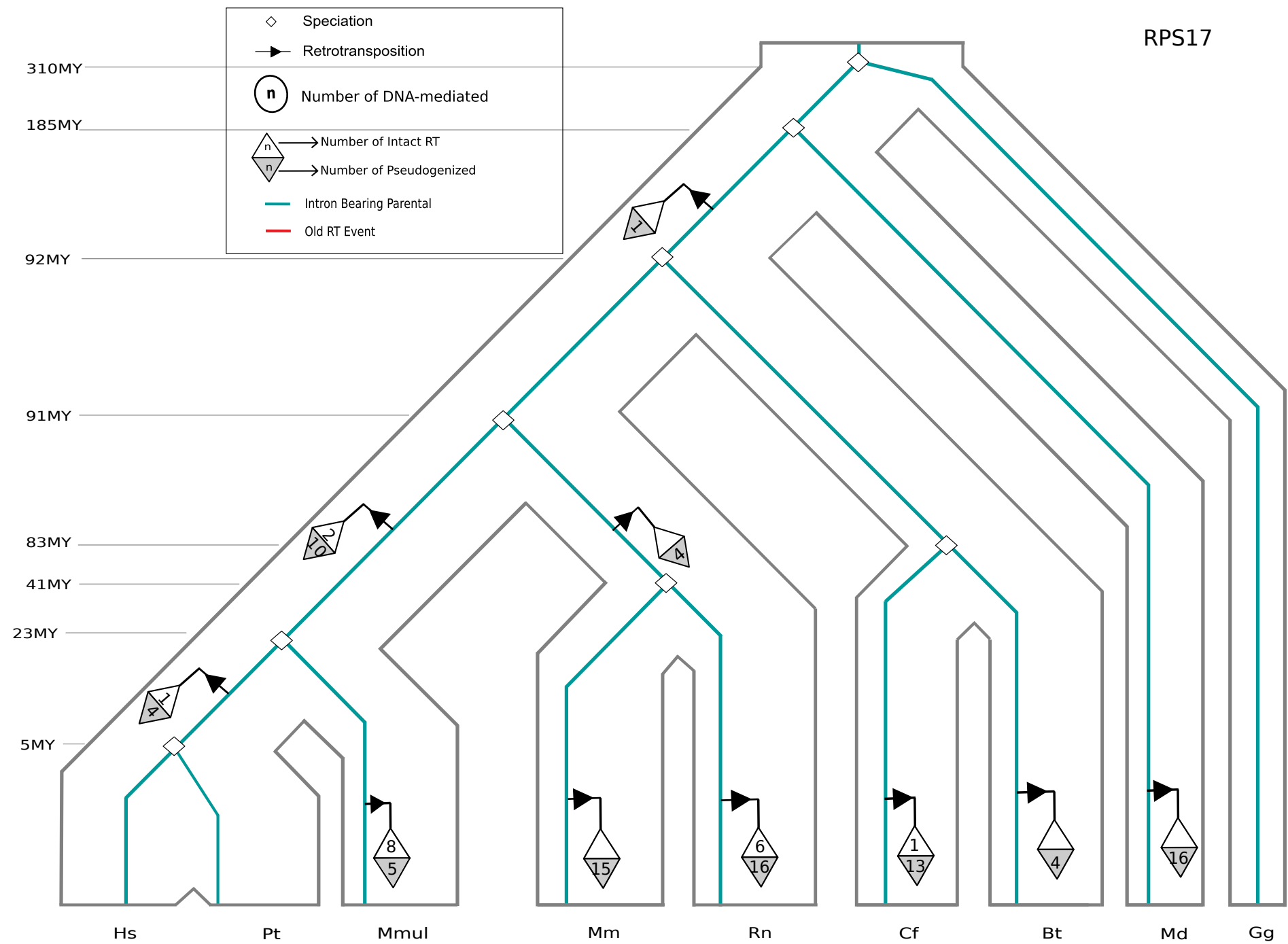

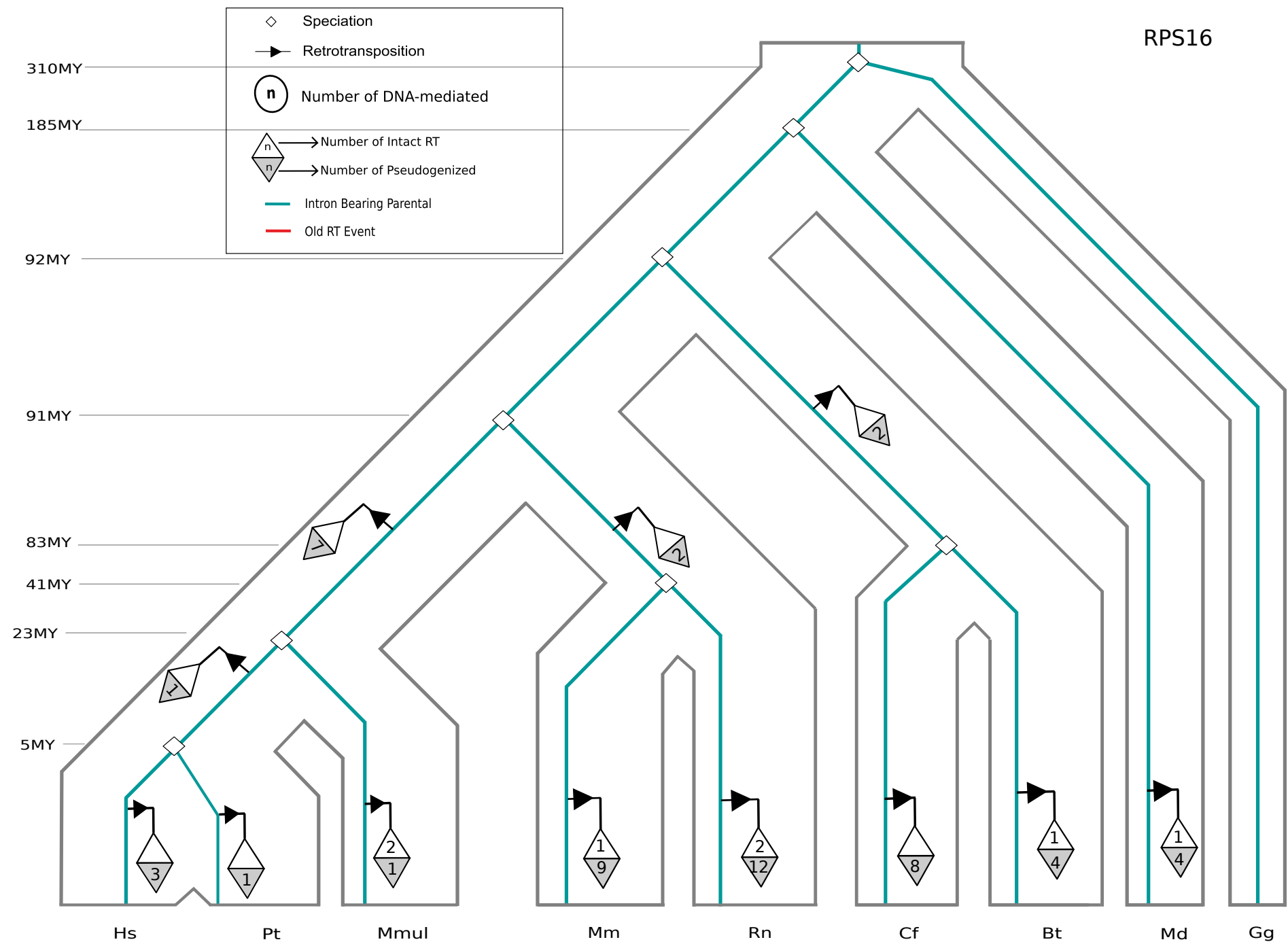

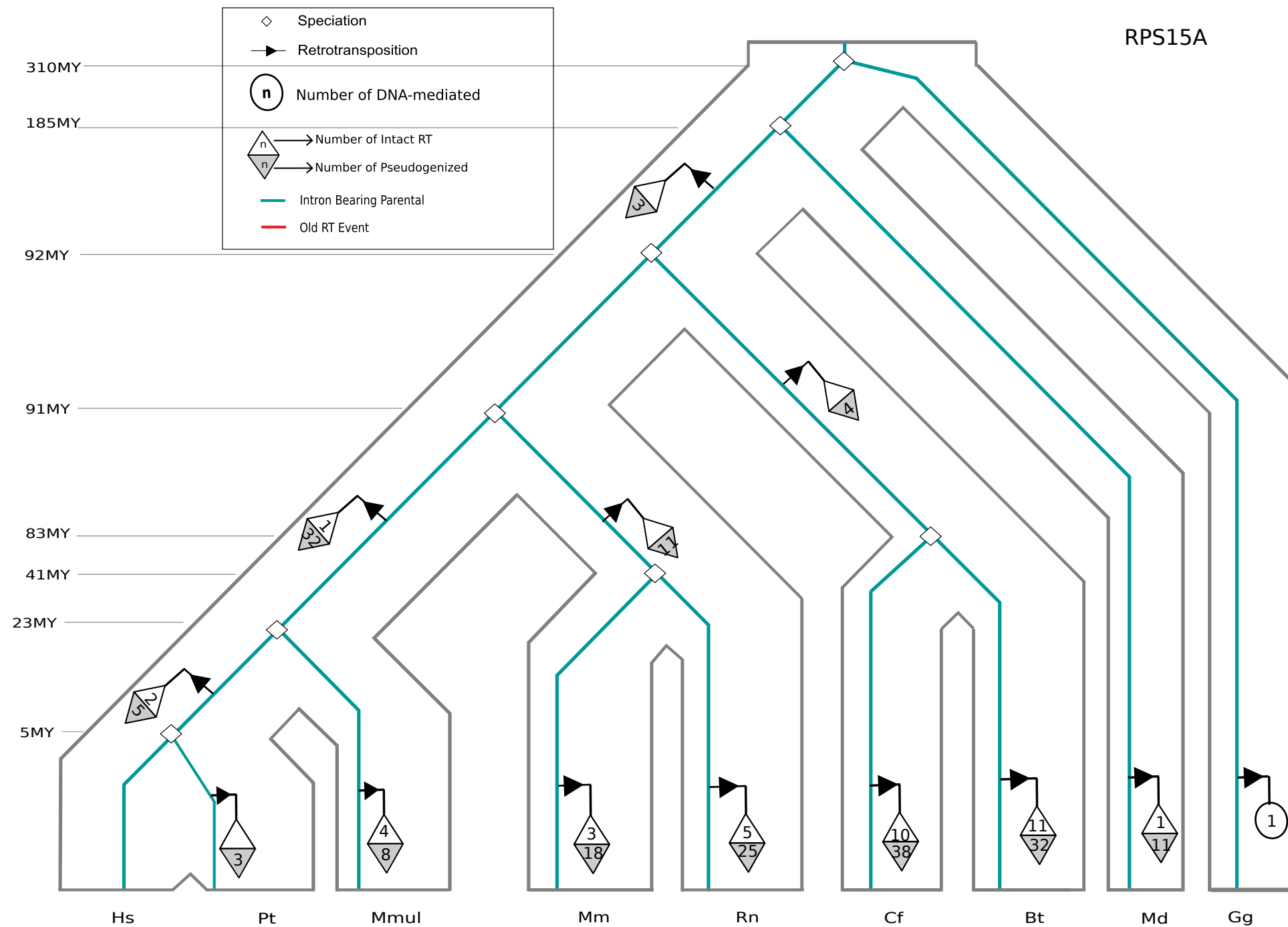

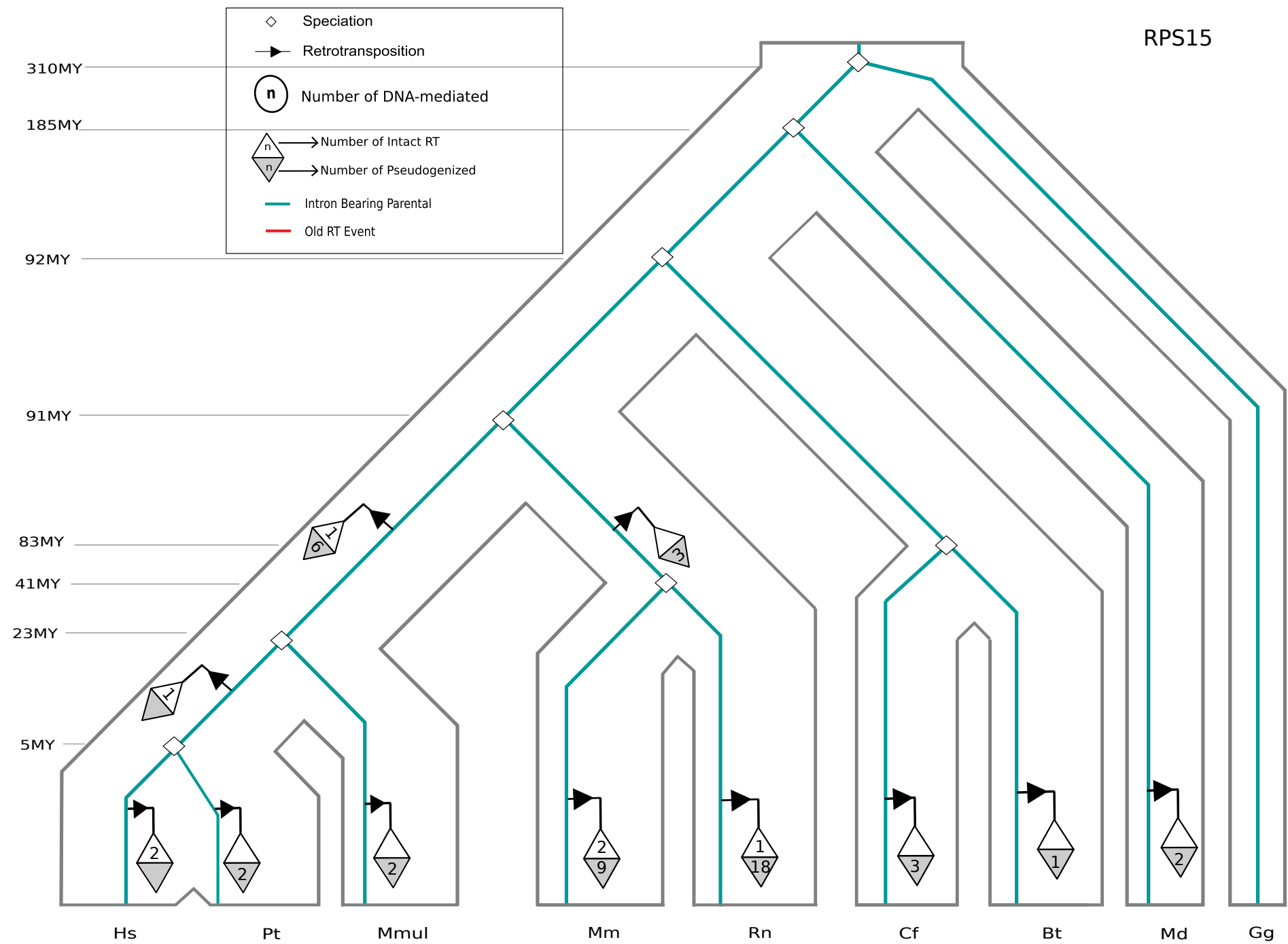

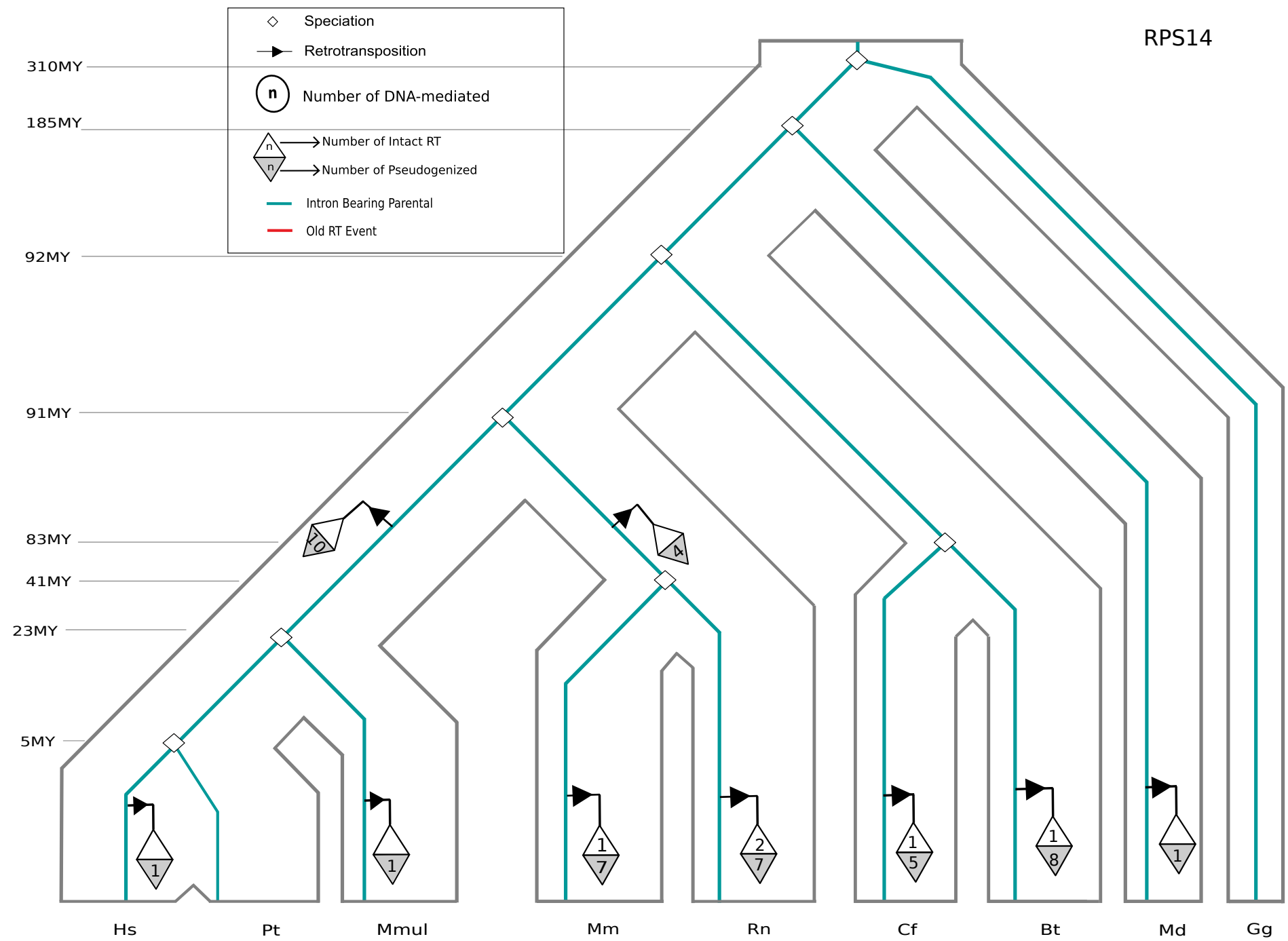

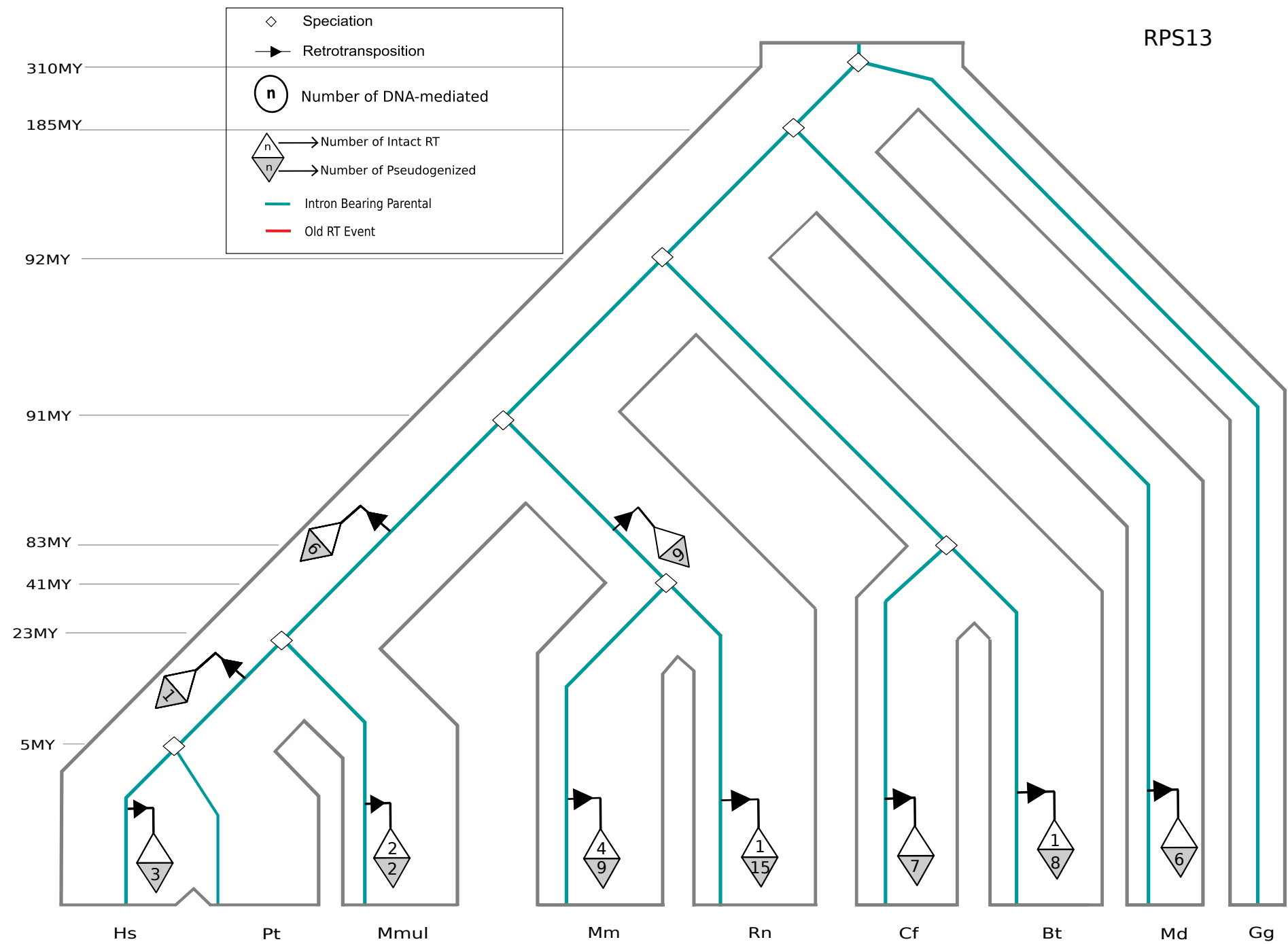

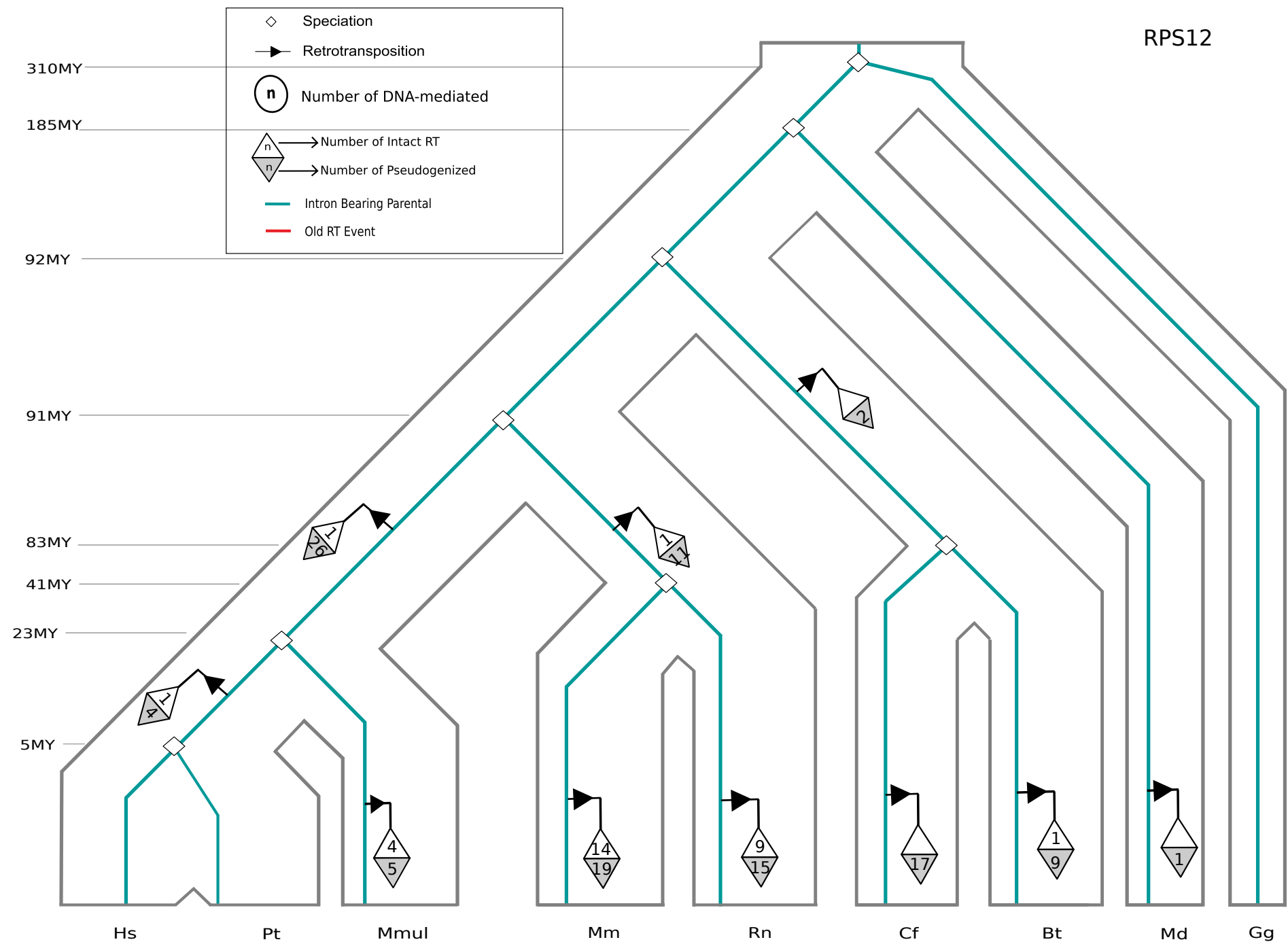

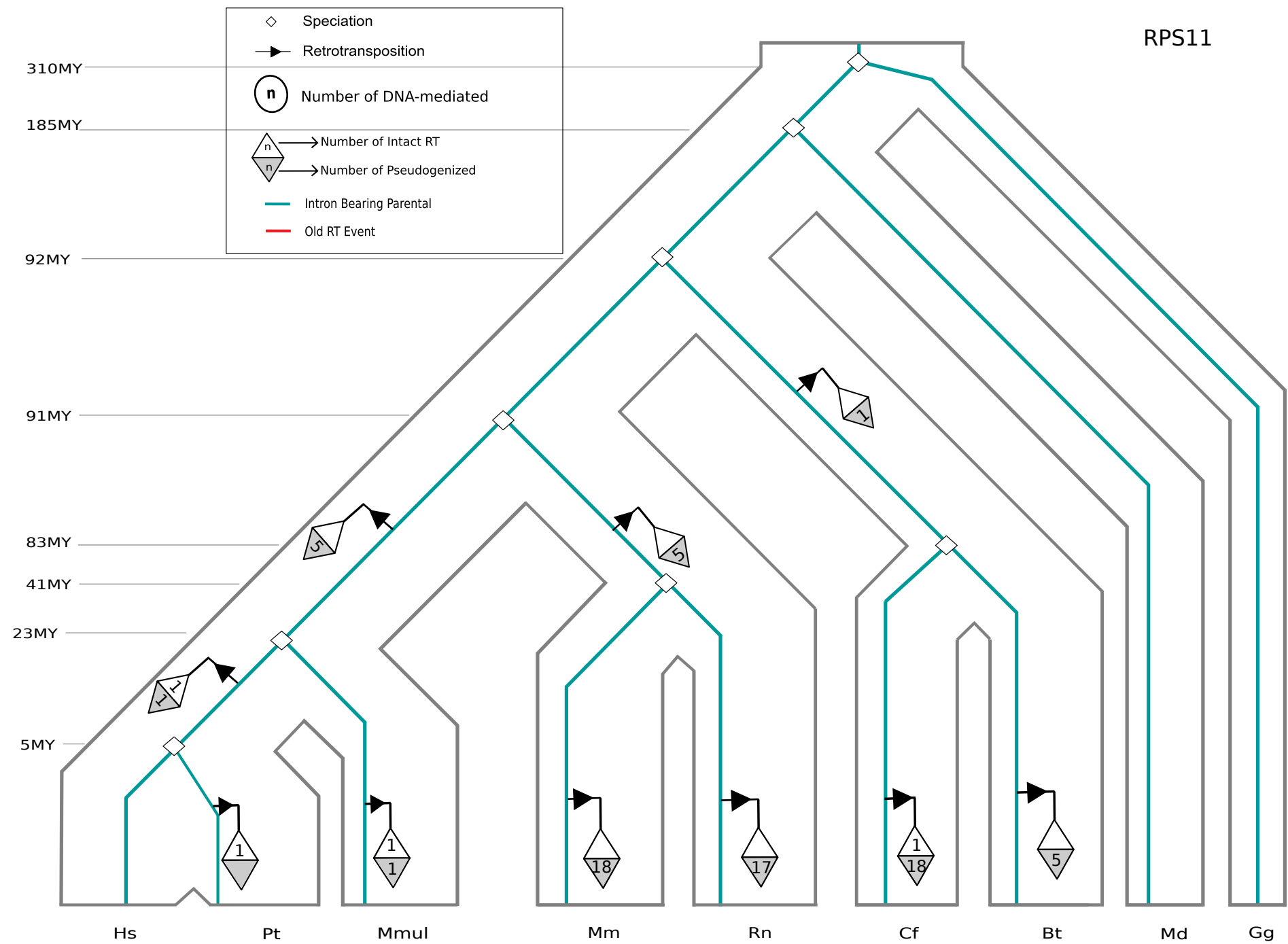

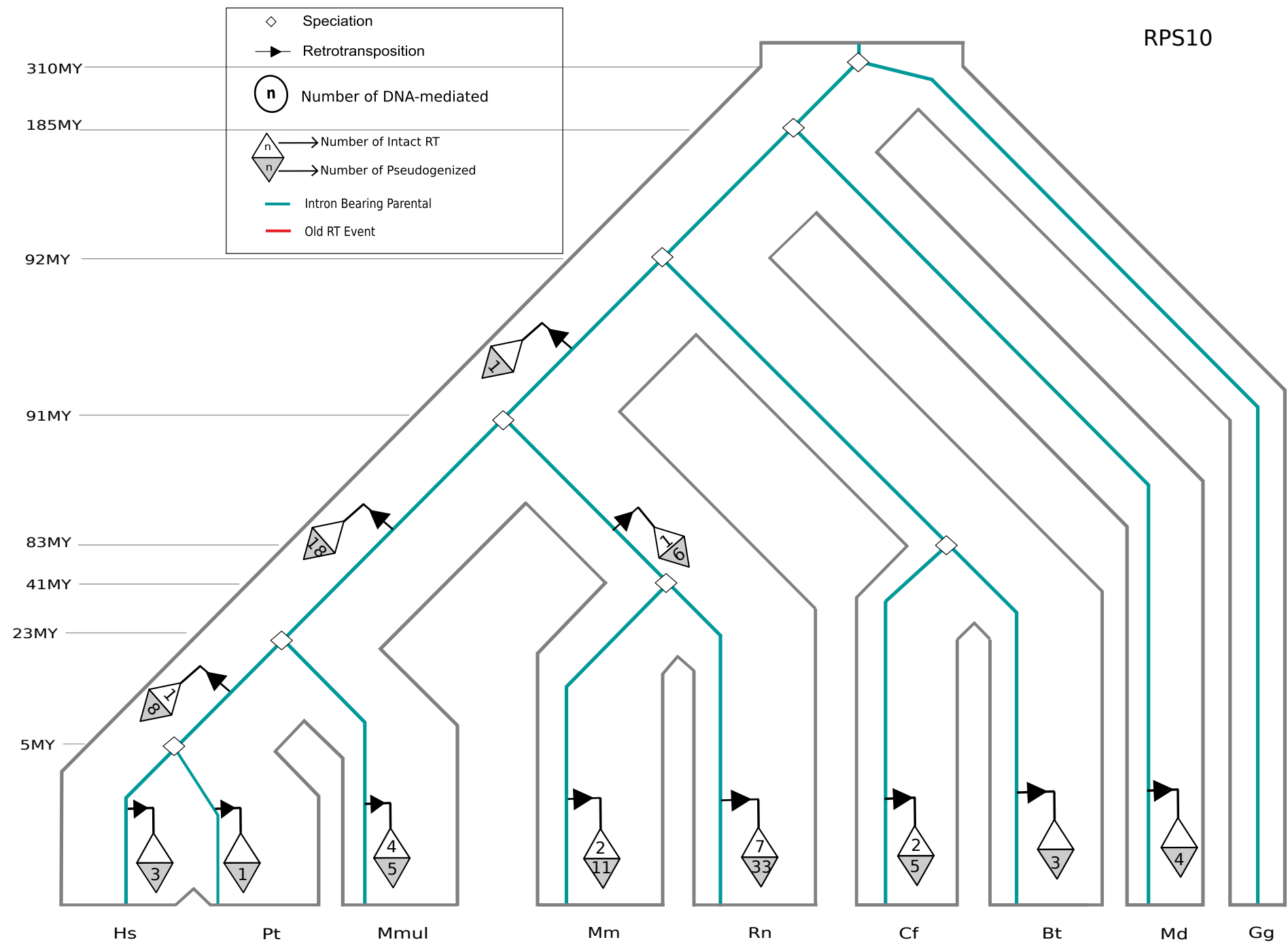

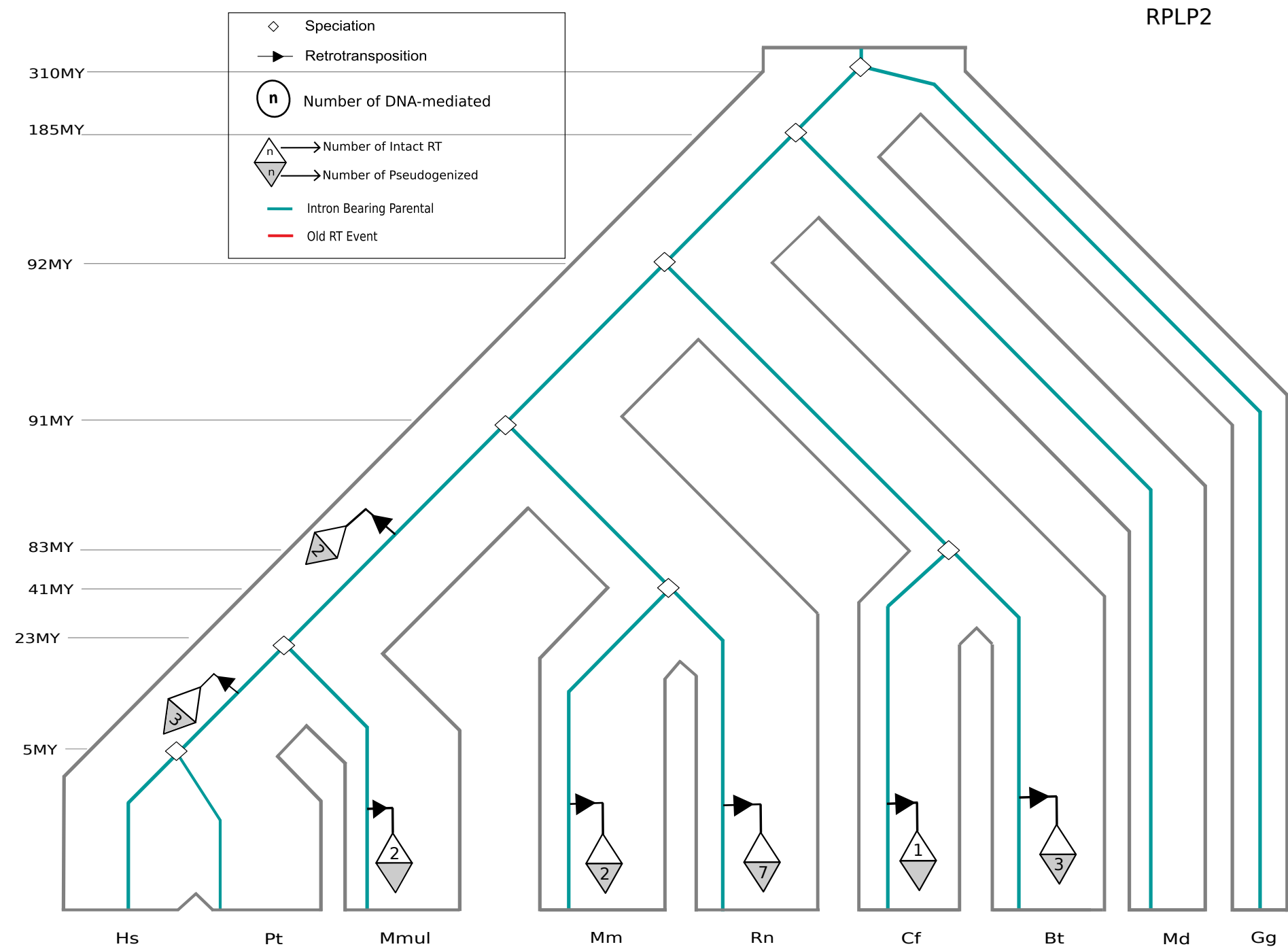

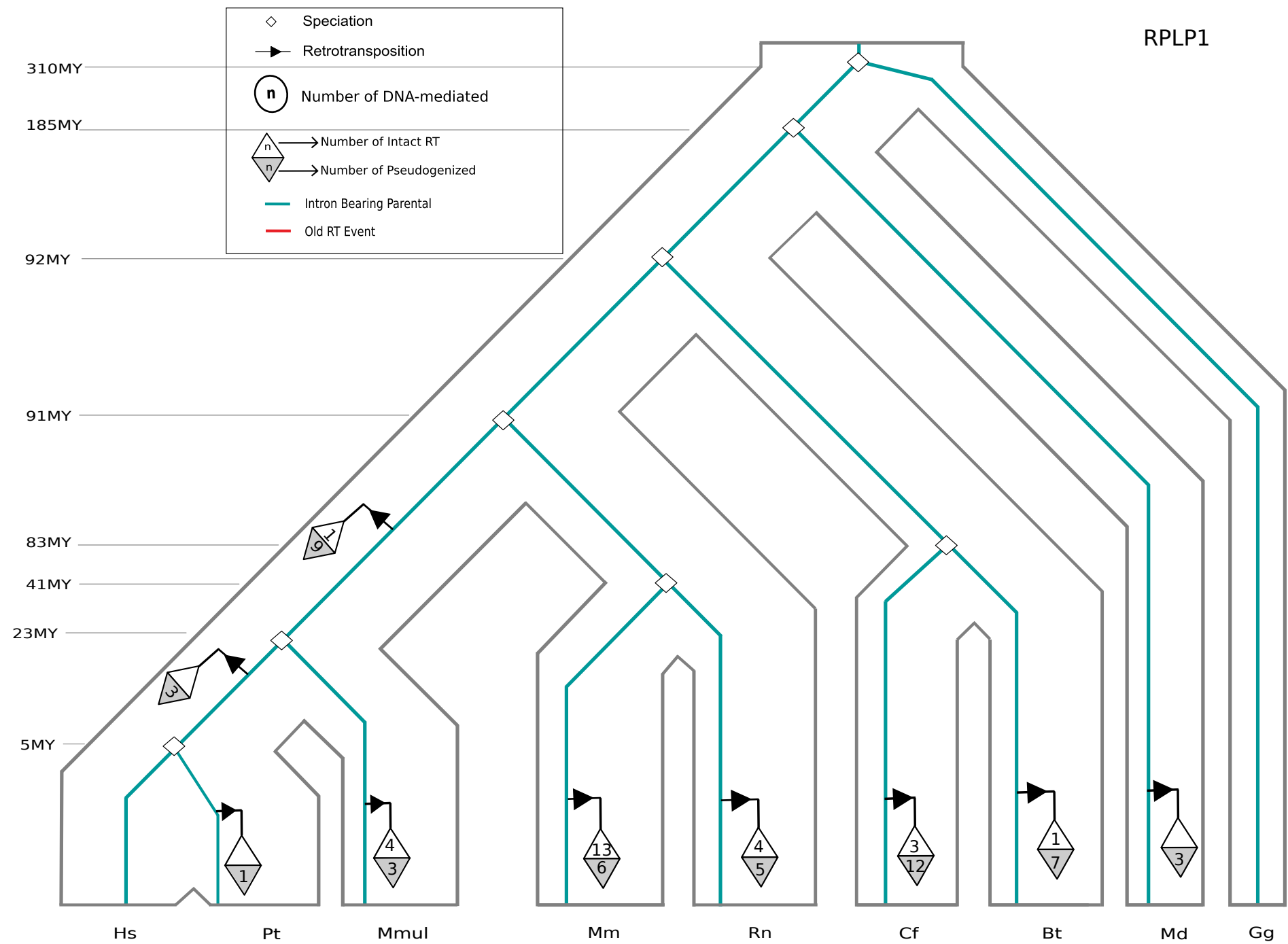

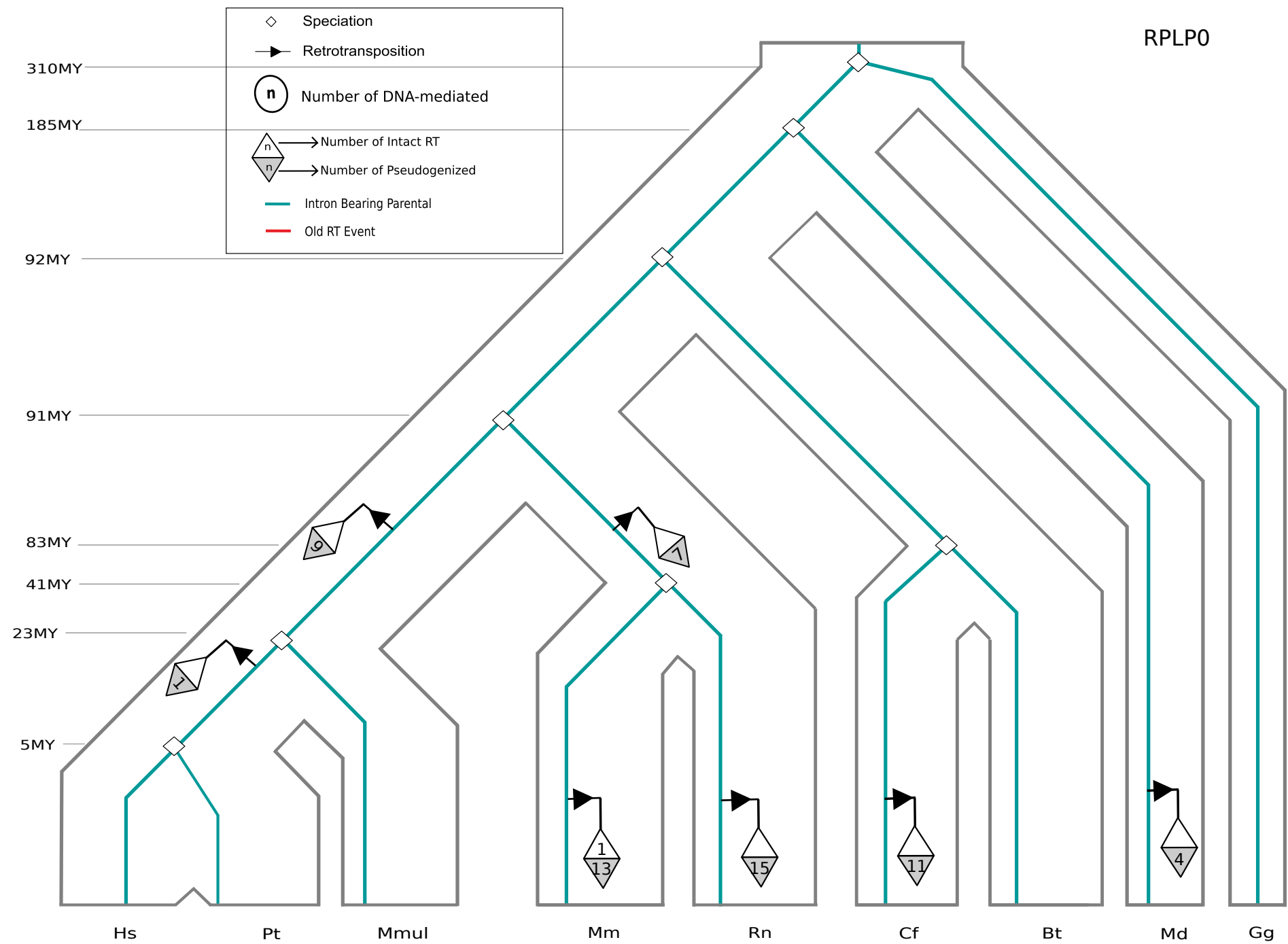

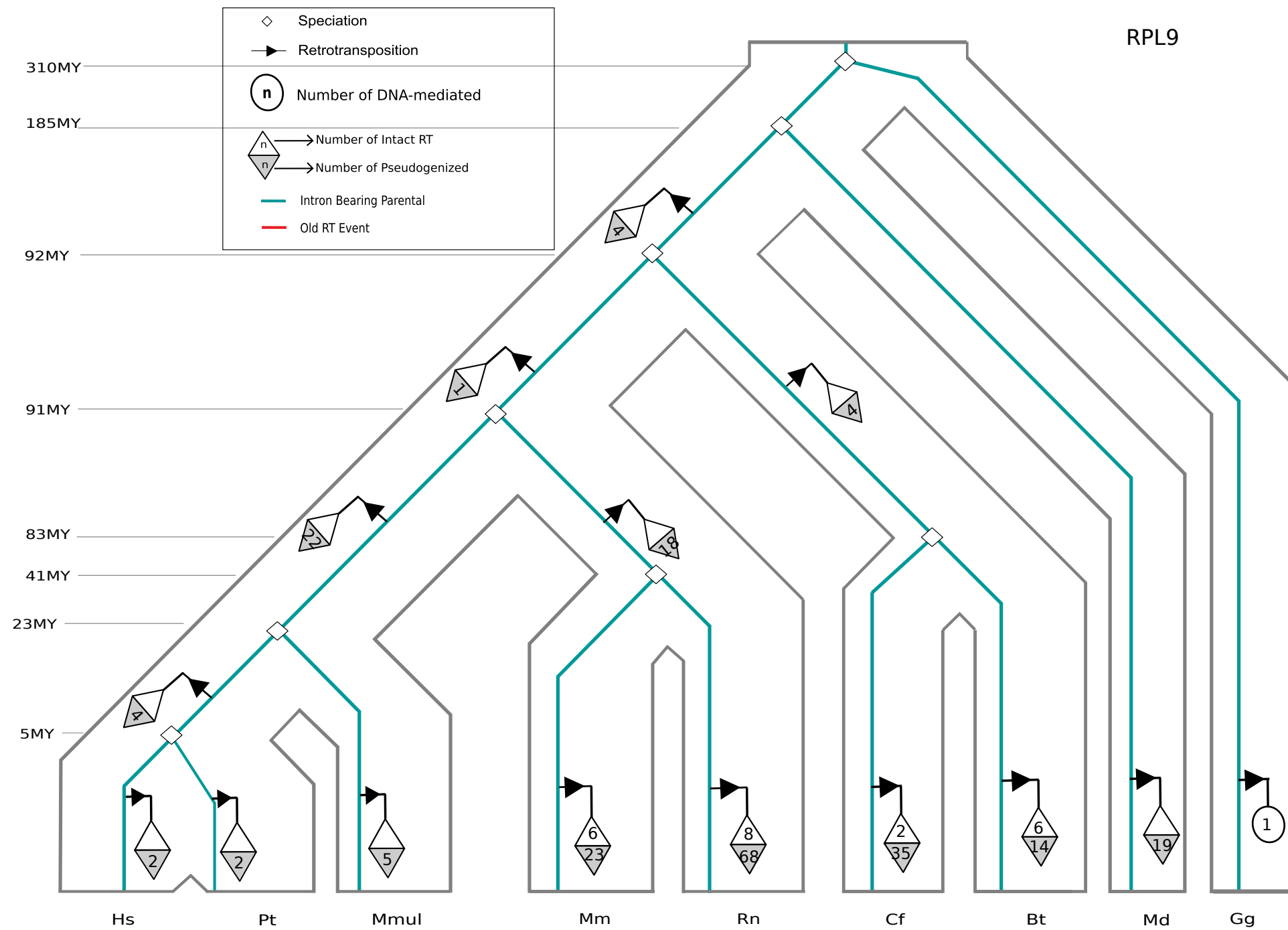

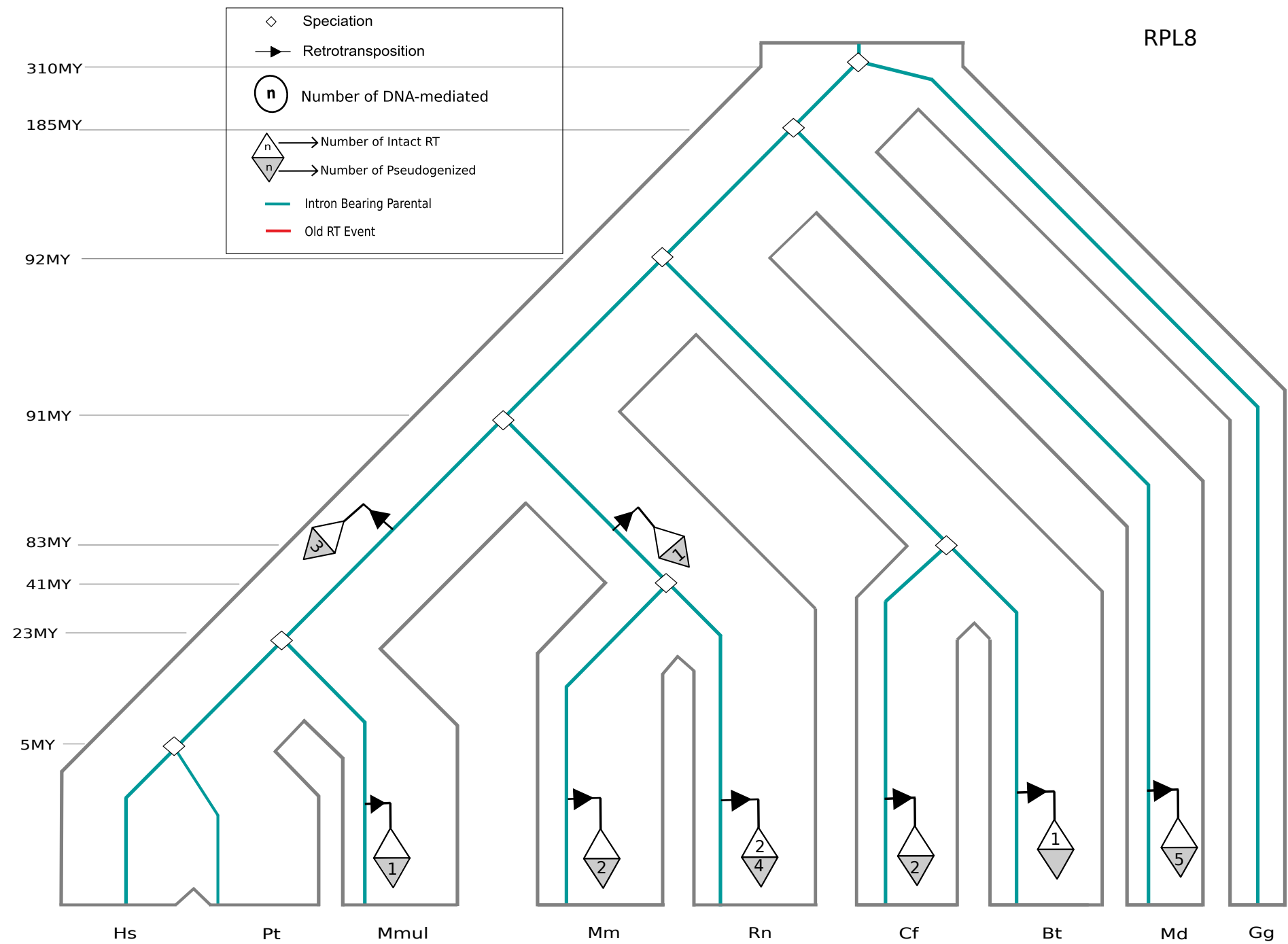

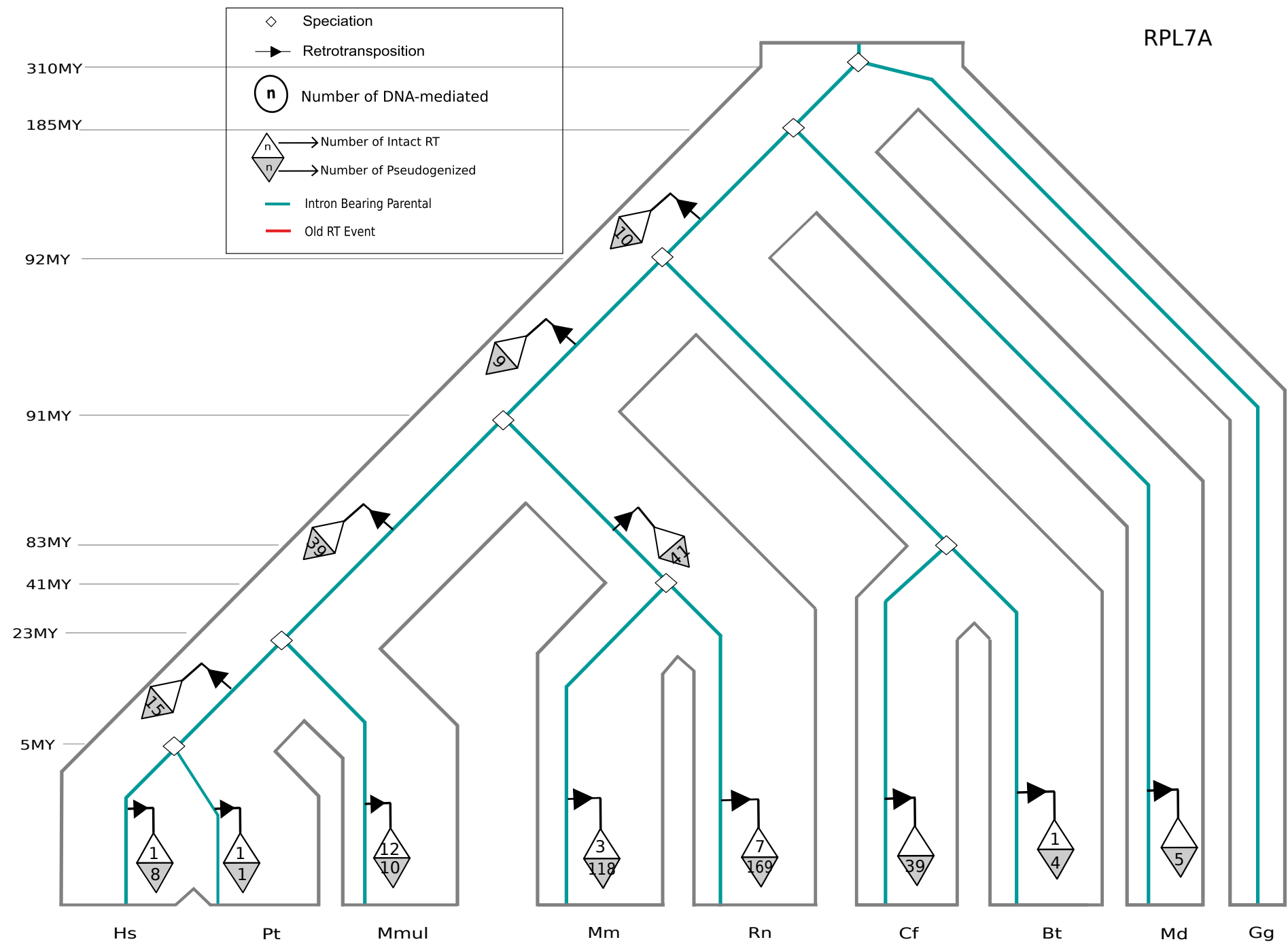

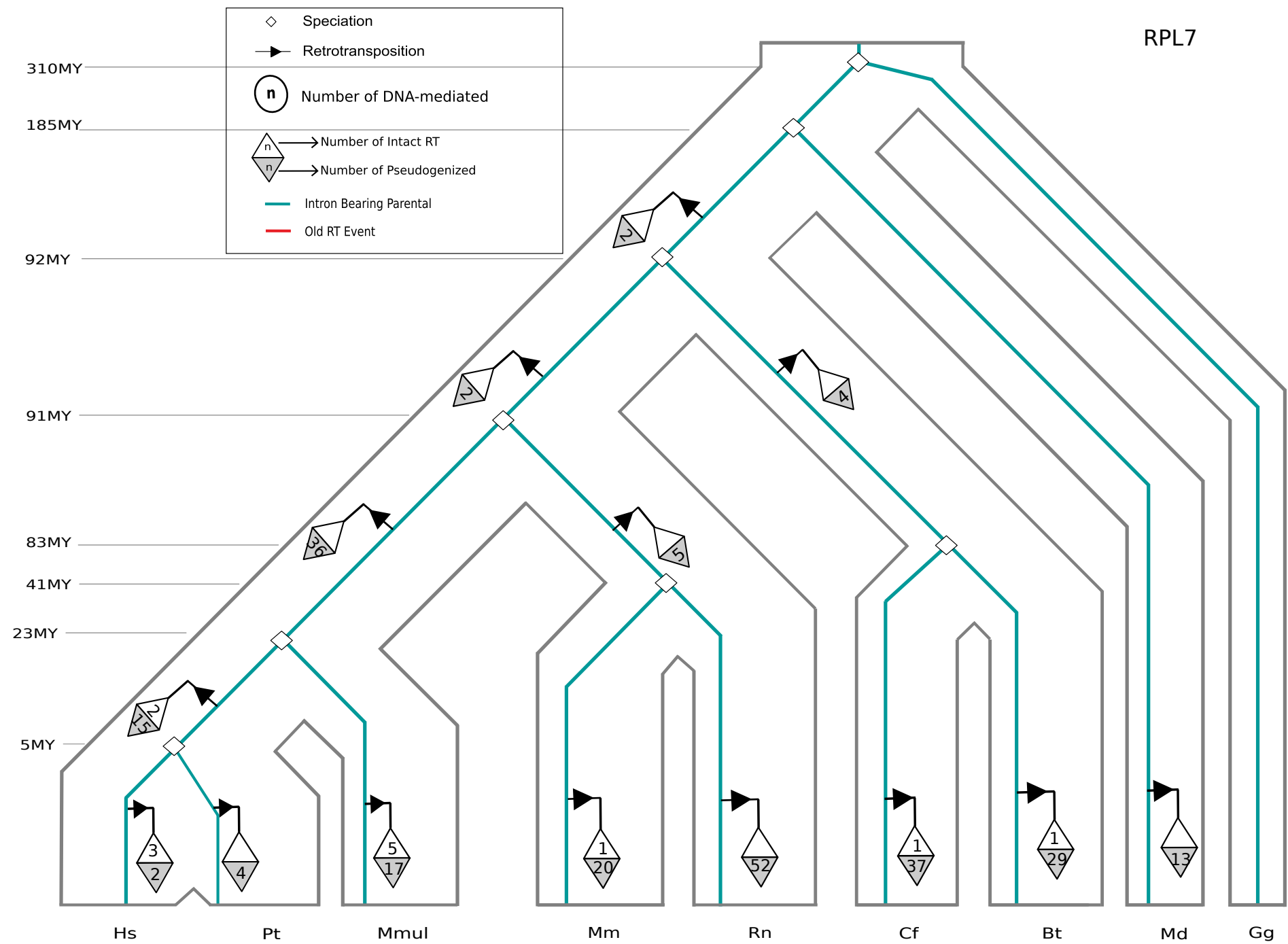

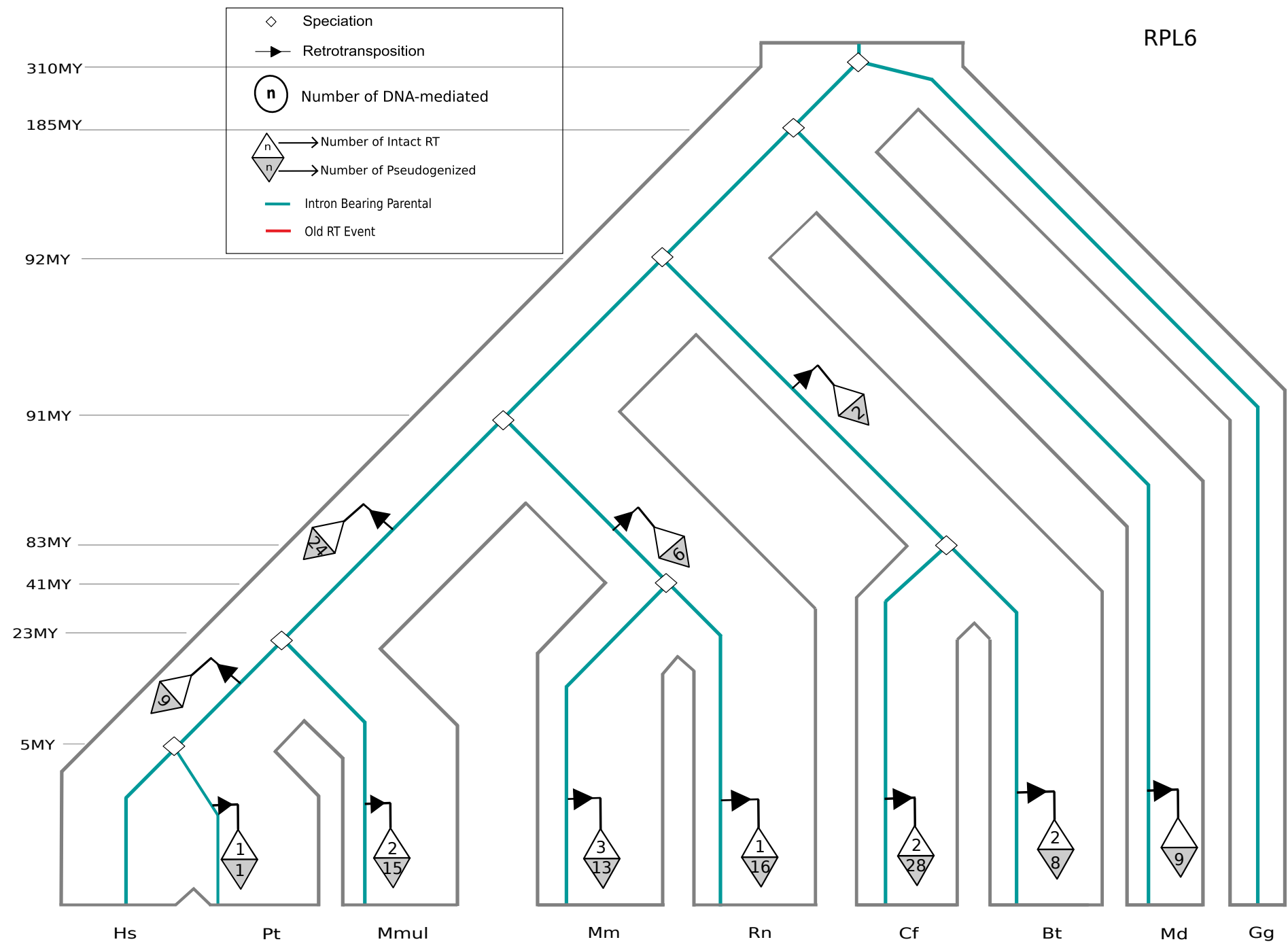

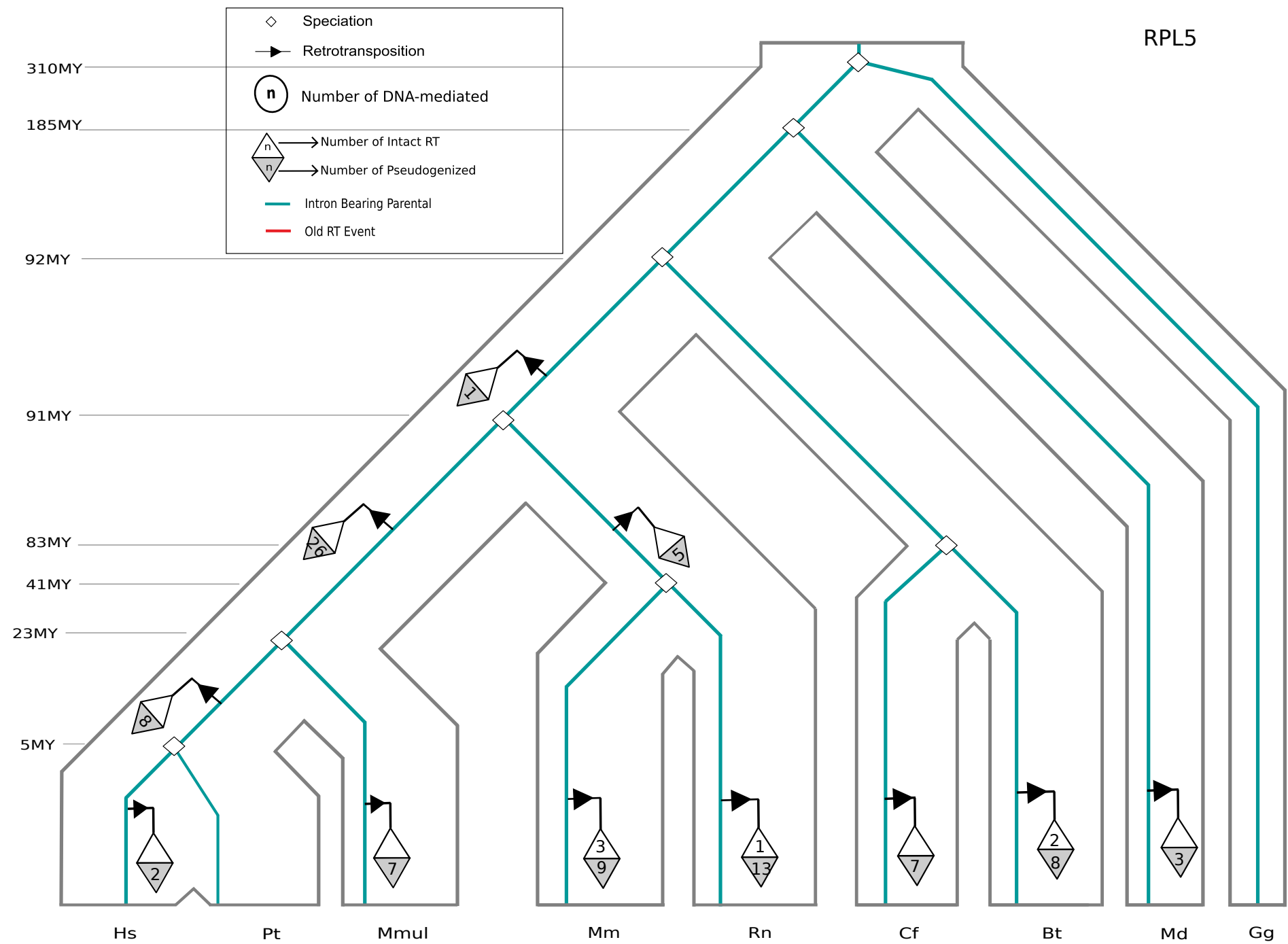

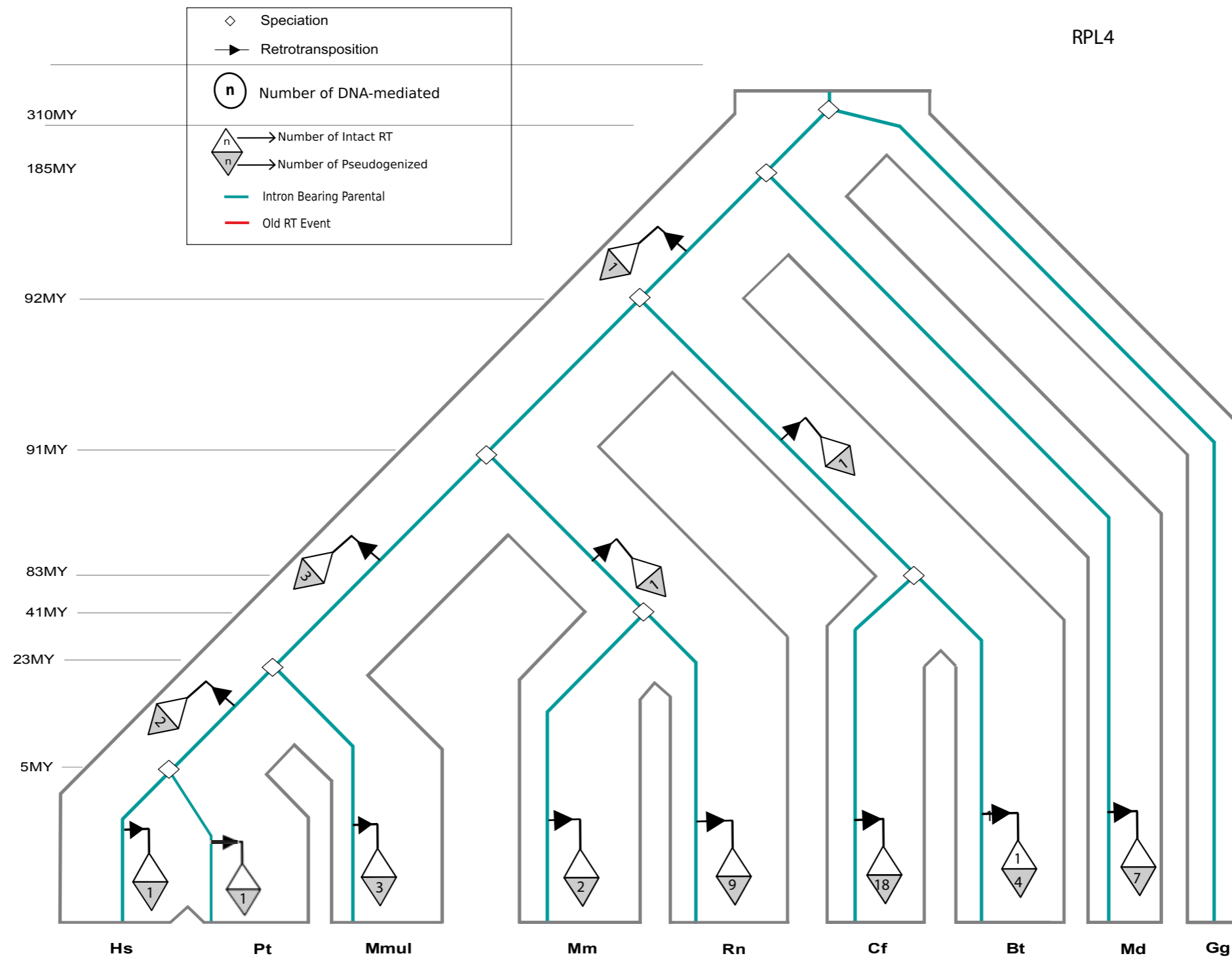

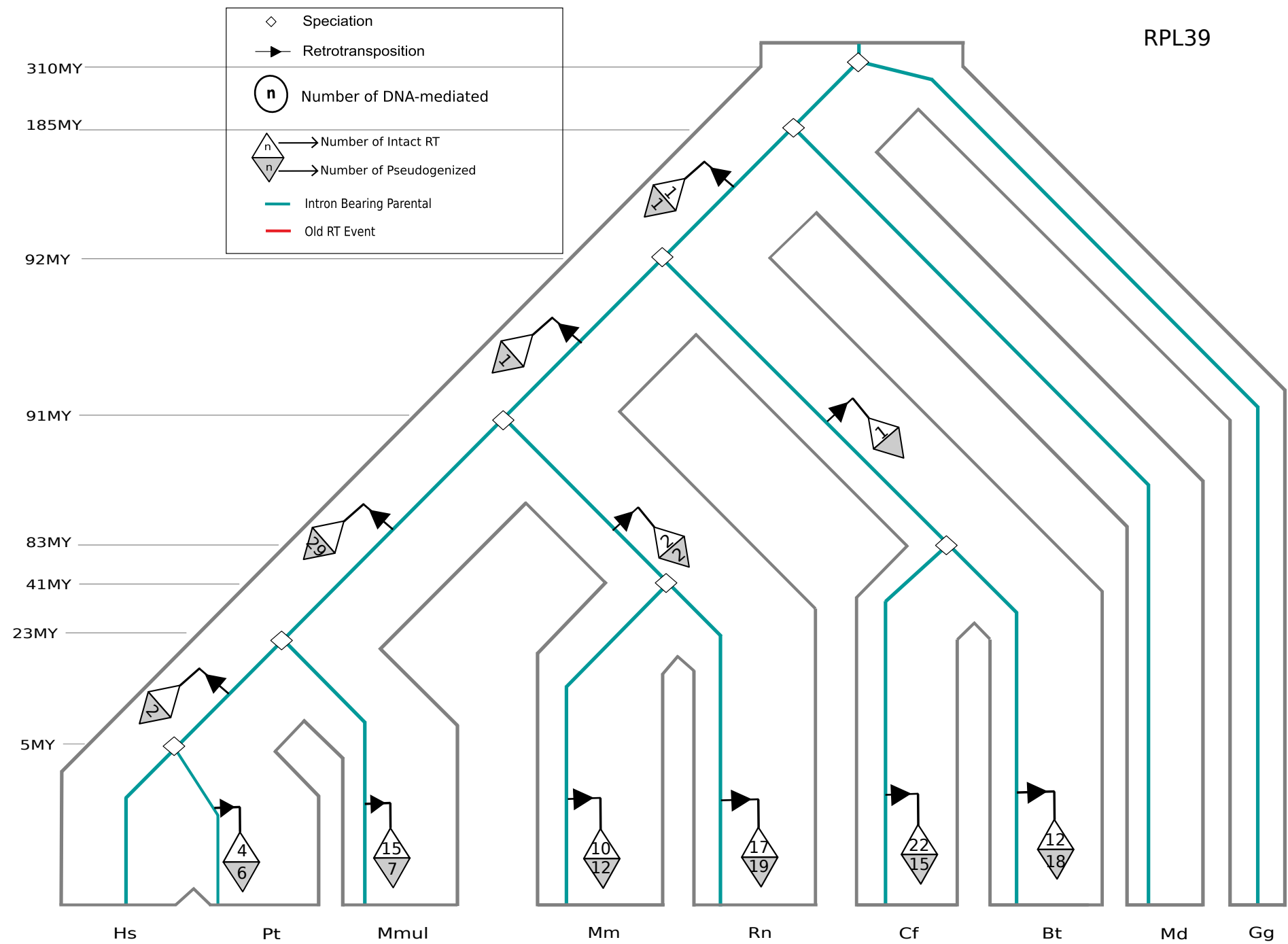

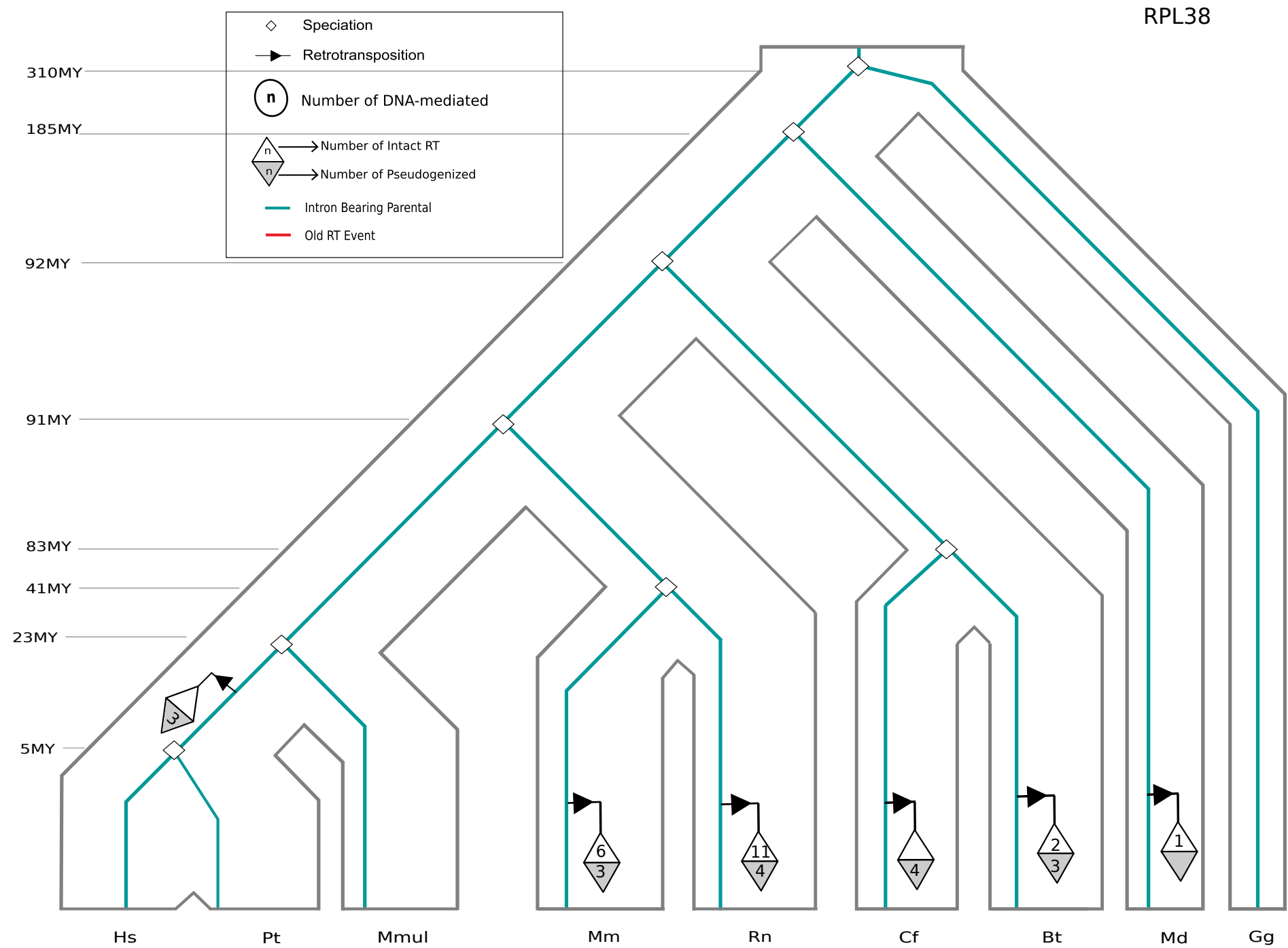

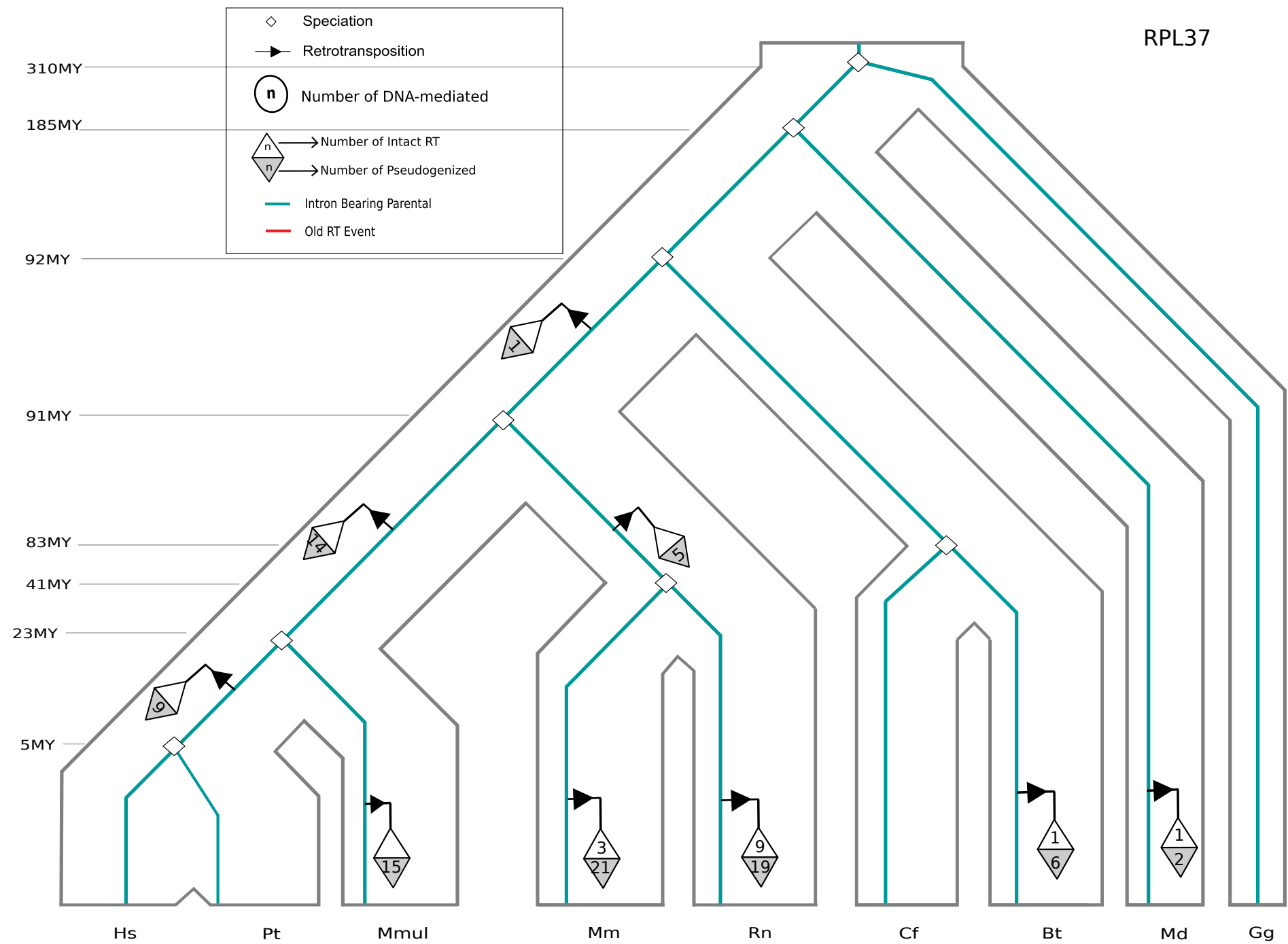

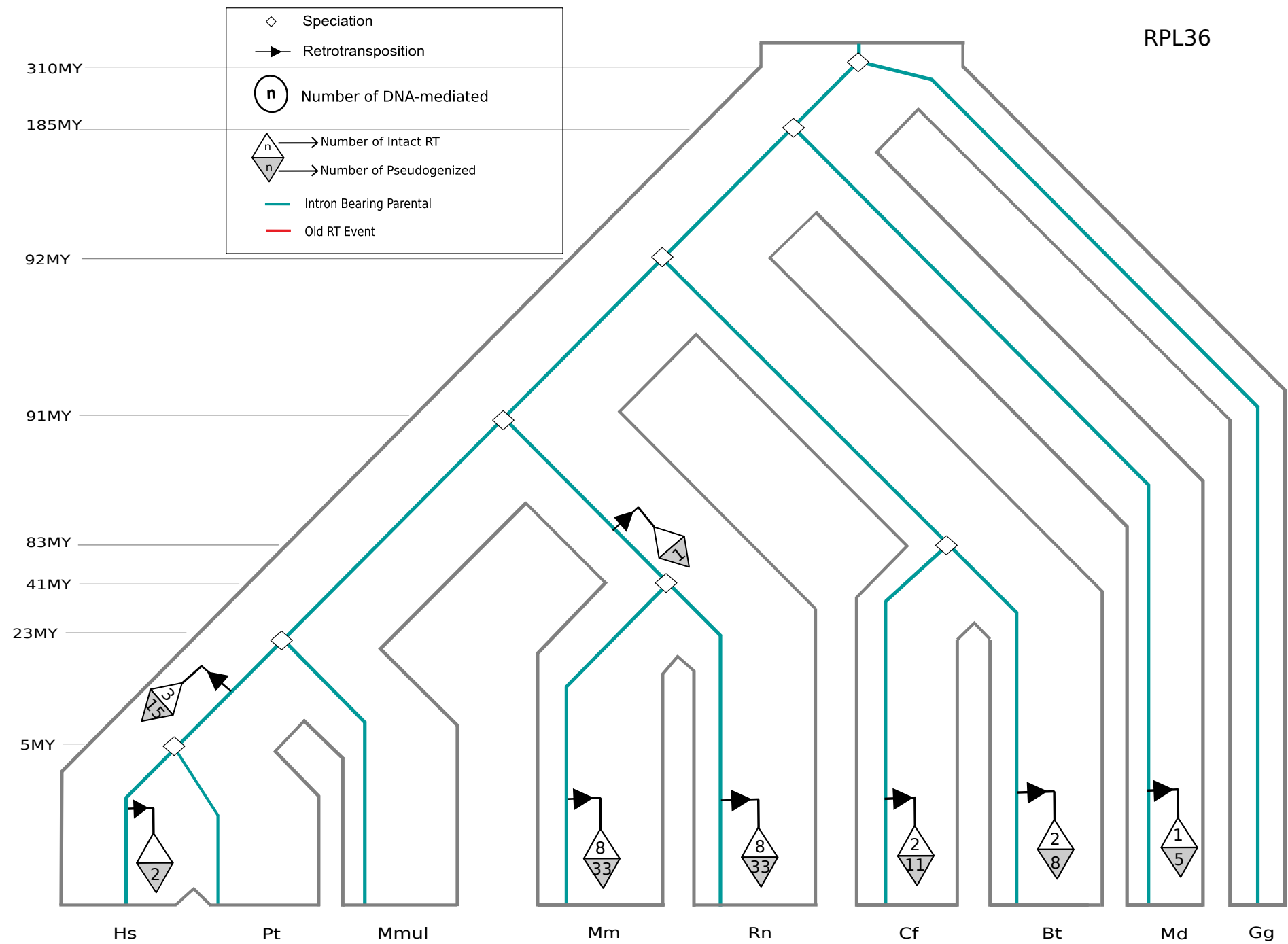

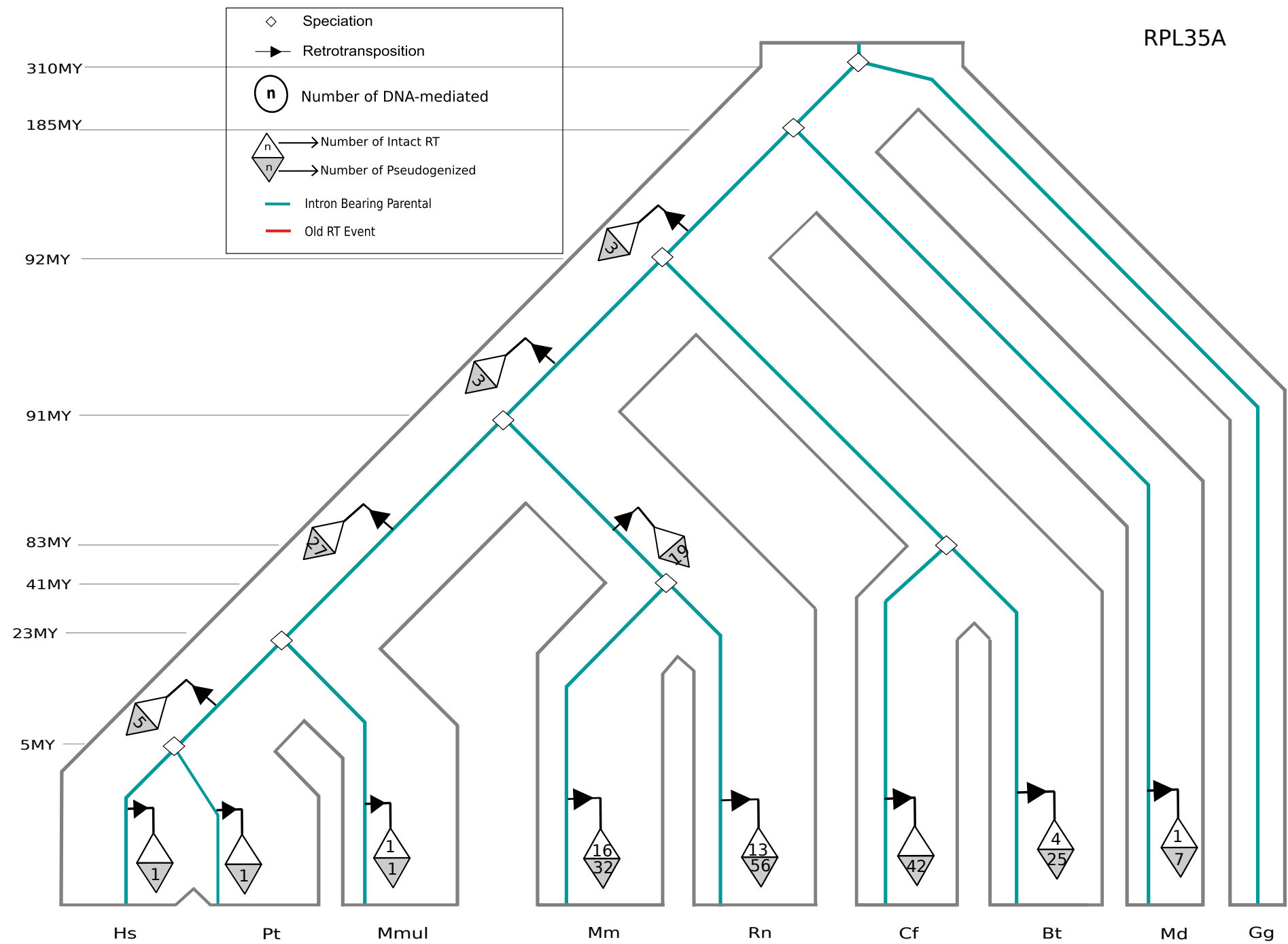

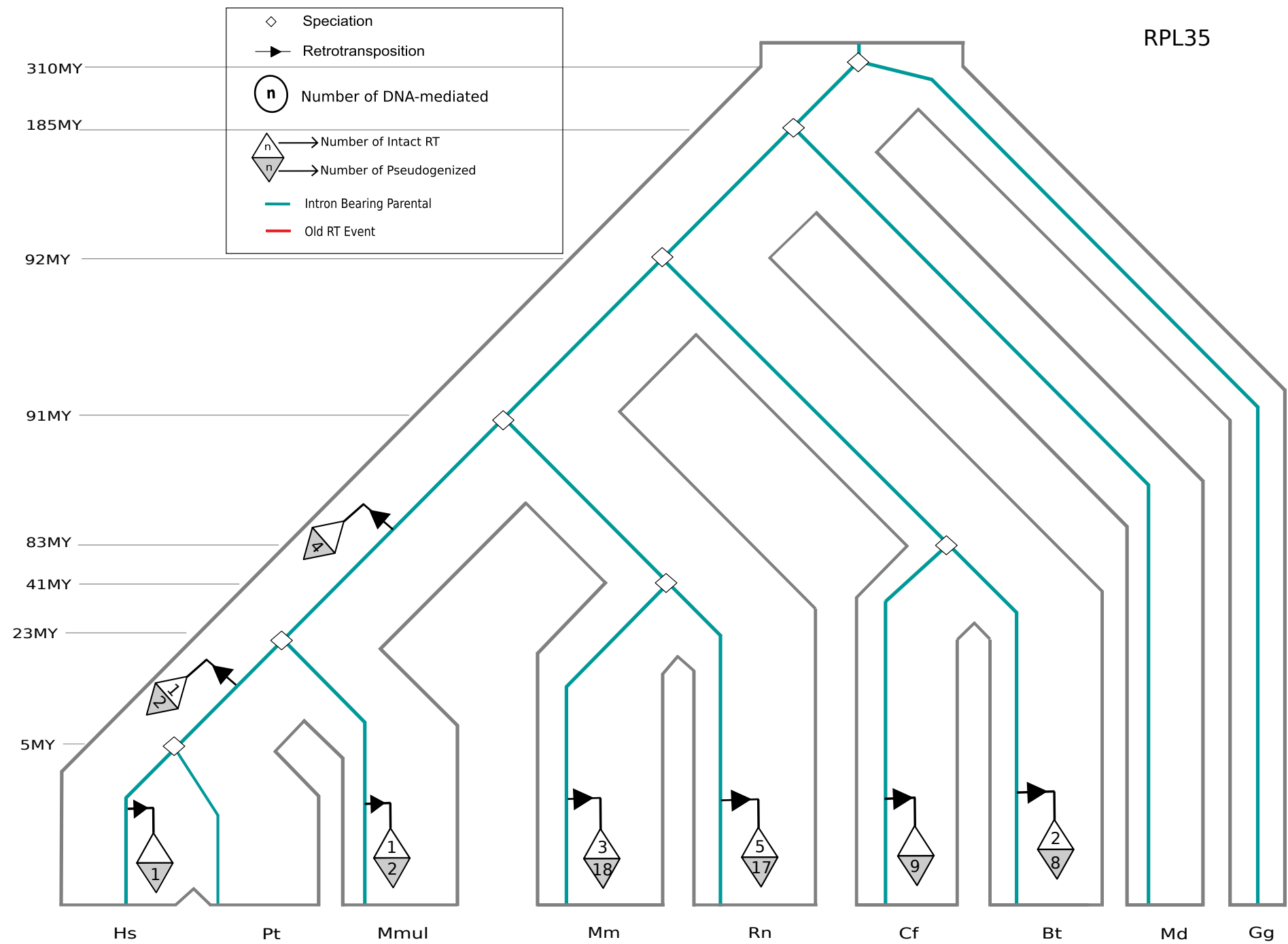

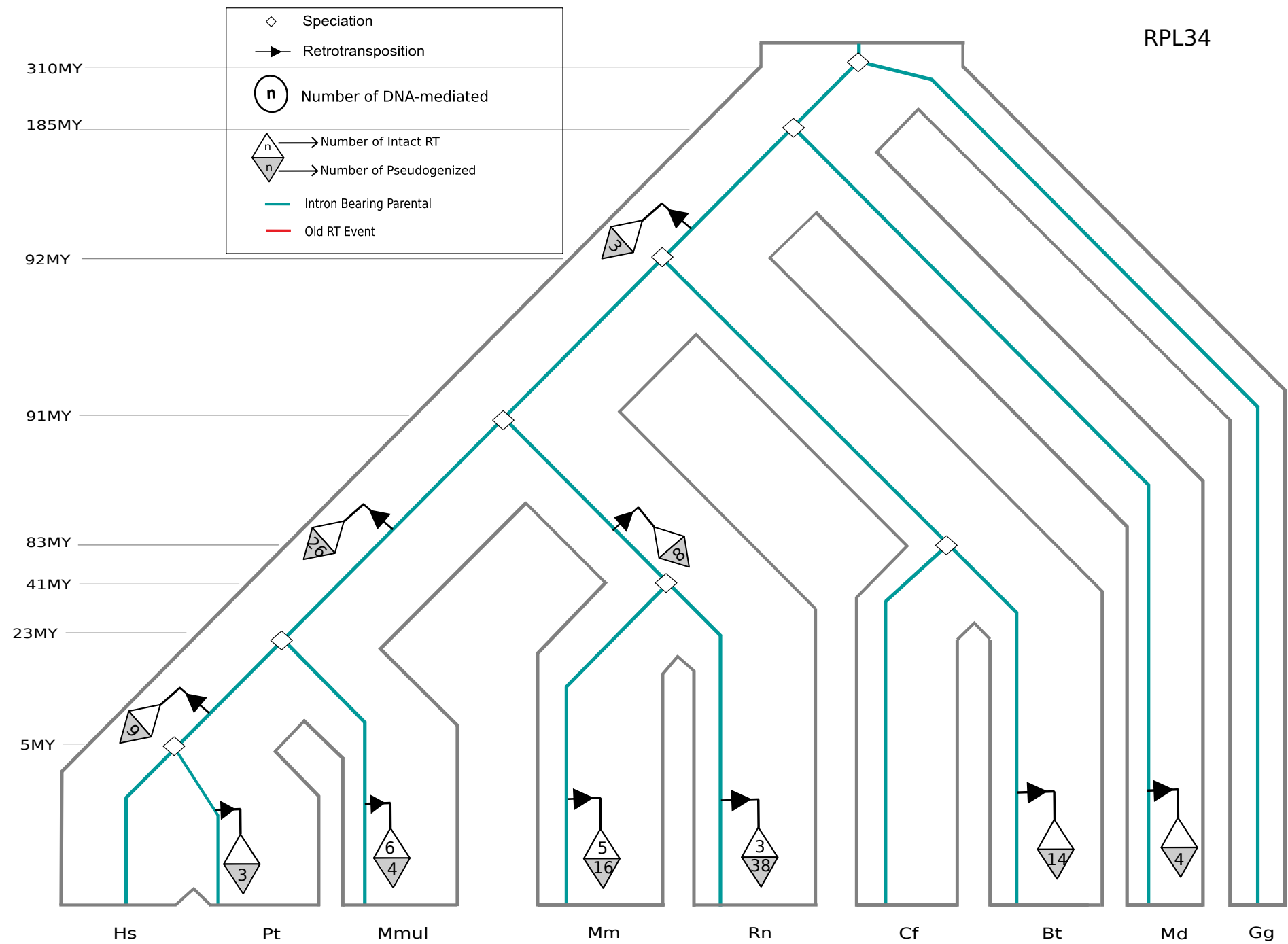

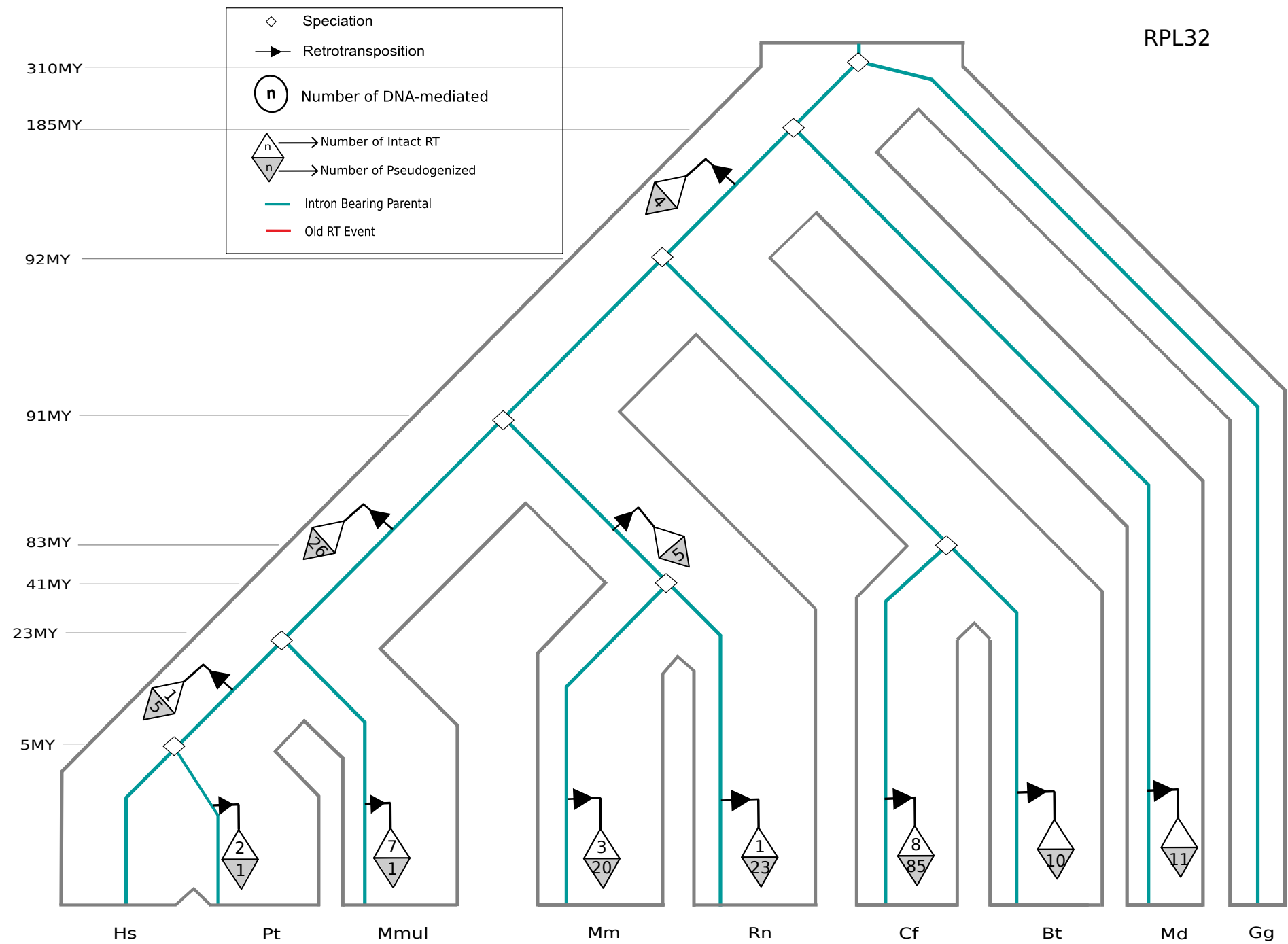

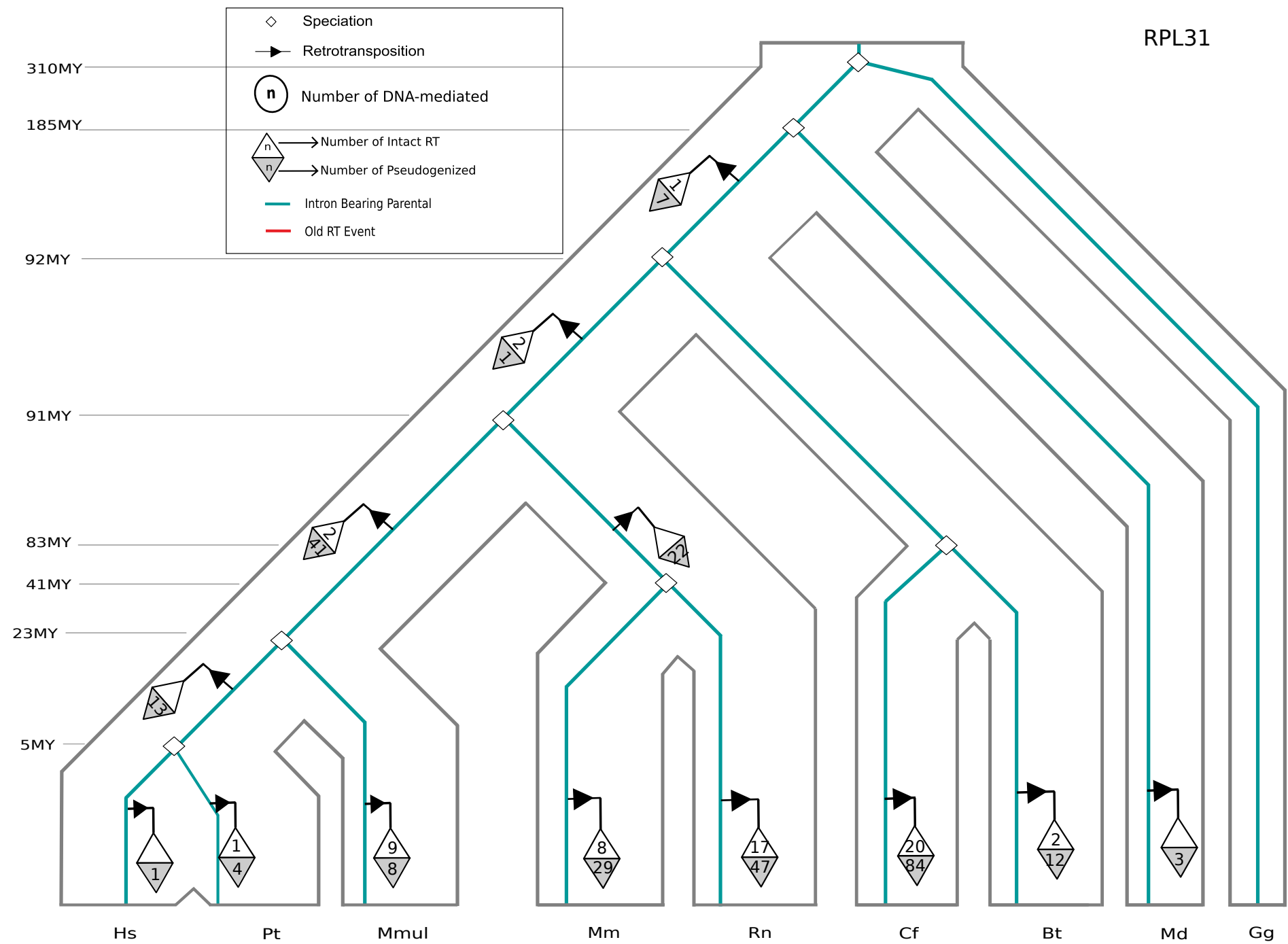

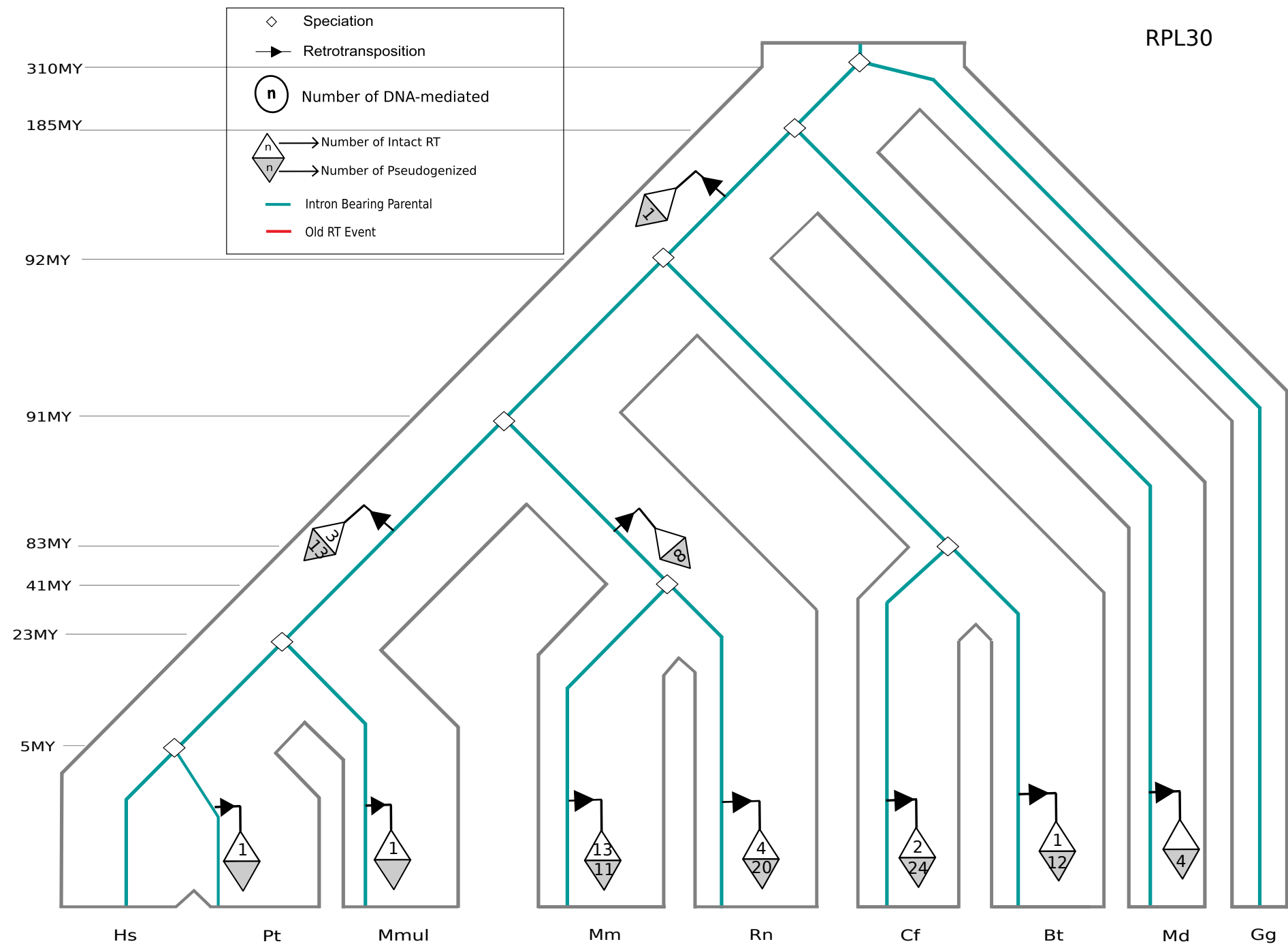

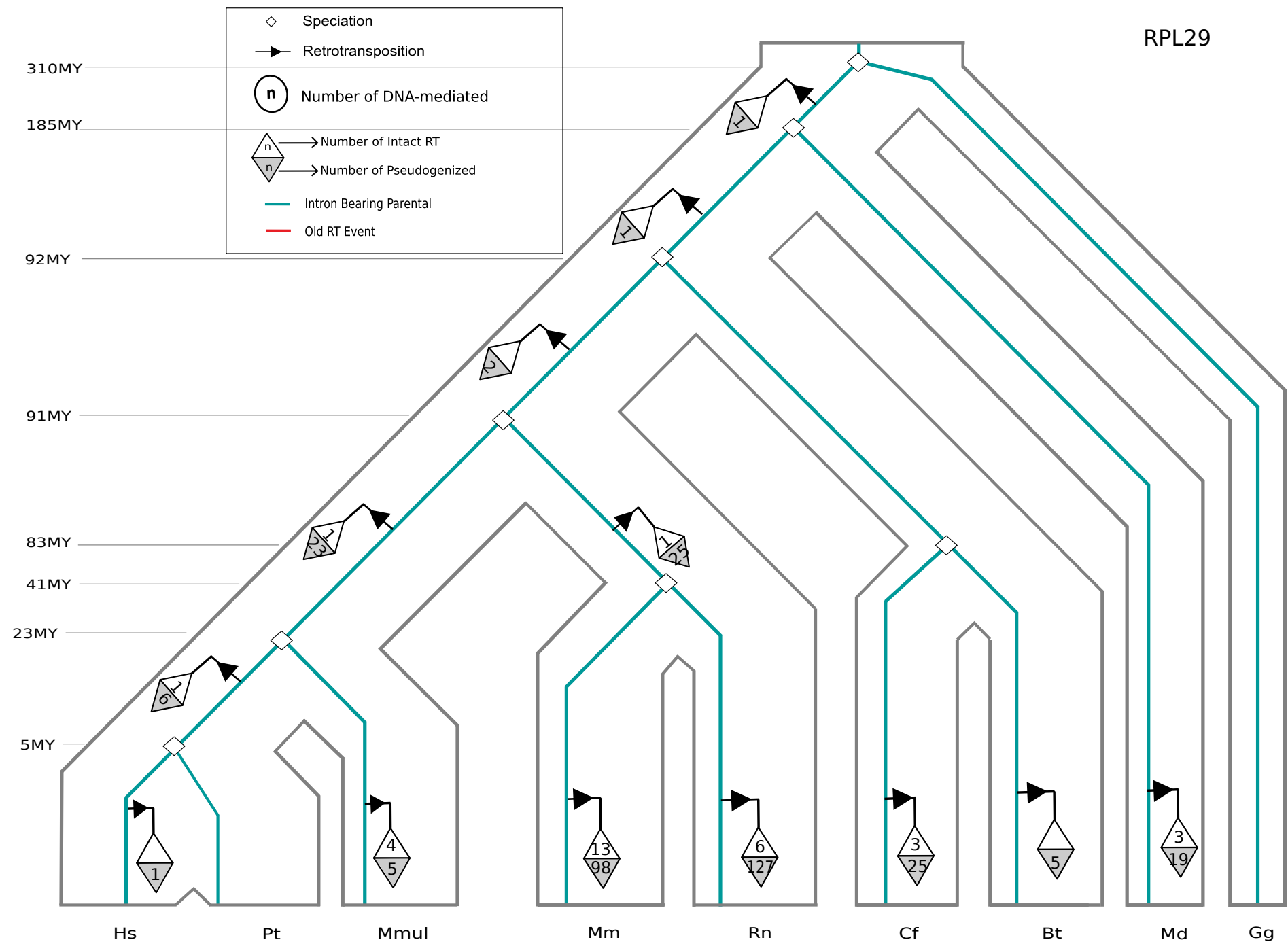

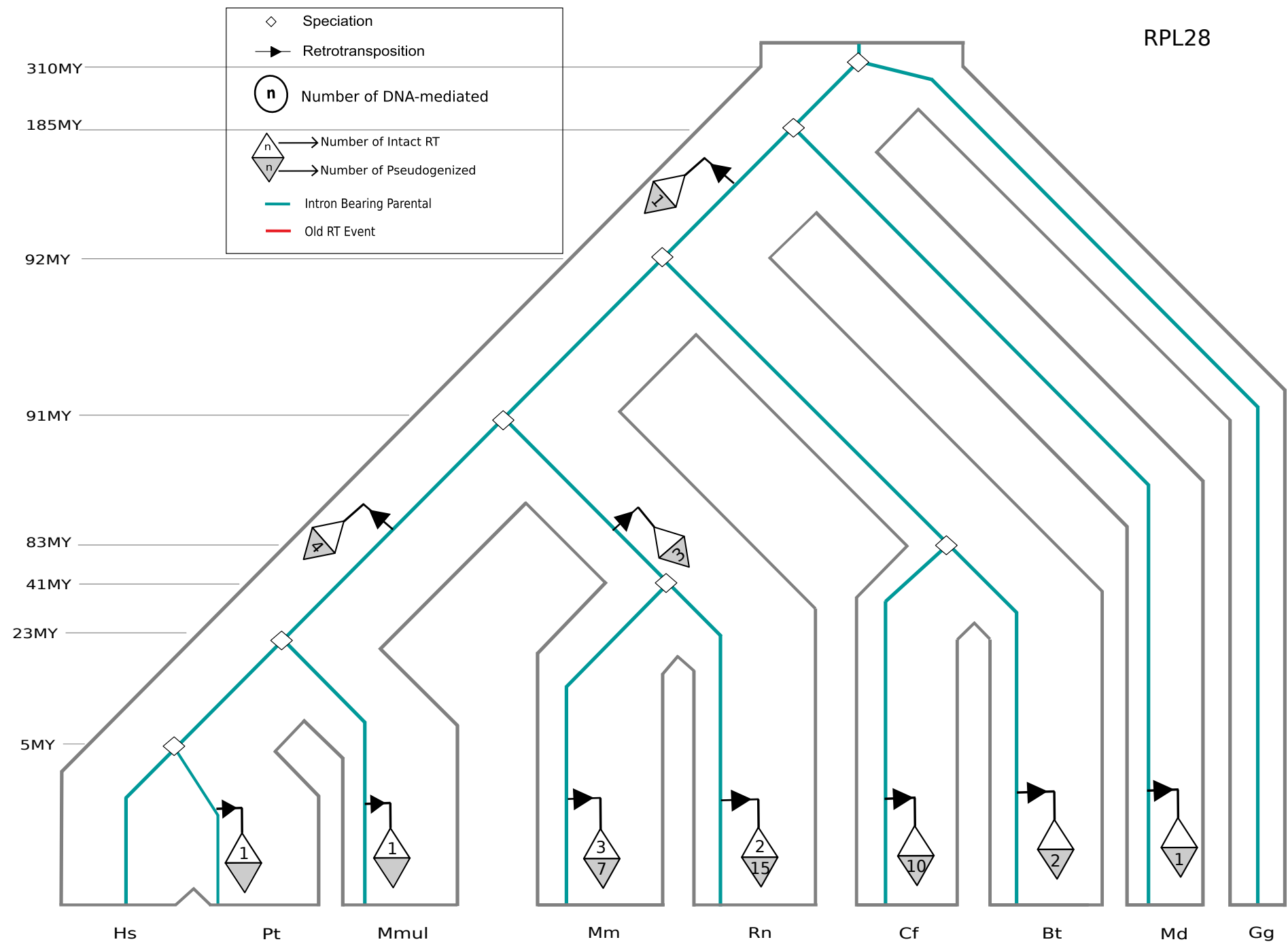

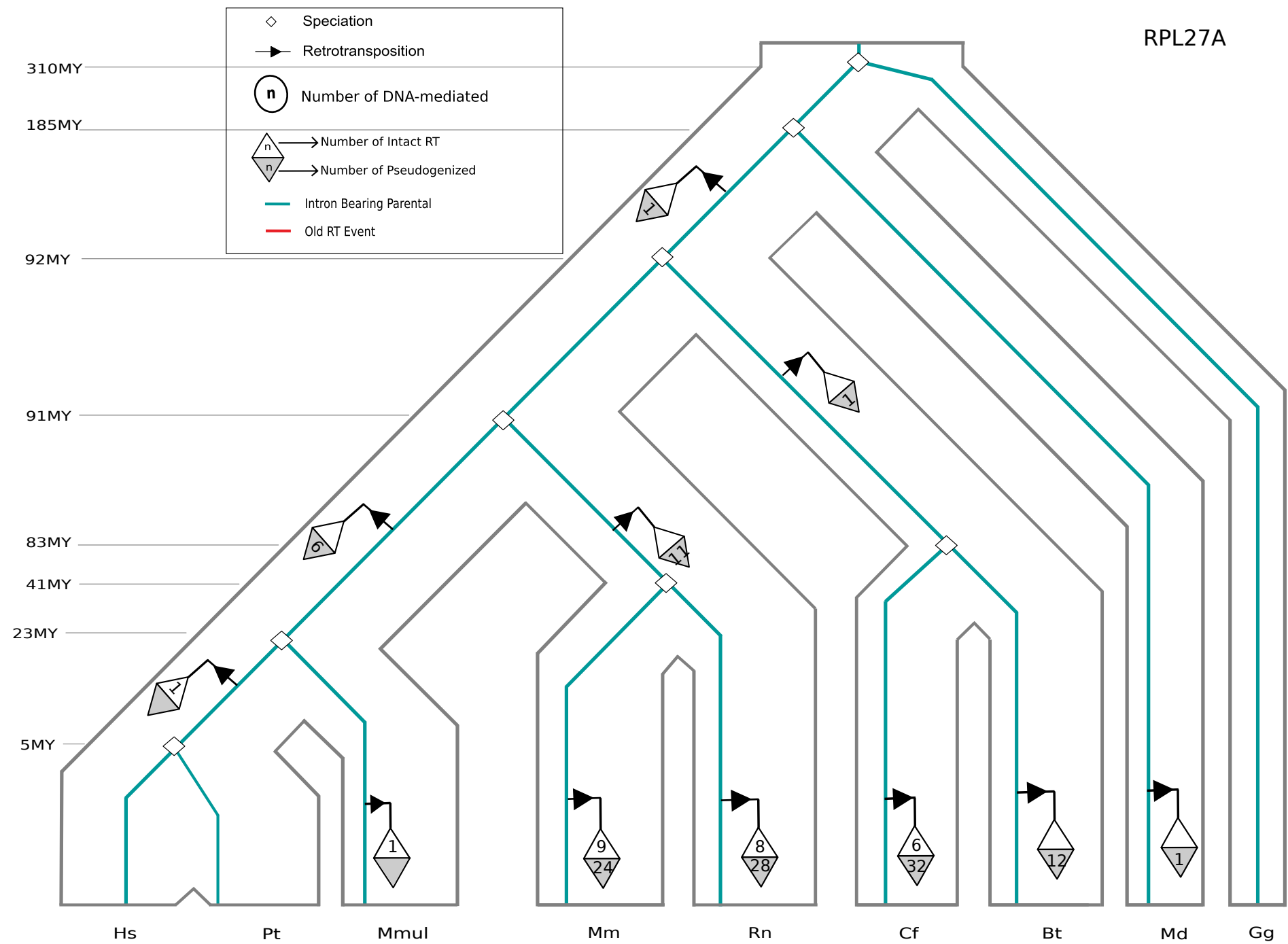

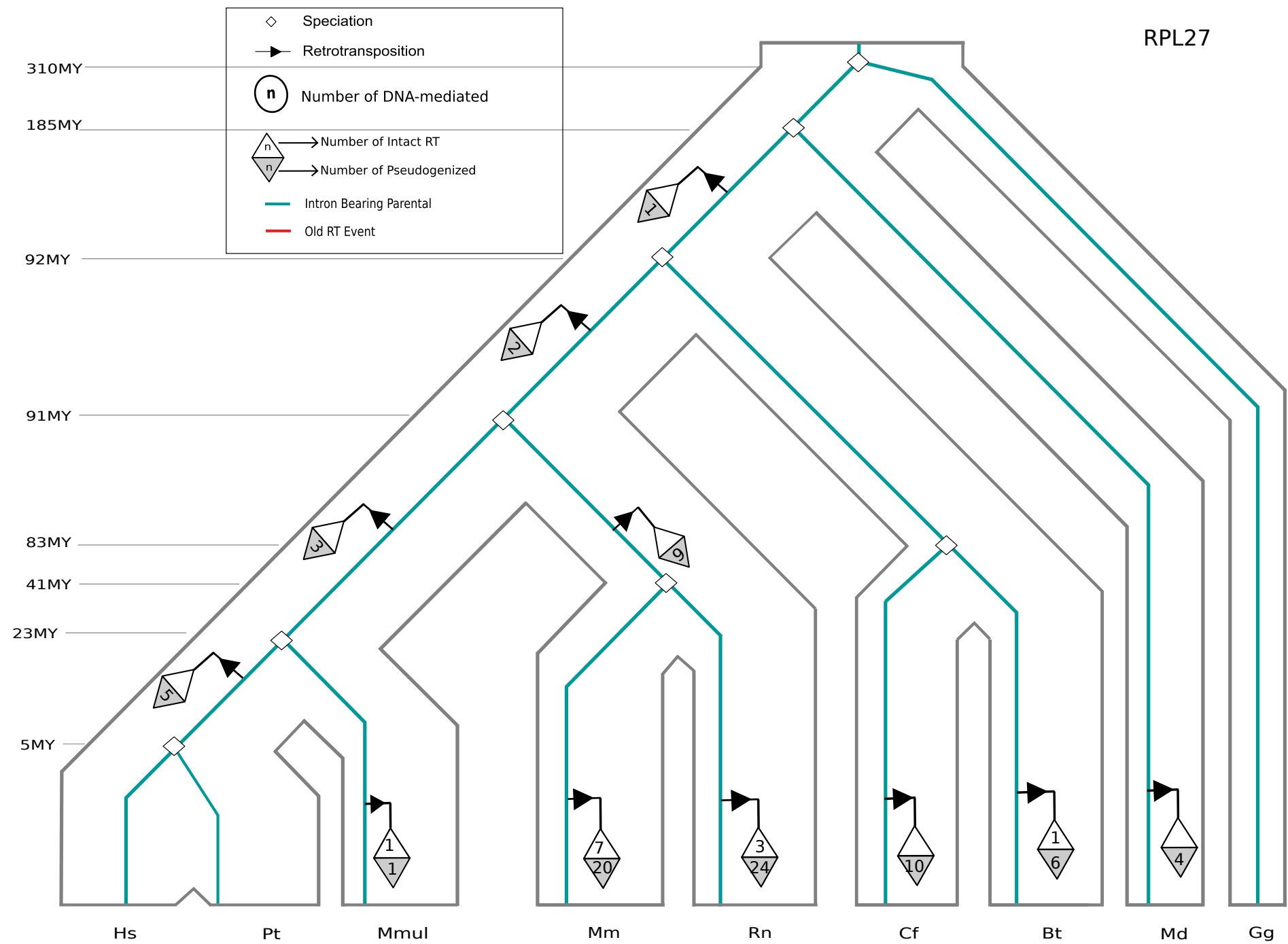

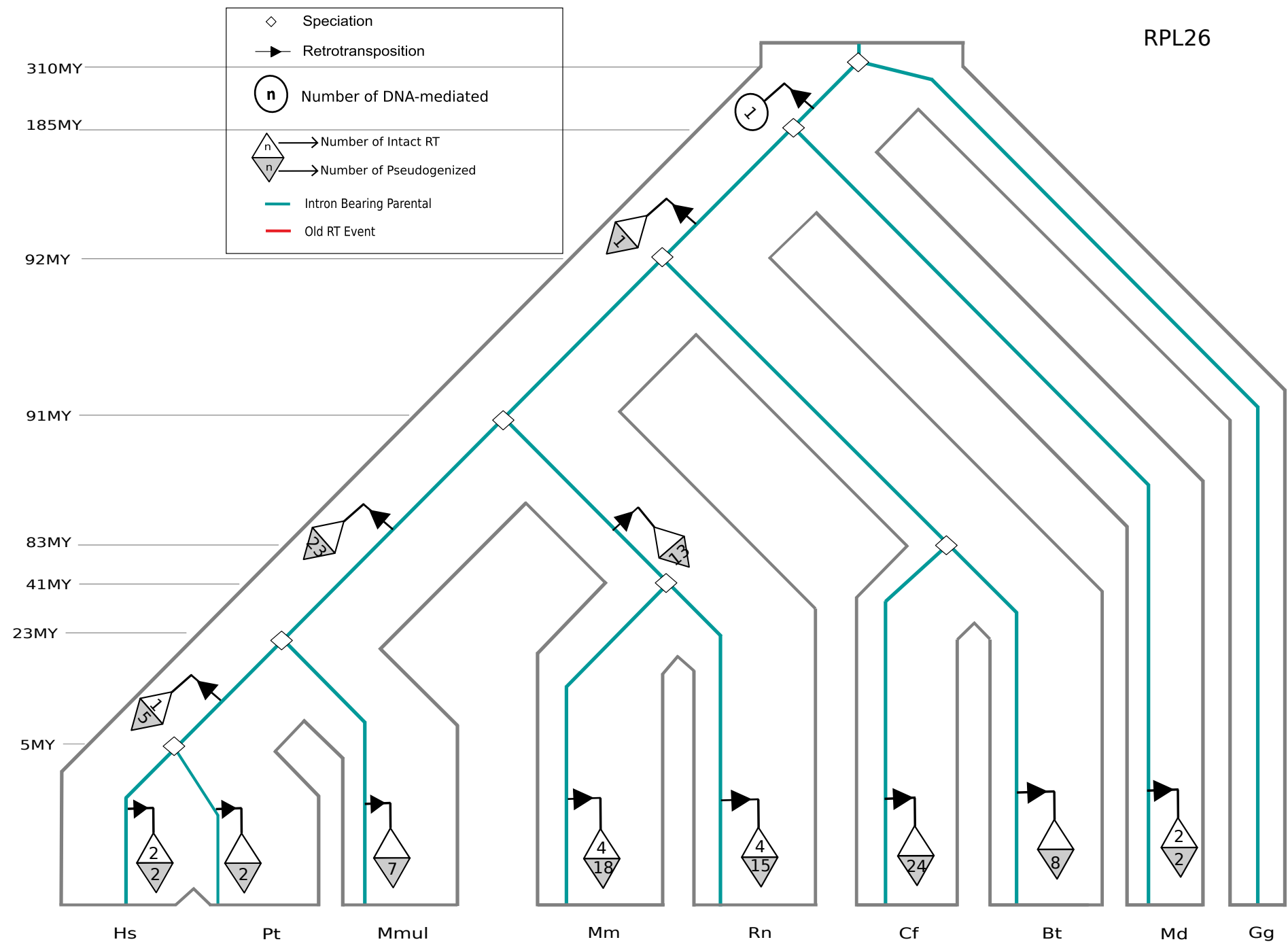

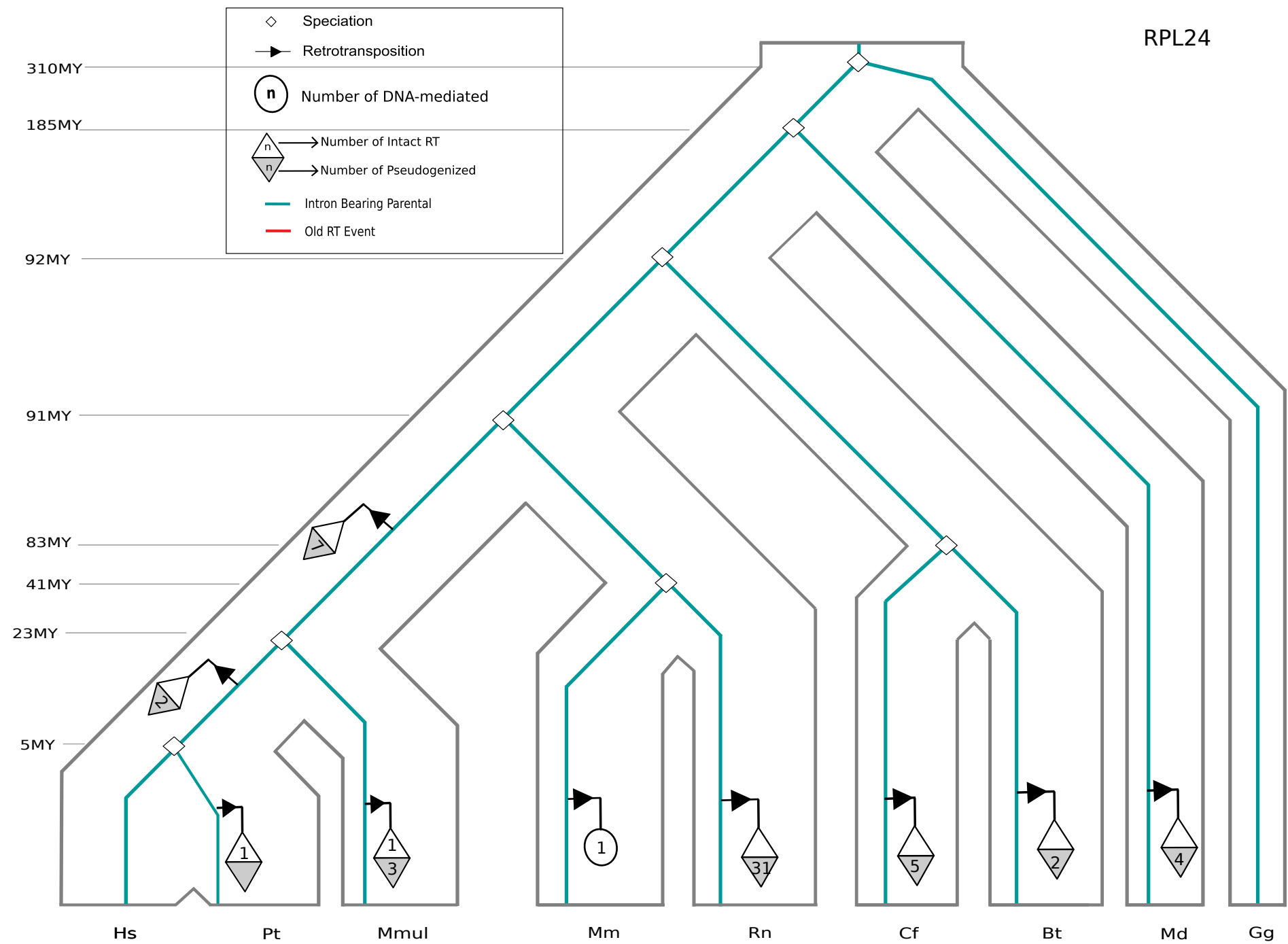

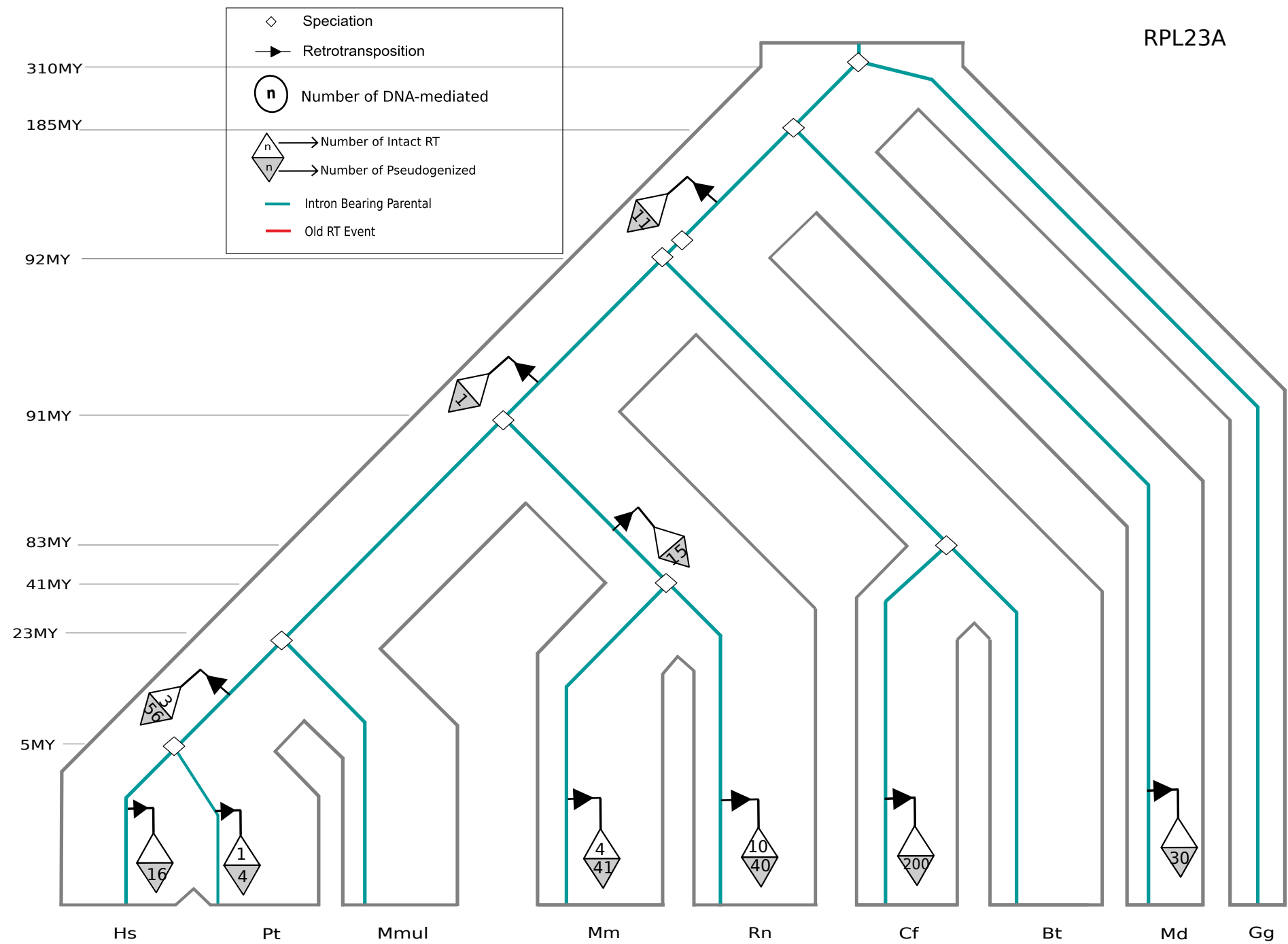

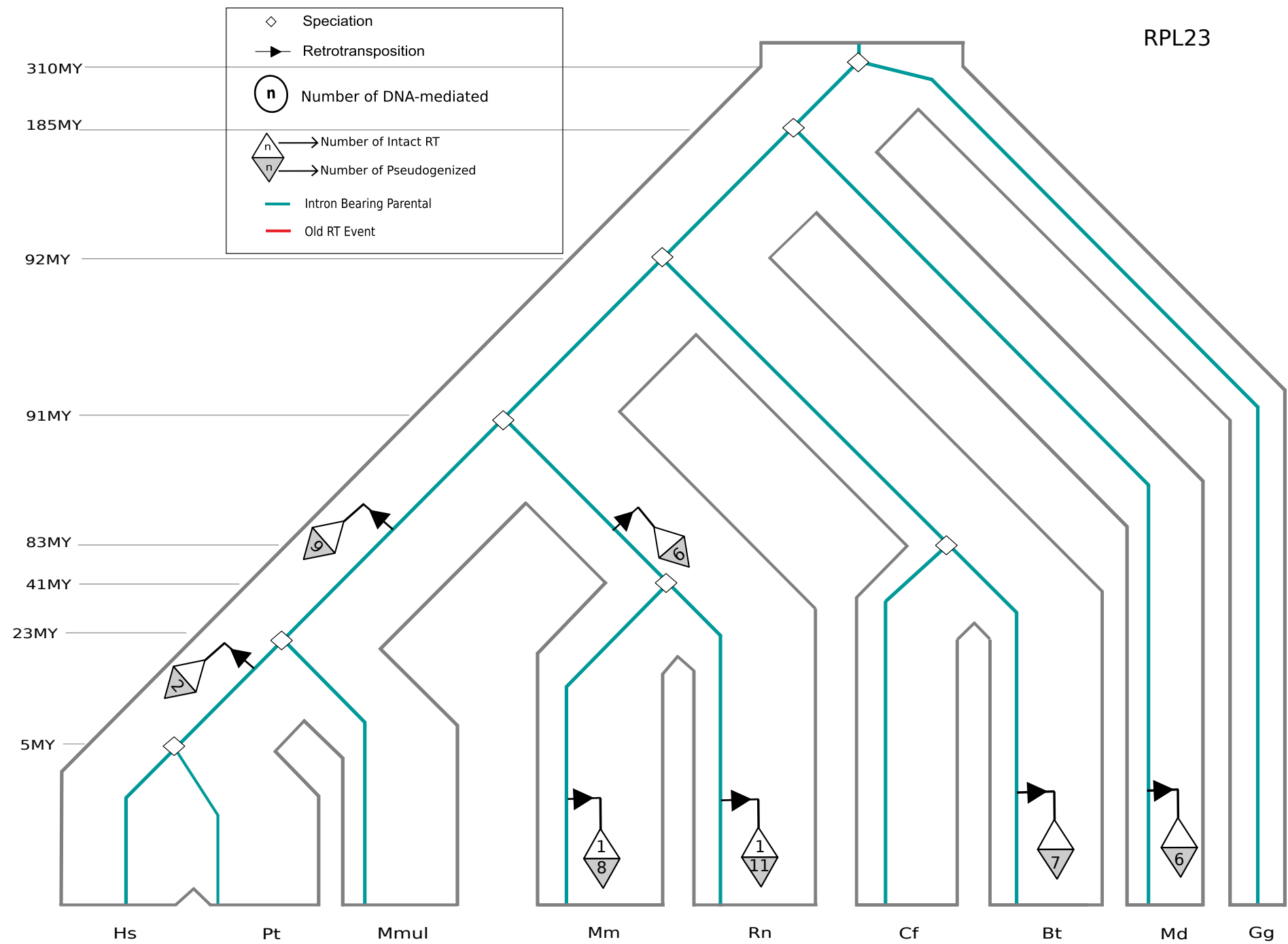

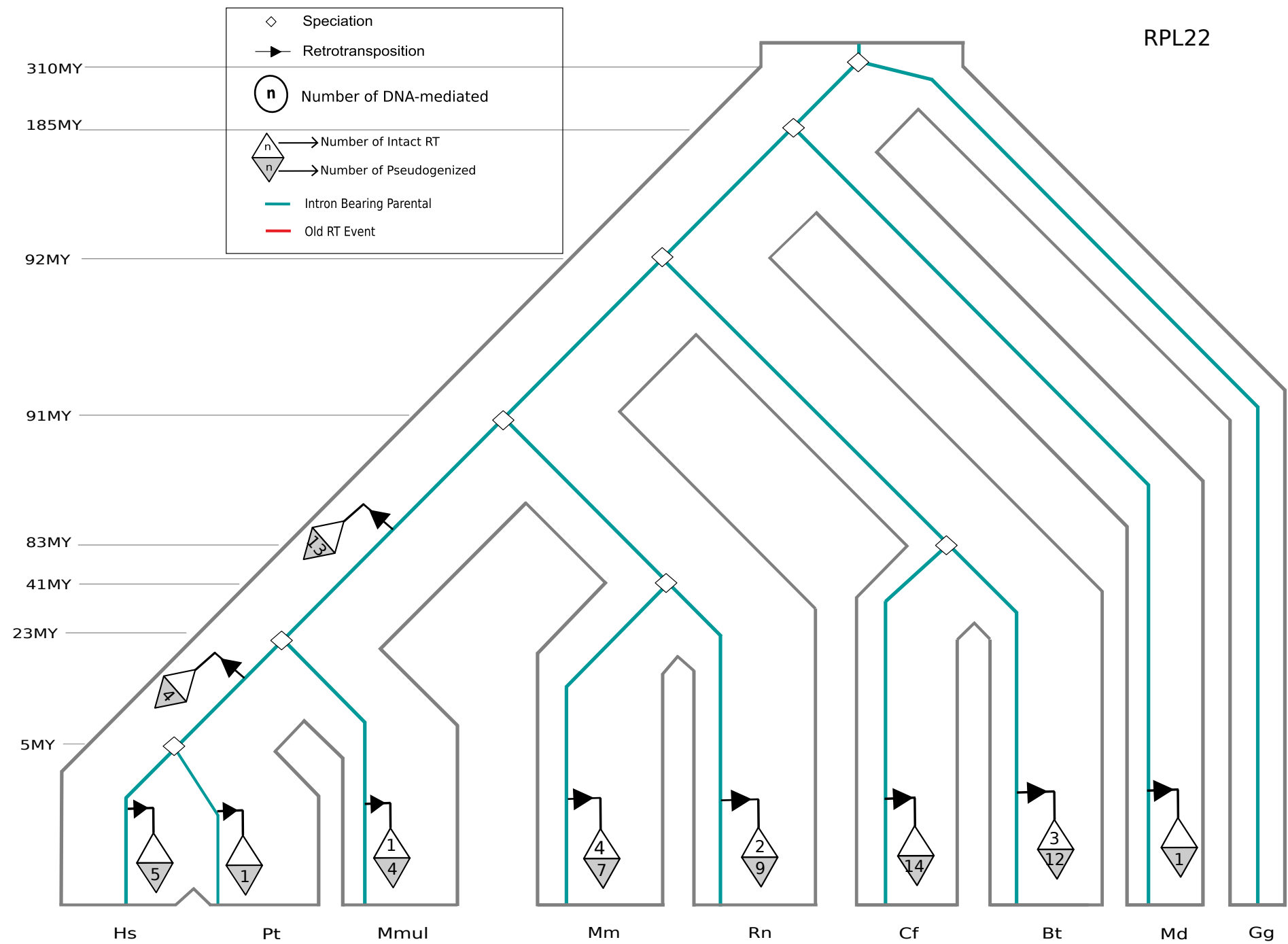

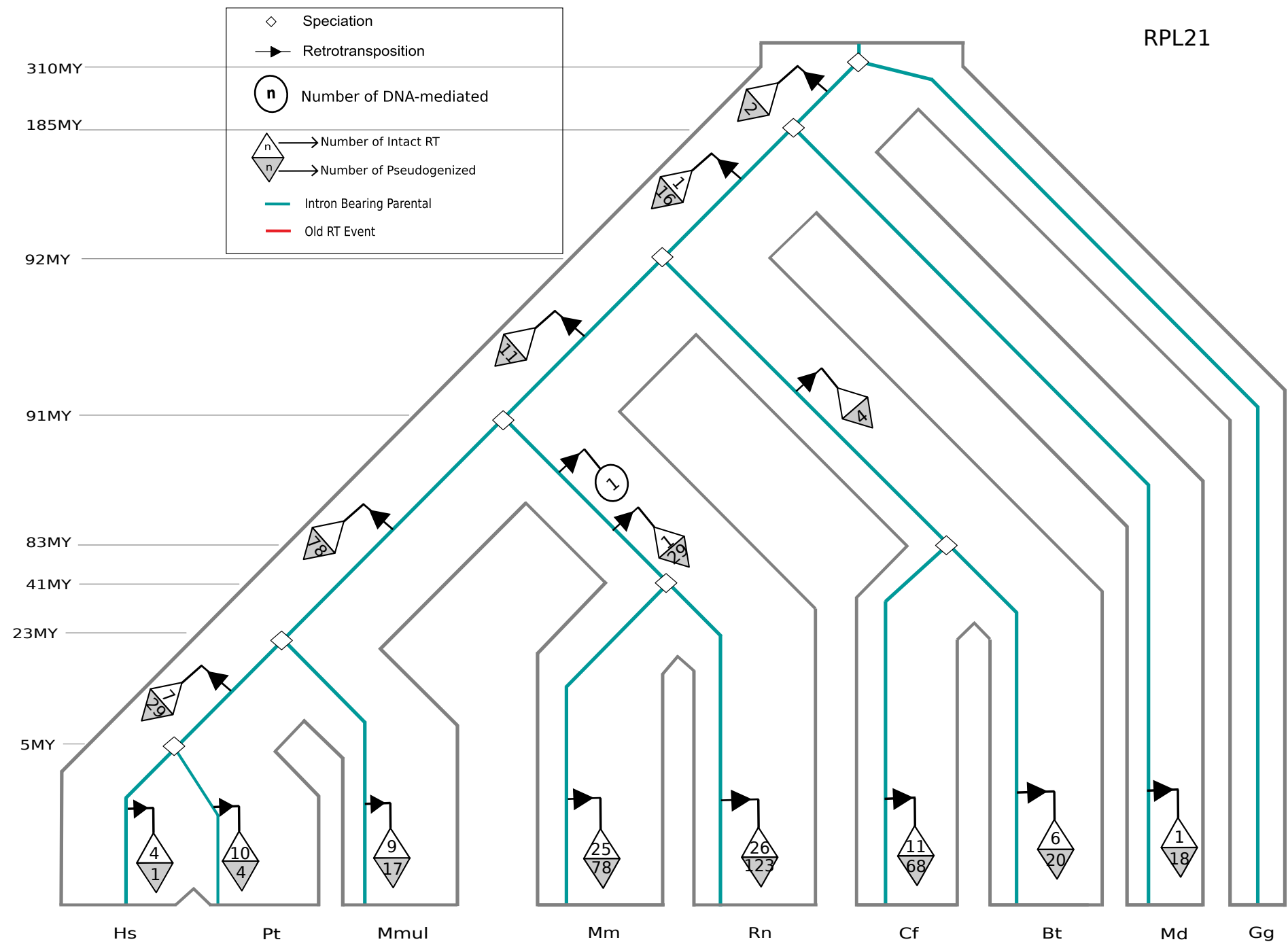

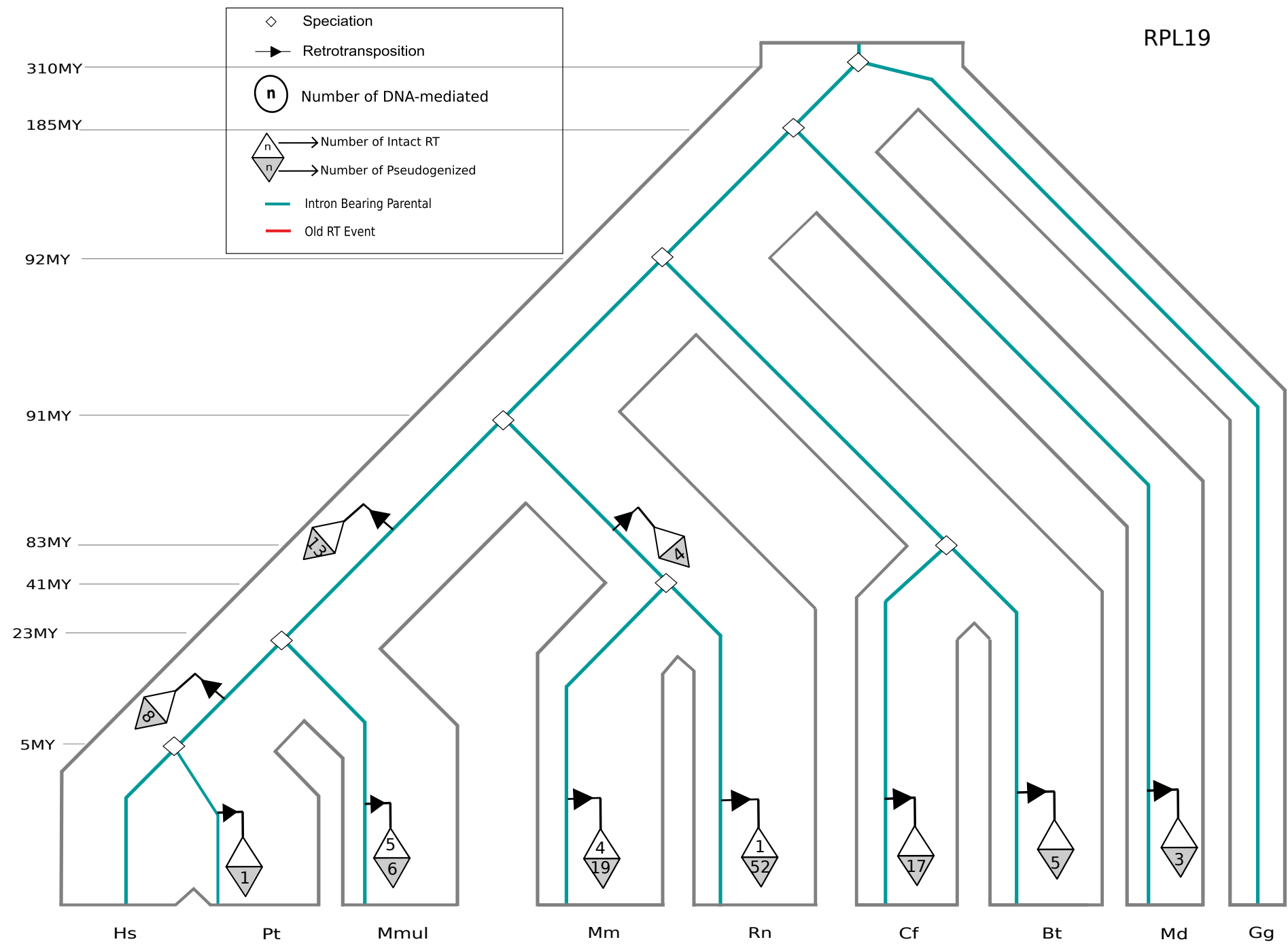

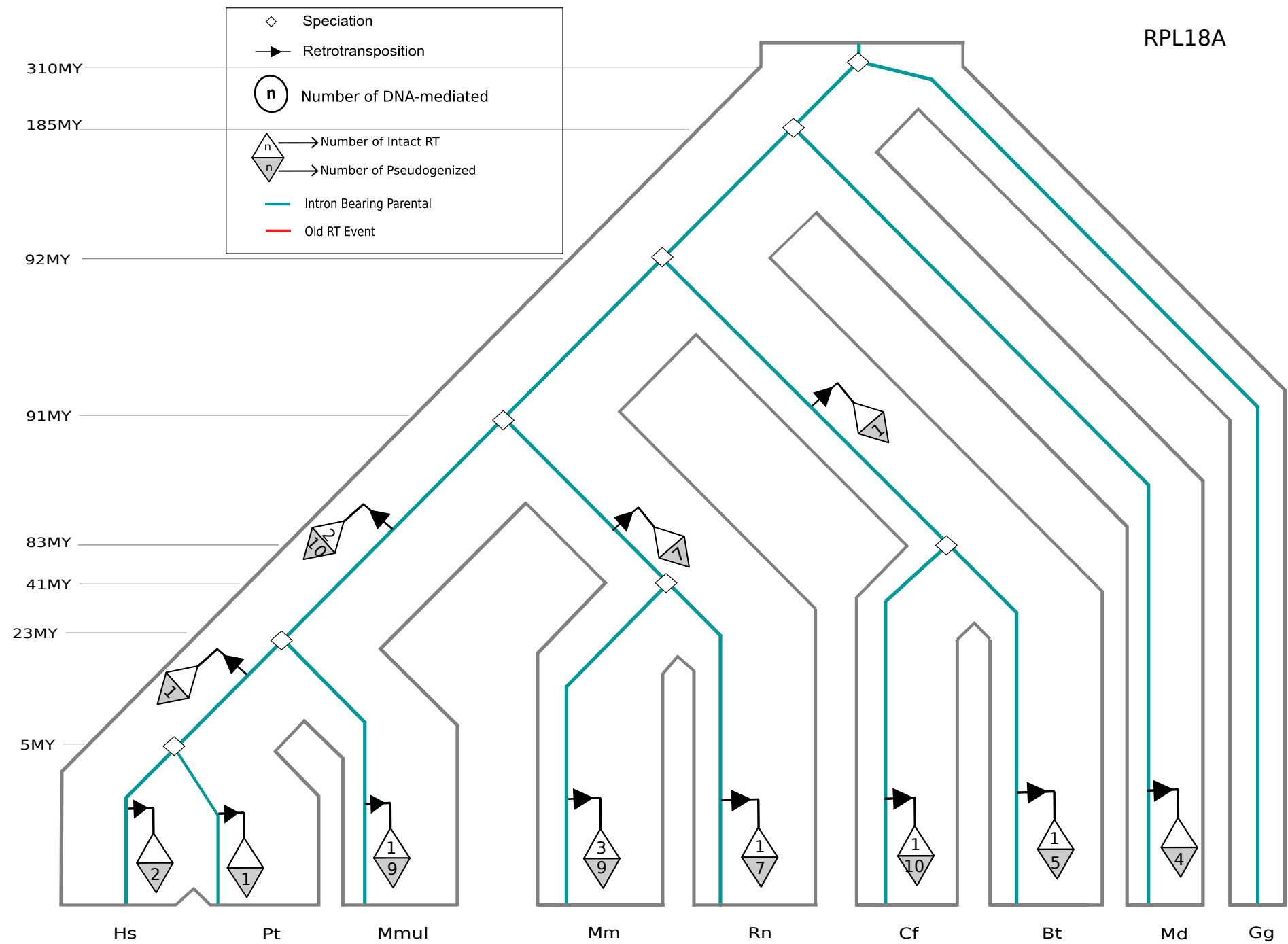

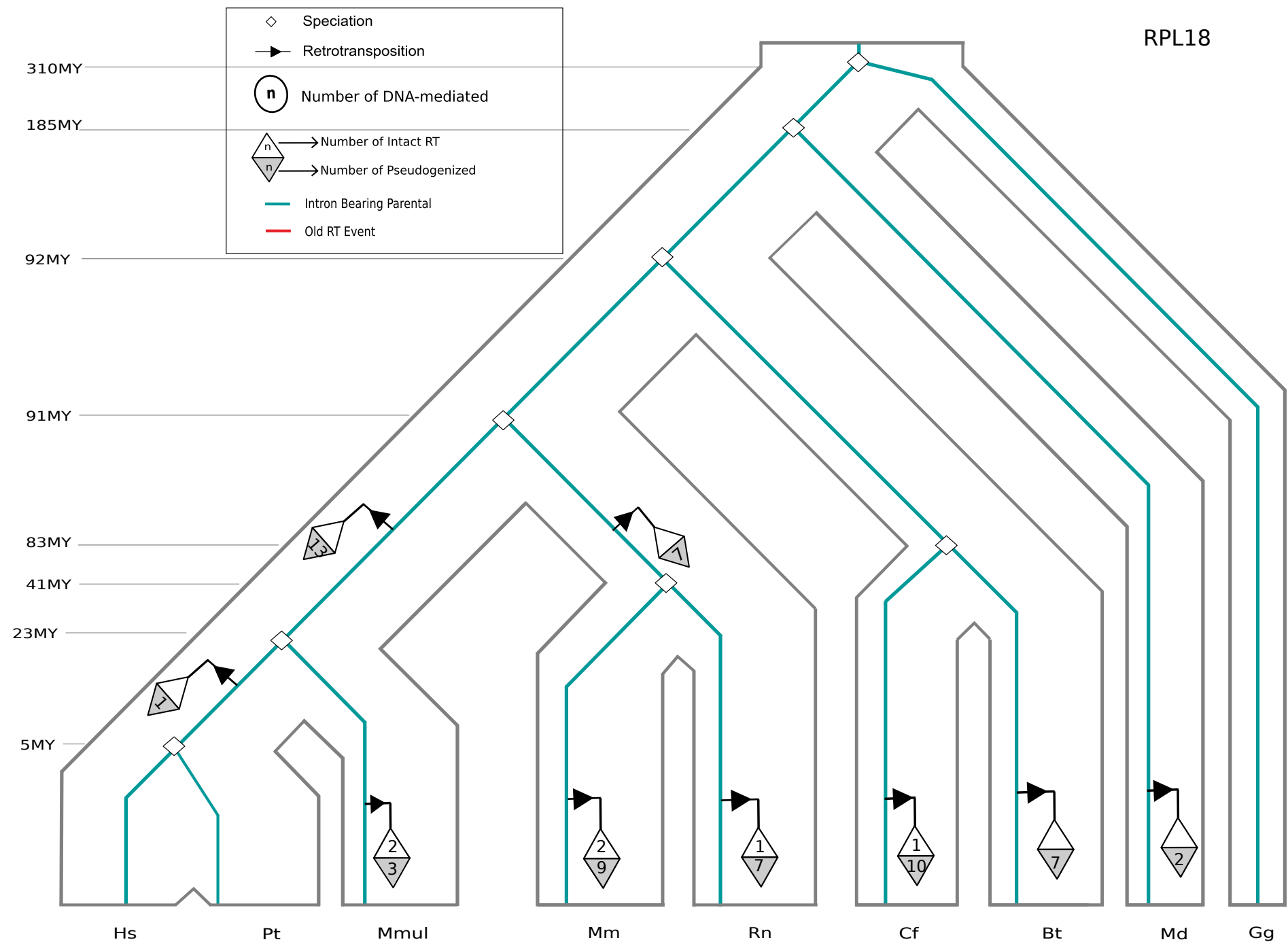

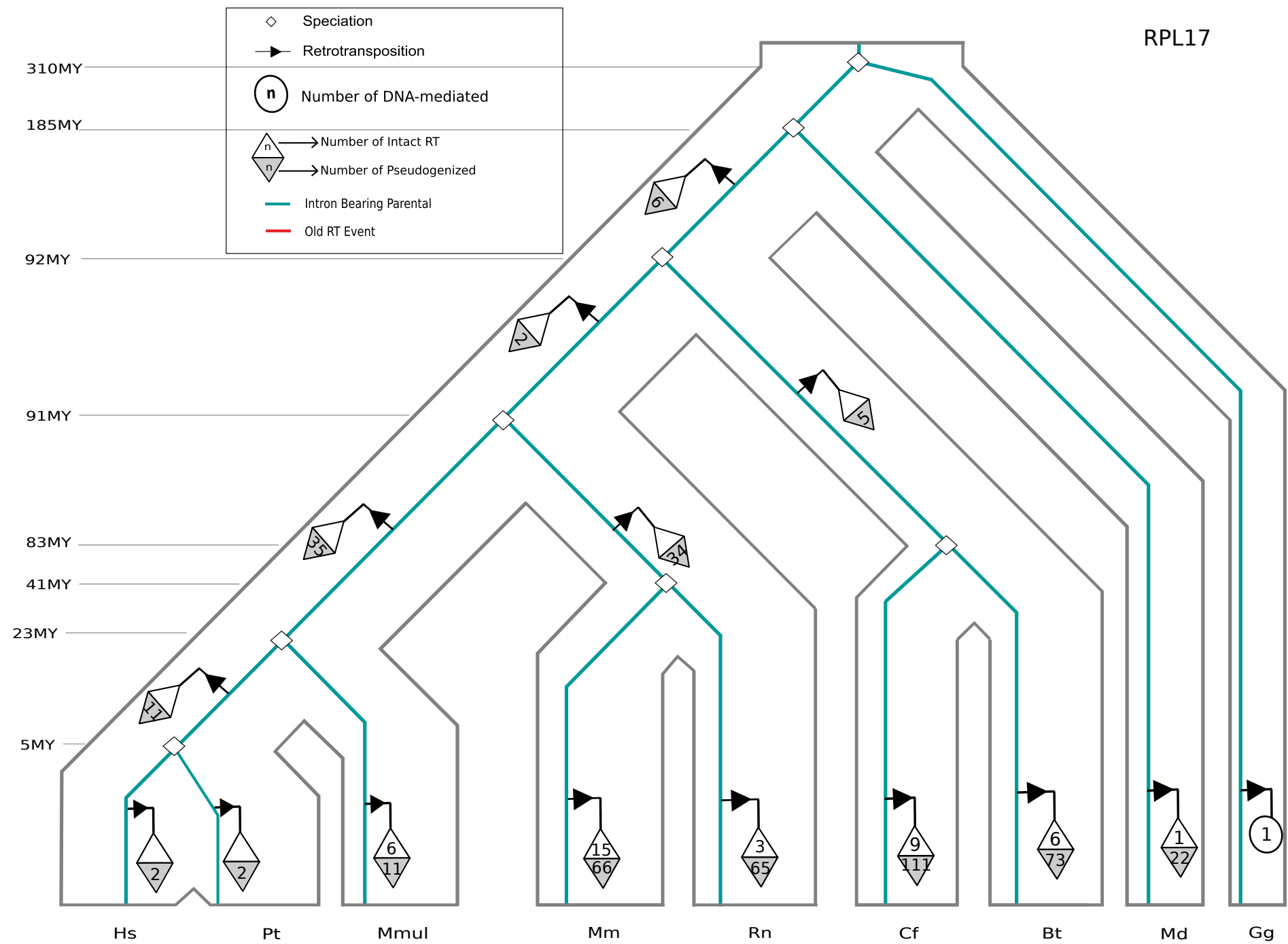

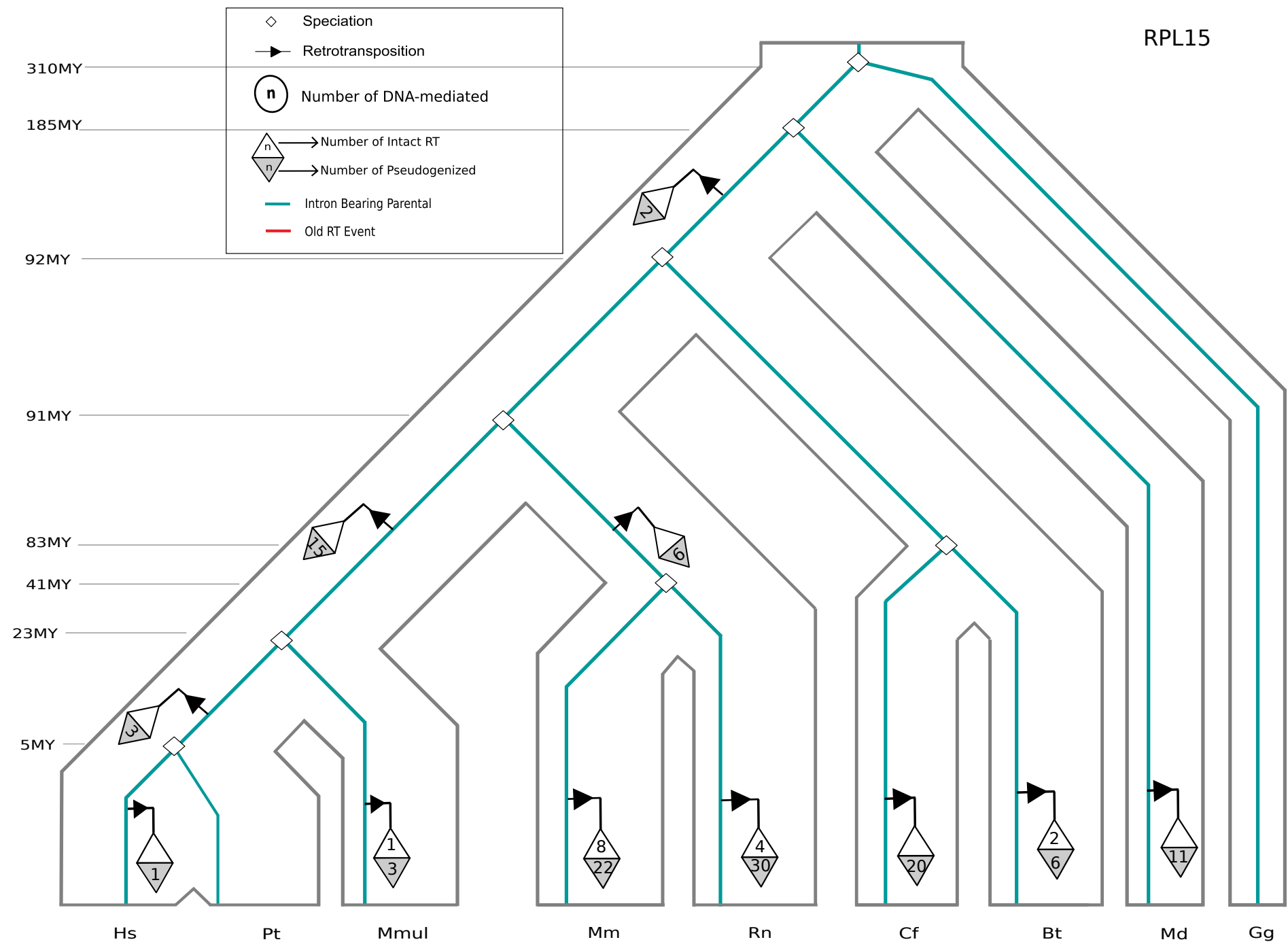

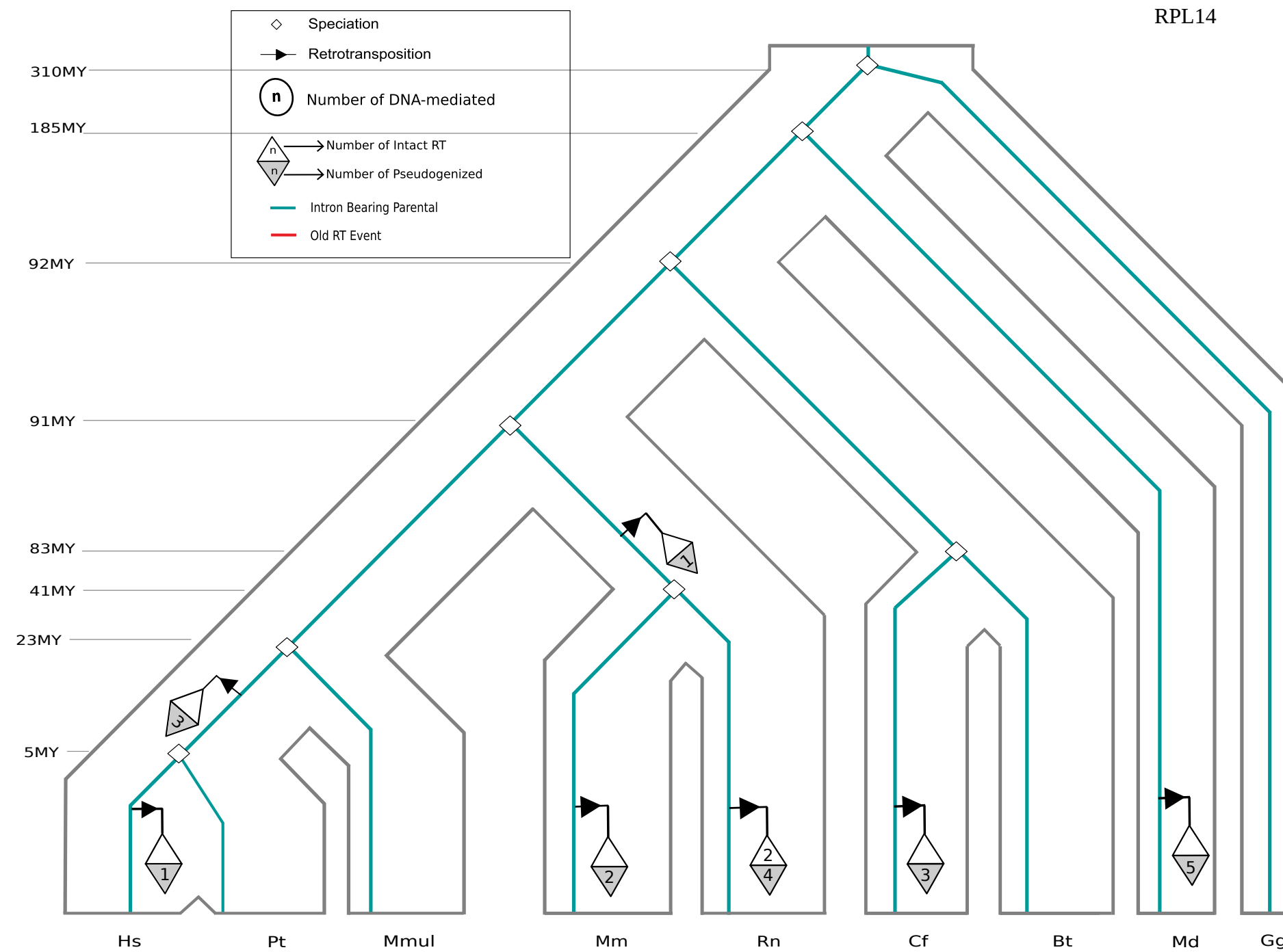

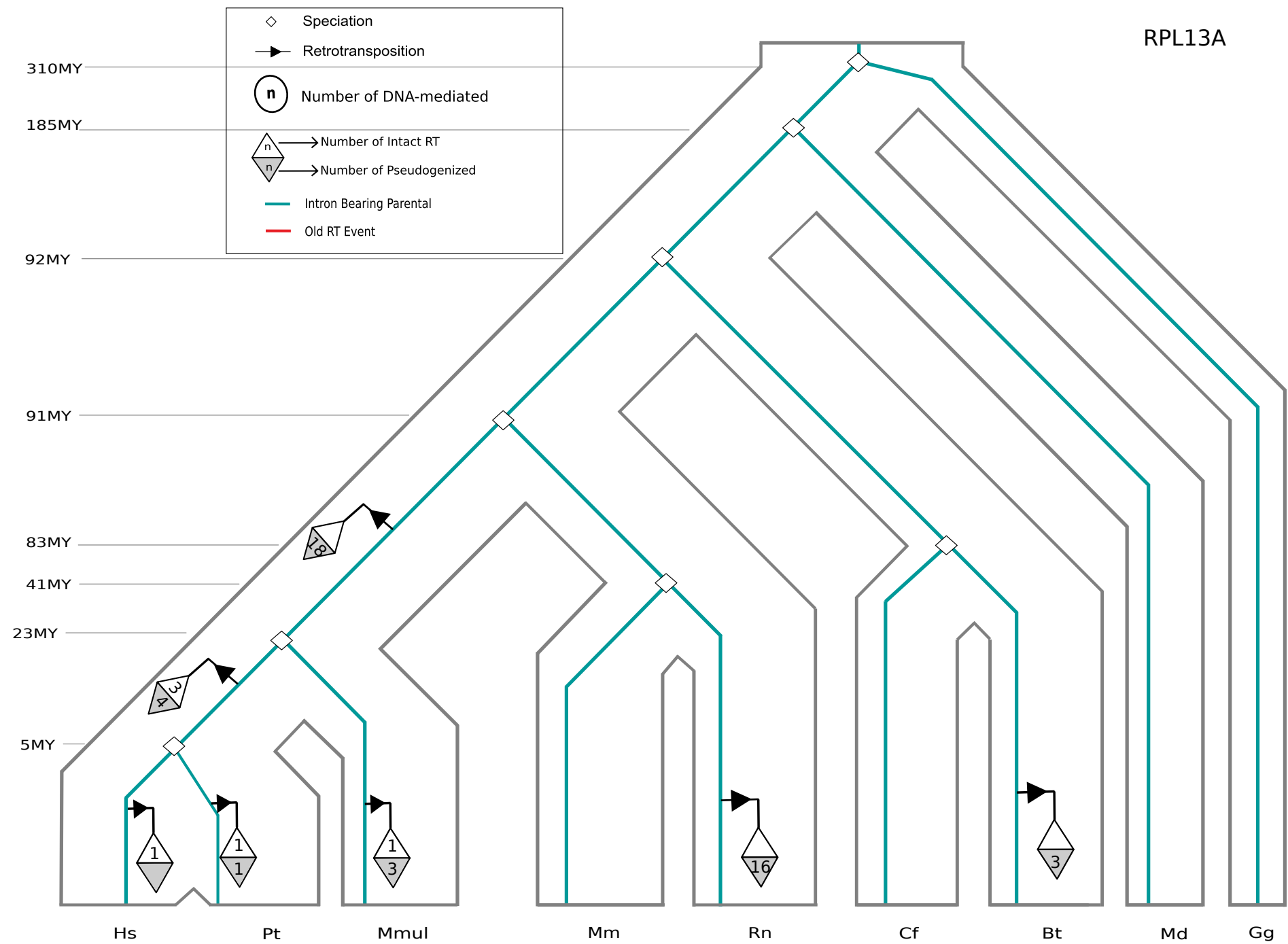

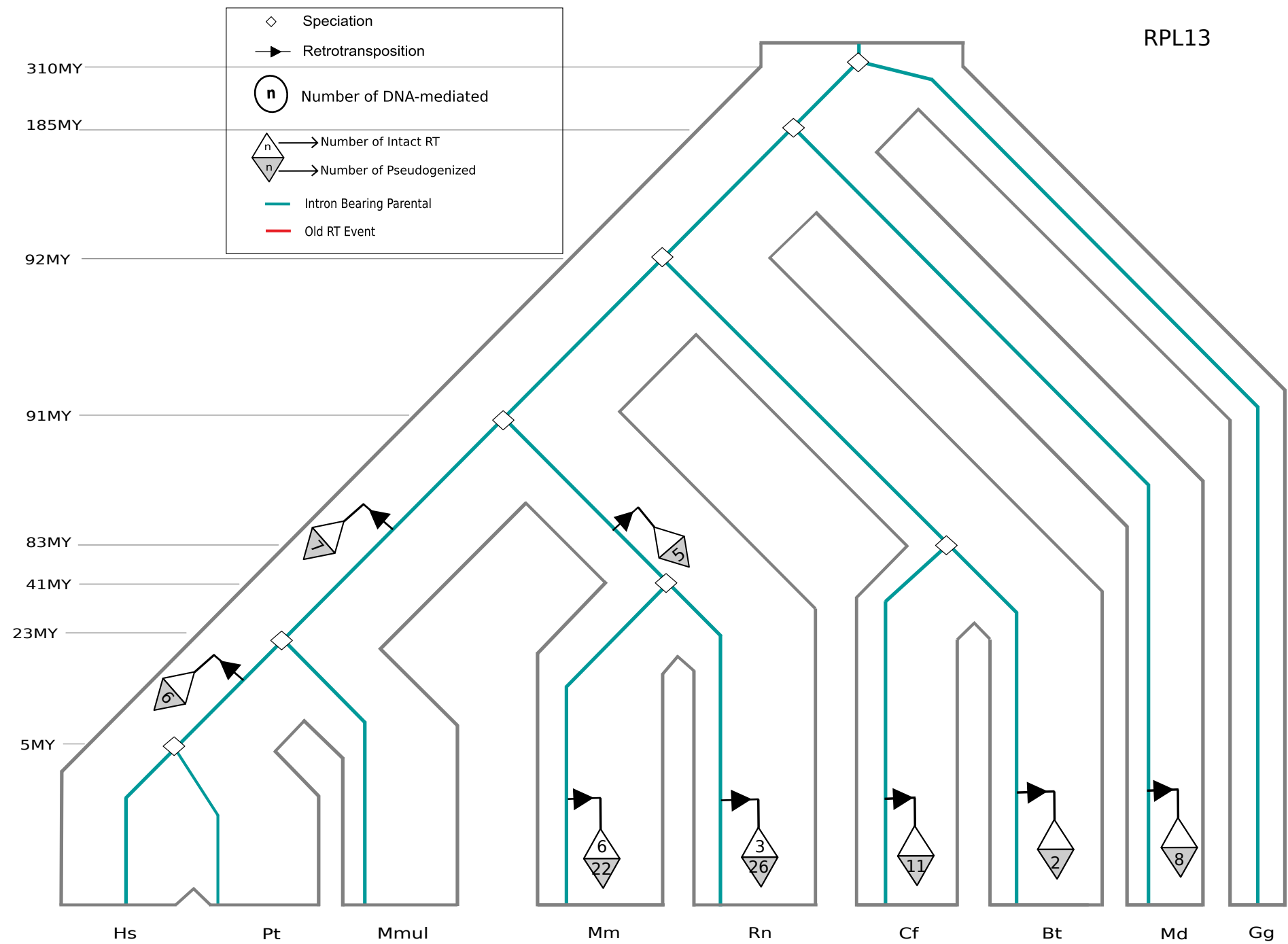

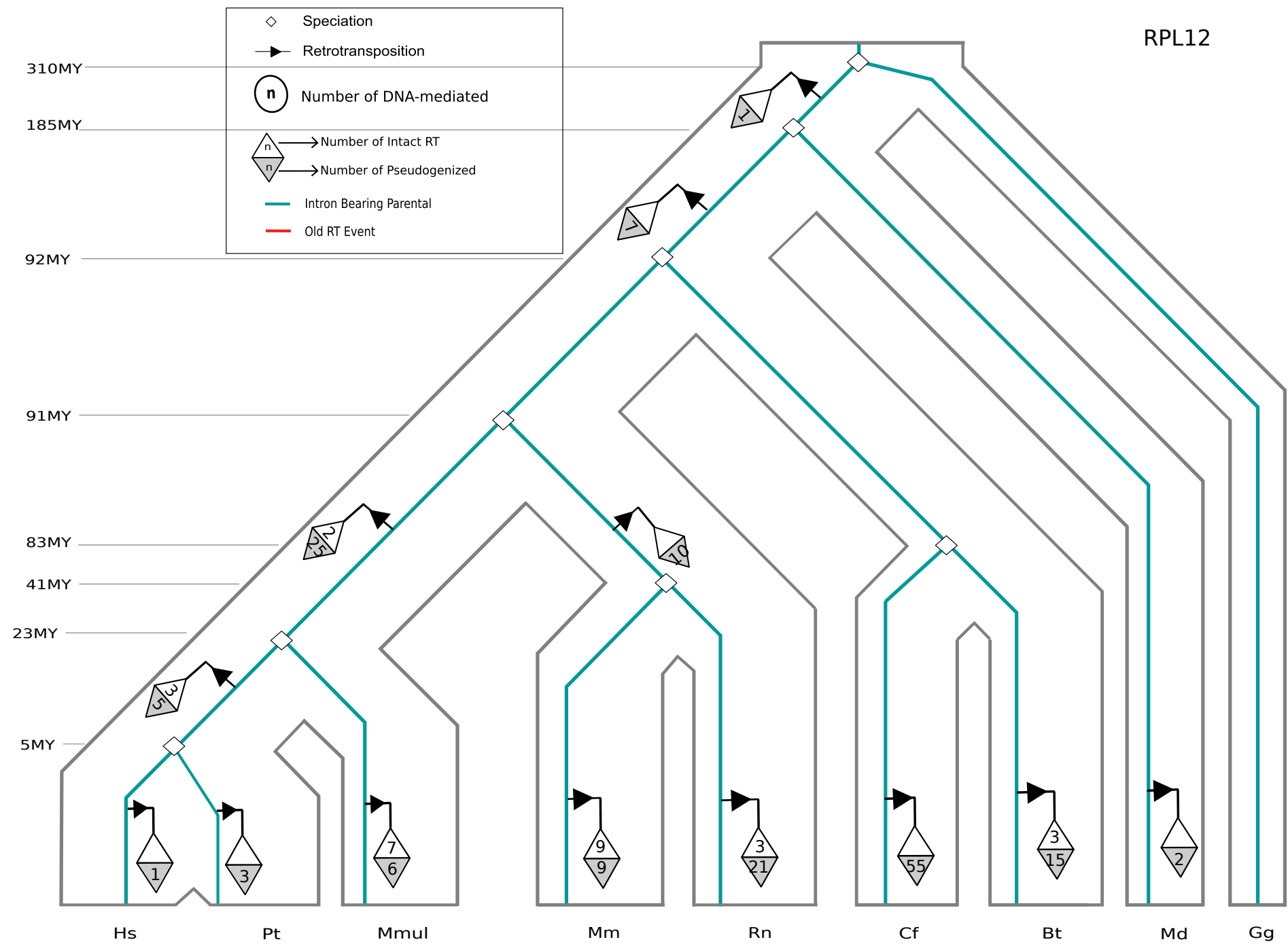

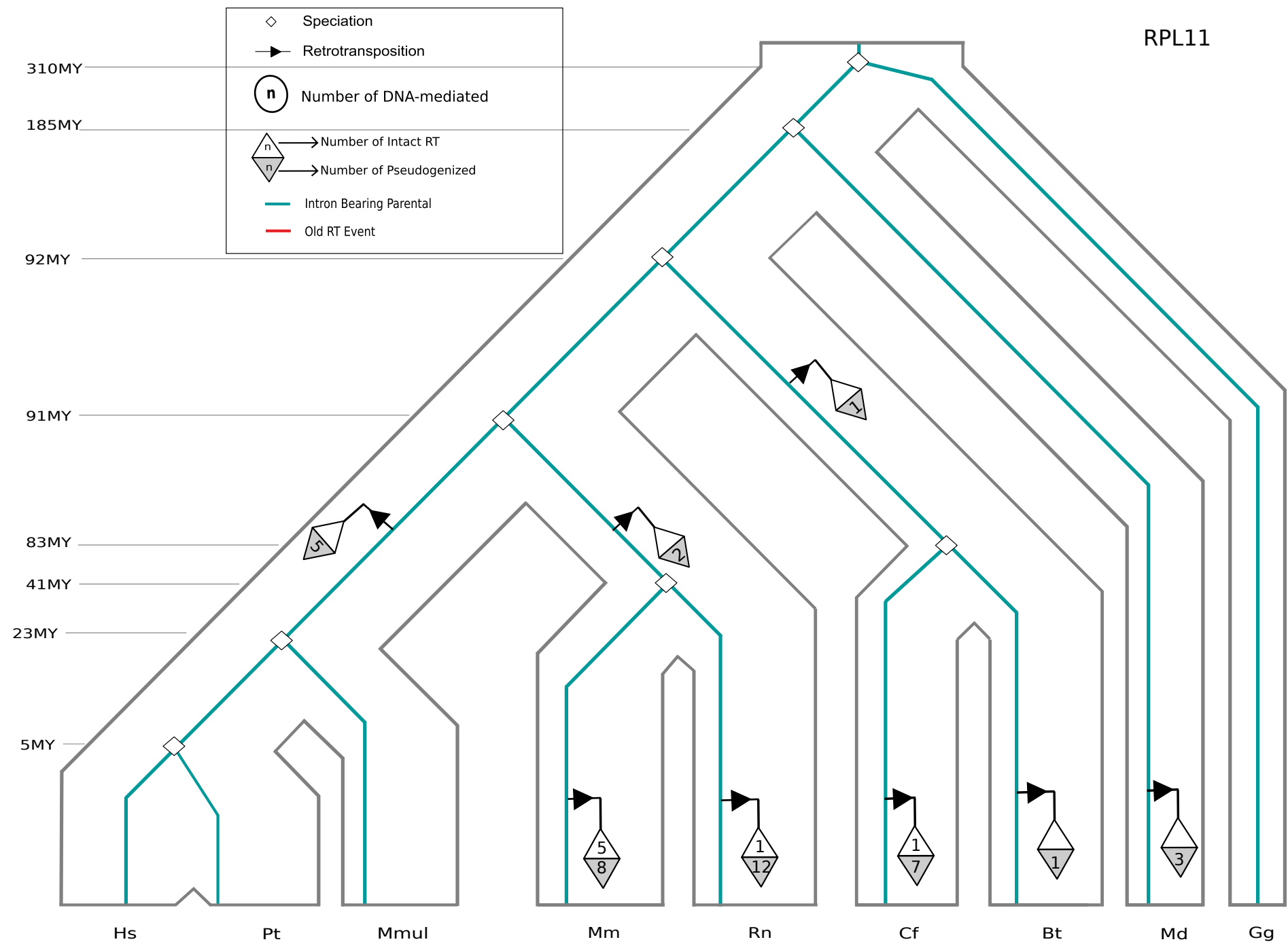

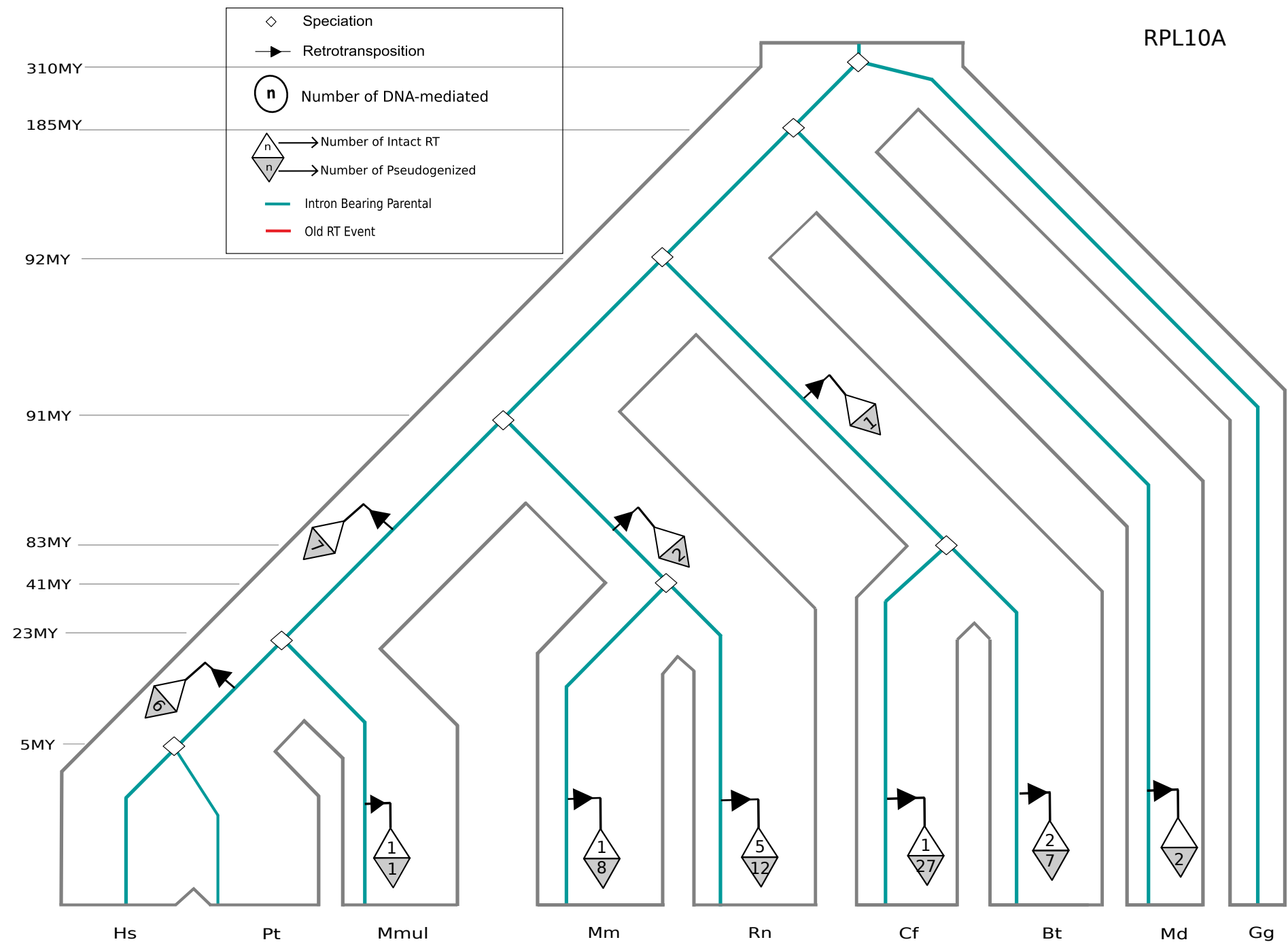

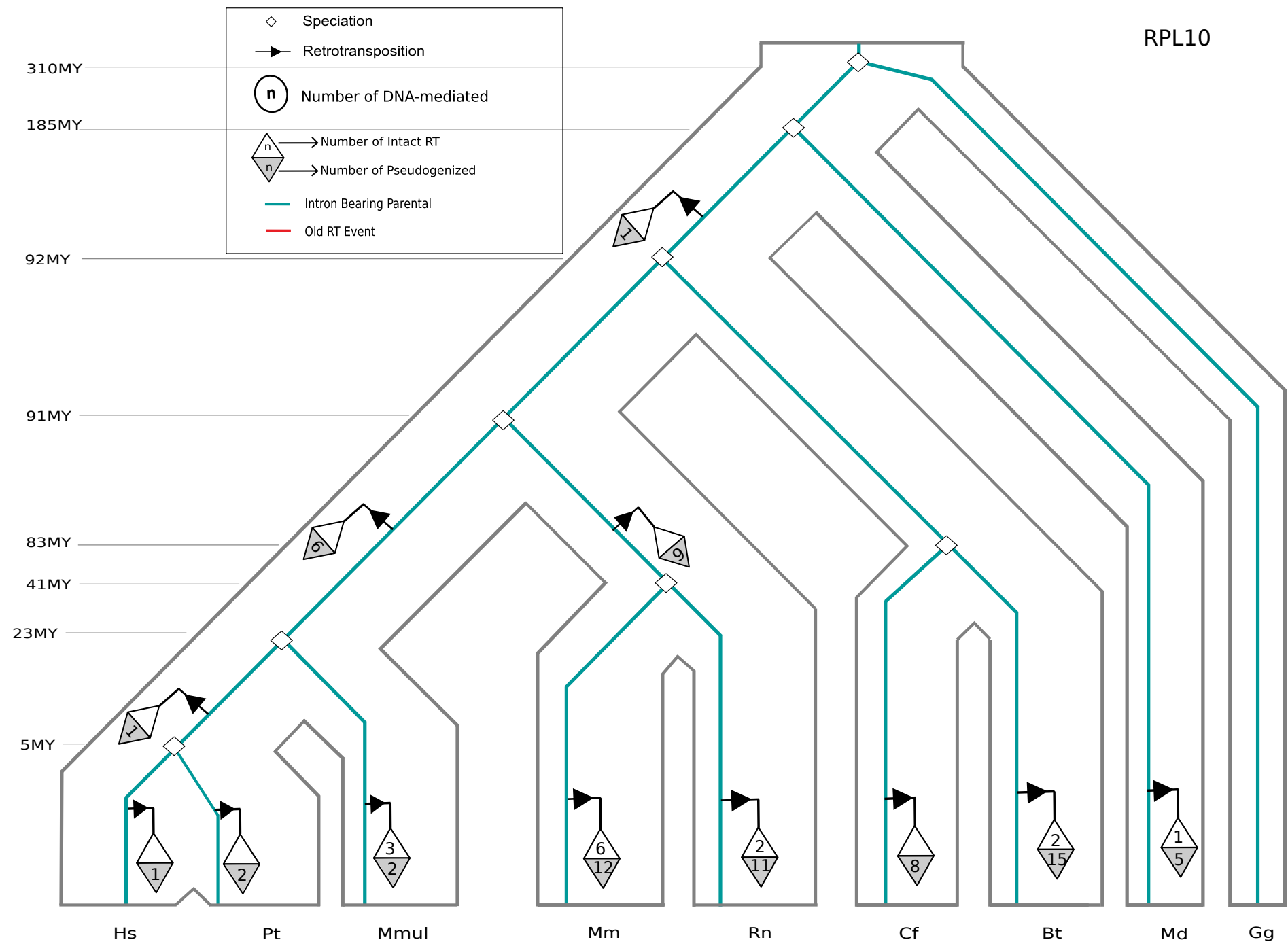

Supplement: Appendix S1 — 74 RP gene trees with all annotated duplication events. (PDF) [file pone.0111721.s011.pdf]
